# Introducing re-weighted range voting in clinical practice guideline prioritization: Development and testing of the re-weighted priority-setting (REPS) tool

**DOI:** 10.1371/journal.pone.0300619

**Published:** 2024-04-05

**Authors:** Michiel S. Oerbekke, Charlotte M. W. Gaasterland, Maarten J. van der Laan, Lotty Hooft

**Affiliations:** 1 Cochrane Netherlands, Julius Center for Health Sciences and Primary Care, University Medical Center Utrecht, Utrecht University, Utrecht, The Netherlands; 2 Knowledge Institute of the Dutch Association of Medical Specialists, Utrecht, The Netherlands; 3 Emma Center for Personalized Medicine, Emma Children’s Hospital, Amsterdam University Medical Centers, Amsterdam, The Netherlands; 4 Department of Surgery, University Medical Center Groningen, Groningen, The Netherlands; 5 Julius Center for Health Sciences and Primary Care, University Medical Center Utrecht, Utrecht University, Utrecht, The Netherlands; King Saud University Medical City, SAUDI ARABIA

## Abstract

We aimed to develop and test a tool based on the re-weighted range voting mechanism to prioritize items (i.e. key questions) in a priority-setting assessment for clinical practice guidelines. The secondary aim was to provide methodological context of the tool. We iteratively developed the tool and used qualitative methods (i.e. think-aloud and semi-structured interviews) to test the tool’s usability and make adjustments accordingly. An observational approach was used to test the tool’s outcome satisfaction in a real-world priority-setting assessment within a rare-disease guideline of a European Reference Network and under four different conditions in the tool. Four guideline methodologists tested the usability of the tool. The real-world testing was performed with a guideline panel consisting of a core working group, five expertise working groups, and a working group with patient representatives. Thirty-one panel members assigned scores in the priority-setting assessment. Seventeen panel members rated the priority-setting outcome, and sixteen panel members rated the outputs generated under the four conditions. Upon initial use, guideline methodologists found the tool to be quite overwhelming. However, with some initial effort they were able to easily identify the tool’s structure. Based on observations and feedback, the tool was further refined and user guidance was developed. Guideline panel members expressed (high) satisfaction with the priority-setting outcome. They particularly preferred the condition when using mean subgroup scores as input or employing aggressive penalties in the weighting method to determine the outputs. The tool generates a ranked list of items and offers flexibility for different choices in priority-setting assessments as long as its input format requirements are met. Although it is not a consensus method, the tool assists in narrowing down a set of priority items. Additional steps in the priority-setting assessment can lead to a consensus being reached regarding the final outcome.

## Introduction

Clinical practice guideline (CPG) developing organizations often cope with limited resources, which can result in a mismatch between the required work (e.g. updating) and the available capacity. Such limited resources may be a driver for the need to identify key items that require the allocation of resources above other items. Priority-setting assessments aim to help identifying such items (e.g. key questions, recommendations). By prioritizing these items, organizations can allocate their limited resources more effectively. Priority-setting is therefore a crucial process for CPG developing organizations who seek to optimize their resource utilization.

Multiple systematic reviews were conducted synthesizing priority-setting processes and indicators [1–3], including those related to the priority-setting of CPG topics [4], key questions in CPG development [5–7], CPG recommendations for uptake [8], and implementation [9]. Although it was stated that there is a need to develop standardized and validated priority-setting tools [5] and some methods have both been developed [6, 10, 11] and tested [7, 12, 13], it is important to recognize that different CPG developing organizations may have unique needs and operate in different contexts. No single published priority-setting assessment might therefore be fully generalizable to other CPG developing organizations without adaptation. Instead, it may be beneficial to develop methodological knowledge for priority-setting assessments and offer multiple options within priority-setting assessments. From here, organizations may choose steps, processes and indicators [1–3], and tools or methods [6, 7, 10–13] that best aligns with their purpose and context. That is, organizations may choose which steps (e.g. generation of topic list, collect data to inform discussions, use of prioritization criteria, ranking, refinement [1]) their assessment should contain, how these steps should be conducted (e.g. using stakeholder input or use existing CPGs to generate a topic list [1]), and which priority indicators (e.g. health burden [1, 2] or burden of disease [3], practice variation[3]) are deemed relevant. Such an approach could facilitate more effective utilization of the limited resources for CPG development within organizations due to the priority assessments being self-tailored to their own context, including their available resources to conduct the priority-setting assessment. Tools and methods suggesting a set of priority indicators may be at risk of offering indicators irrelevant to the context of organizations. For example, using ‘new available evidence’ and ‘consequences for costs’ as priority indicators may not necessarily seem to apply in dental care [7]. Furthermore, imposed steps and processes may cost resources (in a broad sense) that organizations might not be willing to invest or ask from participants. For example, for 107 questions rated by 30 participants on multiple indicators it took an average of 3.8 hours per participant [13] and thus an average total of 114 hours. In perspective, this is almost 10 percent of a 40-hour workweek for an individual participant and a total almost equaling the weekly productivity of three full-time employees. Some organizations might feel such a process is too time-intensive or puts too much strain on the participants and may wish to use a less resource-intensive process (e.g. less priority indicators to score, providing only one overall score, or even use a method without priority indicators).

Priority-setting assessments have relied on Delphi methods [4, 5, 8], counting [14], or calculating mean scores [6, 11, 13]. However, some contexts may need other options for initially ranking or selecting items. For example, results may be distorted when discussions involve persuasive actors or the composition of the participants is imbalanced. In a multidisciplinary oncology CPG, for example, there may be more representatives from internal medicine and surgery in the CPG panel than compared to pathology. This could lead to outcomes favoring the interests of the largest groups. However, pathology is critical for diagnosing and staging the disease to initiate a systemic and/or surgical treatment. New developments in this area may be important but not prioritized through counting or averaging due to imbalances in the representation. This could leave clinical guidance sub-optimal. Therefore, there is a need to explore additional options which could help less represented perspectives being considered in priority-setting assessments.

Re-weighted range voting uses a mechanism that adjusts the influence of participants for selecting the next item based on their scores on previously selected items [15]. This mechanism has a proportional representation characteristic [15], but can be modified to enhance the representation of less dominant perspectives in the ranking. This could depend on which data is used and the specific modifications made to the mechanism. However, before such tool can be actually used, it first needs to be developed and tested in real-world scenarios. Our goals were to develop a tool based on the re-weighted range voting mechanism (including a modified mechanism), to evaluate its usability, and to evaluate its outcome satisfaction in a real-world priority-setting assessment. Additionally, we aimed to provide methodological context for the tool to help CPG developing organizations determine whether our tool is appropriate for their priority-setting purpose and contextual requirements.

## Materials and methods

### Ethics statement

Review by a Medical Research Ethics Committee in the Netherlands is not required when the study is not under the scope of the Dutch Medical Research involving Human Subjects Act [16], where the current type of study does not meet the two conditions needed to fall under its scope [17].

### Frame of reference

A theoretical frame of reference was developed to discern key components of priority-setting assessments. Through a creative process, ideas pertaining to priority-setting assessments were integrated and adapted to generate abstract main components. The frame of reference will show the concept of sequential steps in a priority-setting assessment based on the generated main components and aimed to illustrate how we considered the role of our RE-weighted Priority-Setting (REPS) tool in priority-setting assessments to be. Such steps and their procedures may be dependent of the context and wishes of an organization. For example, an organization may choose to generate a topic list as an early step in their priority-setting assessment by surveying experts. To establish the initial face validity of the frame of reference, several priority-setting assessments published in the literature were aligned with its components.

### Development of the REPS-tool

#### Re-weighted range voting mechanism

Participants are instructed to assign scores from 0 (i.e. no priority) to the maximum scale score (i.e. highest possible priority; determined a priori). Participants also have the option to abstain from scoring an item, while negative scores are not allowed. The item with the highest total score is selected as the first winner [15]. The individual weight of participants are then adjusted based on their scores assigned to previously selected winners, using Formula 1 [15, 18]:

$$individual\;weight=\;\frac{constant}{\left(constant+\;\frac{sum\;of\;participant^{\prime }s\;scores\;on\;ranked\;winners}{maximum\;scale\;score}\right)}$$ (1)

The participant’s scores on the remaining items are multiplied by their respective individual weights and the new scores are summed for each item. The highest total score is subsequently identified as the next winner. We additionally used a second weighting method to achieve a more disproportionate representation by introducing a parameter (here denoted as *A*) as an exponent of the participant’s sum of scores on ranked winners (Formula 2) [15]. We will refer to this method as the decay-adjusted weighting method, and to the exponent as decay aggression (*A*):

$$individual\;weight=\;\frac{constant}{\left(constant+\;\frac{sum\;of\;participant^{\prime }s\;scores\;on\;ranked\;winners^{A}}{maximum\;scale\;score}\right)}$$ (2)

#### Early usability testing

Think aloud sessions [19] with semi-structured interviews were performed with CPG methodologists, who were invited in June-July 2020 and agreed to participate. The methodologists participated in individual video calls where they utilized a preliminary version of the REPS-tool. The methodologists were informed about the purpose, the session, the recording (including the anonymized transcription), and were provided with the opportunity to ask any questions (see S1 File for the protocol). The methodologists were asked to share their screen and communicate their thoughts out loud while they were performing tasks in the REPS-tool during the session. One author (MSO) led the one-to-one sessions and recorded the video call for later analysis. The recorded videos were transcribed word-by-word in Dutch and enriched with descriptions of the methodologists’ on-screen actions. One author (MSO) analyzed the transcriptions by hand and added qualitative labels to observed experiences in Dutch (labels were also translated to English whilst quotes remained in Dutch). The observations were thereafter organized in clusters based on the different phases in the think aloud sessions, forming the basis for subsequent interpretations.

#### Real-world priority-setting

**Fig 1:**
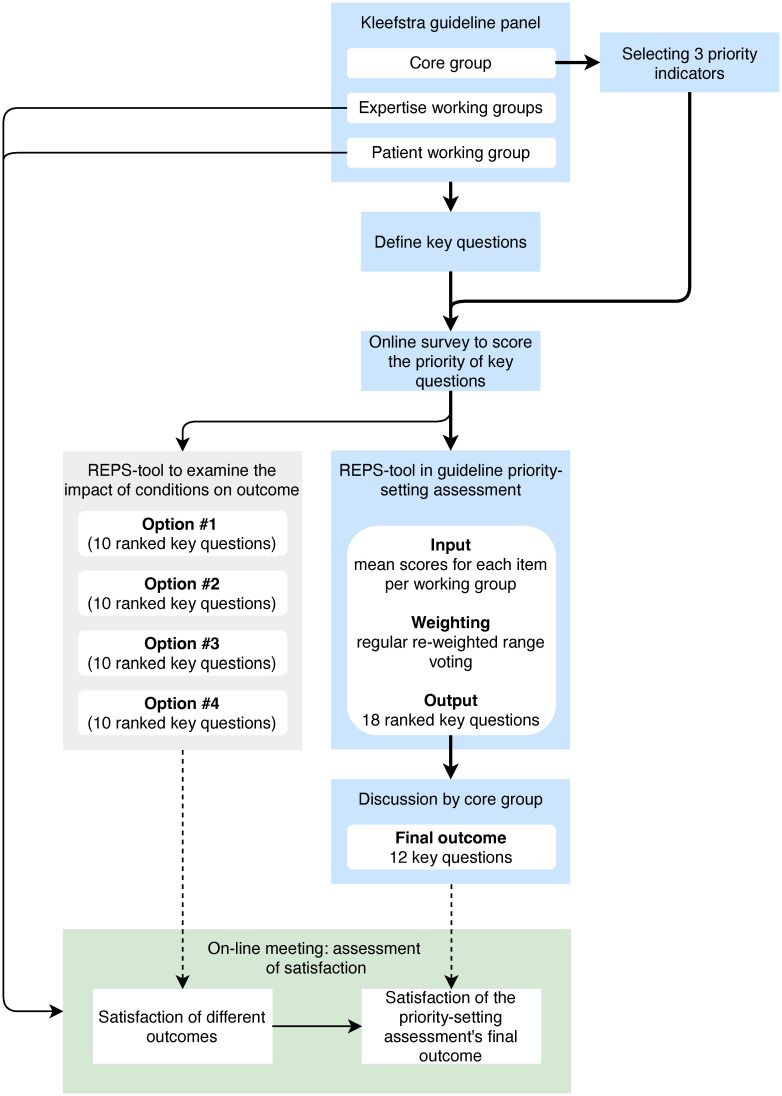
Flow of the priority-setting assessment pilot. The figure shows the process of the priority-setting assessment in the Kleefstra guideline (blue boxes and bold arrows). The assigned scores in the survey were also used to test four different conditions in the REPS-tool determining the outcome (grey box). Satisfaction of the four different outcomes of the REPS-tool (from the grey box) and the final outcome of the Kleestra priority-setting assessment (from the blue process) were assessed in an online meeting (green box).

We used the REPS-tool in the priority-setting of key questions in the European Reference Network on Rare Congenital Malformations and Rare Intellectual Disability (ERN-ITHACA) rare-disease CPG concerning the Kleefstra syndrome (Fig 1). There were resources to newly develop 12 out of 45 key questions defined by the CPG panel. The panel consisted of a core group (including a CPG methodologist), five working groups (based on expertise), and a working group of patient representatives. Three priority indicators were selected by the core working group: 1) unwanted (international) clinical variance that may be improved with the guideline, 2) high prevalence of symptoms, and 3) high (disease) burden for either the Kleefstra population or for their relatives. Panel members were invited to an online survey and were asked to individually provide an overall priority score ranging from 0 to 5 per key question based on the three indicators. The mean scores for each key question within each working group were entered into the REPS-tool. The regular re-weighted range voting (Formula 1) was used as the weighting method. Thereafter, the resulting top-18 rankings were discussed by the CPG panel’s core group. This led to the selection of 12 key questions as the final outcome of the priority-setting assessment.

The set of priority scores from the survey was also used to generate top-10 outputs of the REPS-tool, considering four different conditions in order to examine their impact on the outcome: 1) mean scores from the working group while using the regular re-weighted range voting, 2) individual participant scores while using the regular re-weighted range voting, 3) individual participant scores while using a low decay aggression, and 4) individual participant scores while using a high decay aggression. During an online meeting, the CPG panel members (excluding the core group) individually indicated their preferred top-10 outputs through a poll. CPG panel members were thereafter presented with the core group’s selection of 12 key questions and were asked to express their satisfaction level with this final outcome of the priority-setting assessment (ordinal poll with five response levels: very content, content, medium, not very content, and dissatisfied).

## Results

### Frame of reference

**Fig 2:**
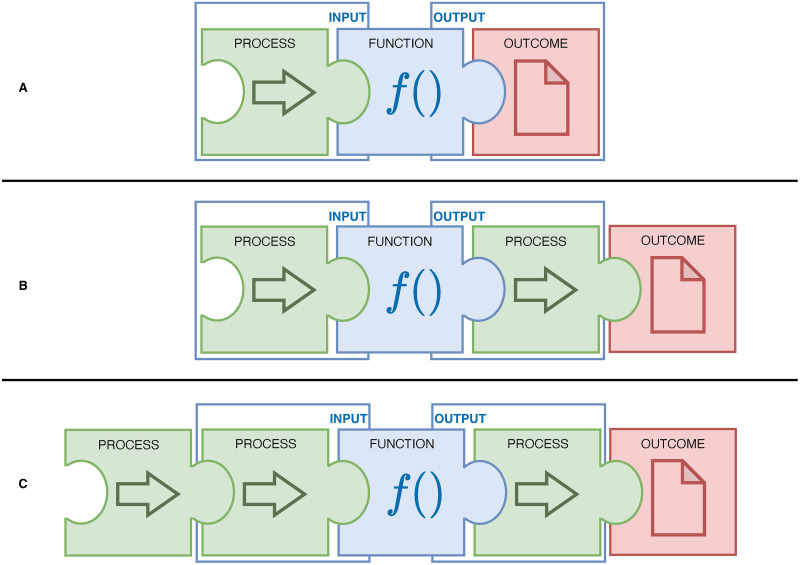
Priority-setting assessment components. The figure shows three main components of a priority-setting assessment: process, function, and outcome. In a basic form, a single process component leads to input for the function which produces an output as the final outcome of the priority-setting assessments (Fig 2A). Priority-setting assessments can be extended by incorporating additional process components tailored to the specific needs of an organization. For example, adding a process component to discuss the function’s output to determine the final priority-setting outcome (Fig 2B and 2C). Alternatively, multiple process components can be introduced before the function component (e.g. to select participants and to identify relevant priority-indicators) leading to the function’s input (Fig 2C).

Three main components in priority-setting assessments were discerned (Fig 2): the process component, the function component (including its input and output), and the outcome component. The process component involves procedural steps followed in the priority-setting assessment. The function component contains specific rules and/or calculations that determine the priority of items. It requires an input to perform its function, and results in an output. The outcome component represents the final outcome of the priority-setting assessment. It can directly correspond to the output of a function (Fig 2A), or additional process components are added to achieve a final outcome of the priority-setting assessment (Fig 2B and 2C). The initial face validity of the frame of reference was examined by aligning priority-setting assessments from the literature to its components (S2 File).

### Early usability testing

All four participating CPG methodologists who participated were female, with their ages ranging from 26 to 32 years old. Their overall working experience varied from 3 to 8 years, with 1 to 3 years specifically in CPG development. Among the methodologists, three held a PhD degree and one an MSc degree. Three of the methodologists had experience with priority-setting in some way, of which one of them had seen the preliminary tool previously before participating in the usability test.

The first impression of the REPS-tool was that it was large and complex. The tool’s structure concerning the placement of scores and ranks was initially unclear. Participants became familiar with the structure after some experimenting, through performing the assignments, or some verbal instructions. Once the structure was clear, we observed that the participants placed the input data correctly in the tool and could get the output out of the tool. Participants generally thought the automated functions in the tool were convenient and wished for more automation in other parts of the tool, for example, one participant was missing a feature that would check for valid scores. The manual ranking process was more inconvenient when there were a lot of items in the tool. This was mainly due the difficulty of searching for the identified winner in the worksheet and remembering which rank to assign to the winner. Furthermore, the participants preferred to have some background information on the tool’s mechanism and receiving guidance about how to use the decay aggression effectively. The qualitative analysis and all actions to improve based on this usability testing can be found in S3 File.

### The REPS-tool function component

**Fig 3:**
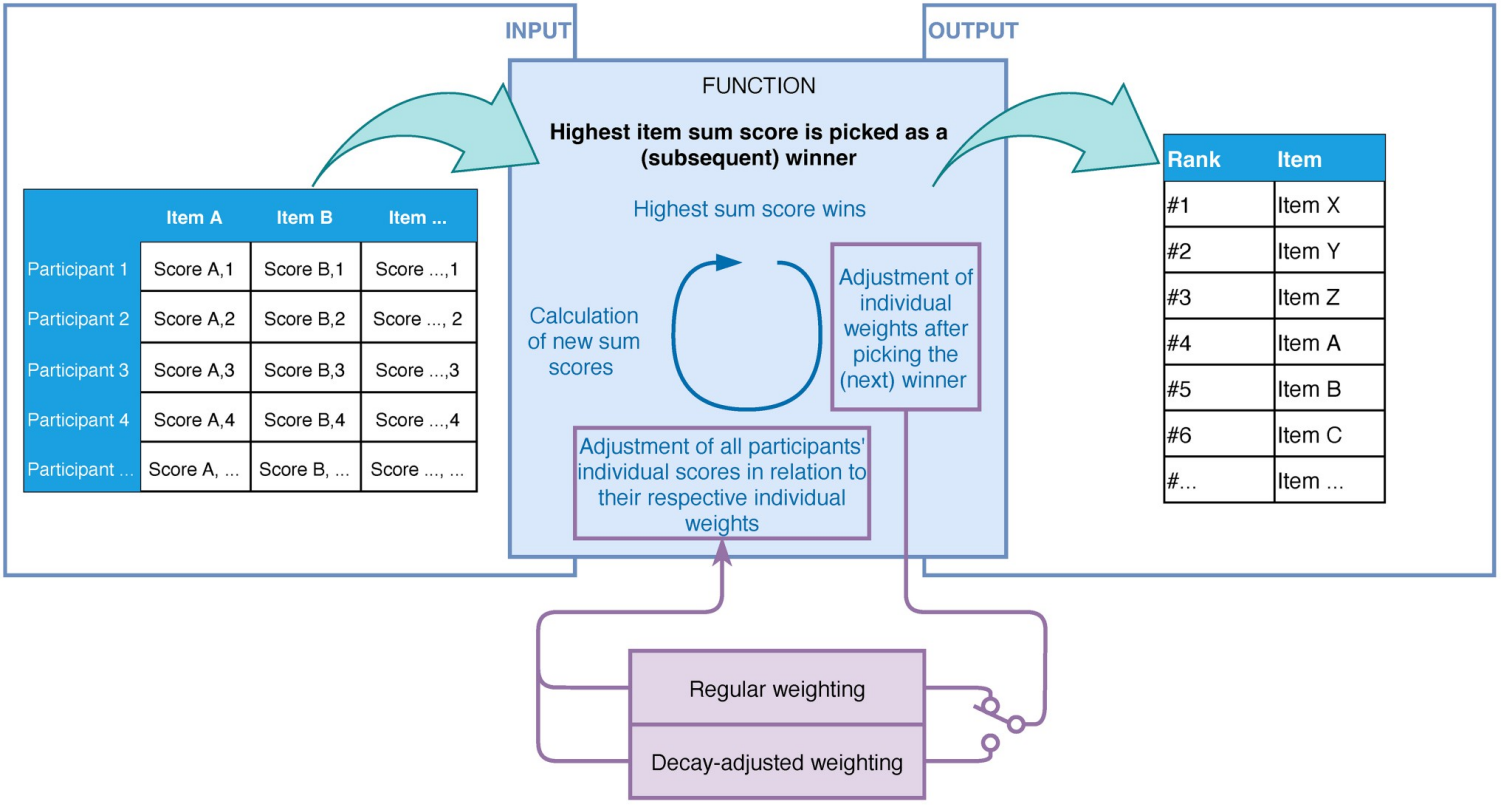
The REPS-tool as a function component. The figure shows the REPS-tool as a function component. The input section shows the input format for the tool. The function repetitively identifies the (new) highest total score as a winner and assigns subsequent ranks to winning items. Individual weights are adjusted after assigning a rank to a winning item. Scores are thereafter recalculated based on the individual weight. The tool has two weighting options: regular re-weighted range voting and decay-adjusted re-weighted range voting. New item sum scores are calculated and the highest total score is identified as the next winner. The tool’s output is a list of ranked items.

**Fig 4:**
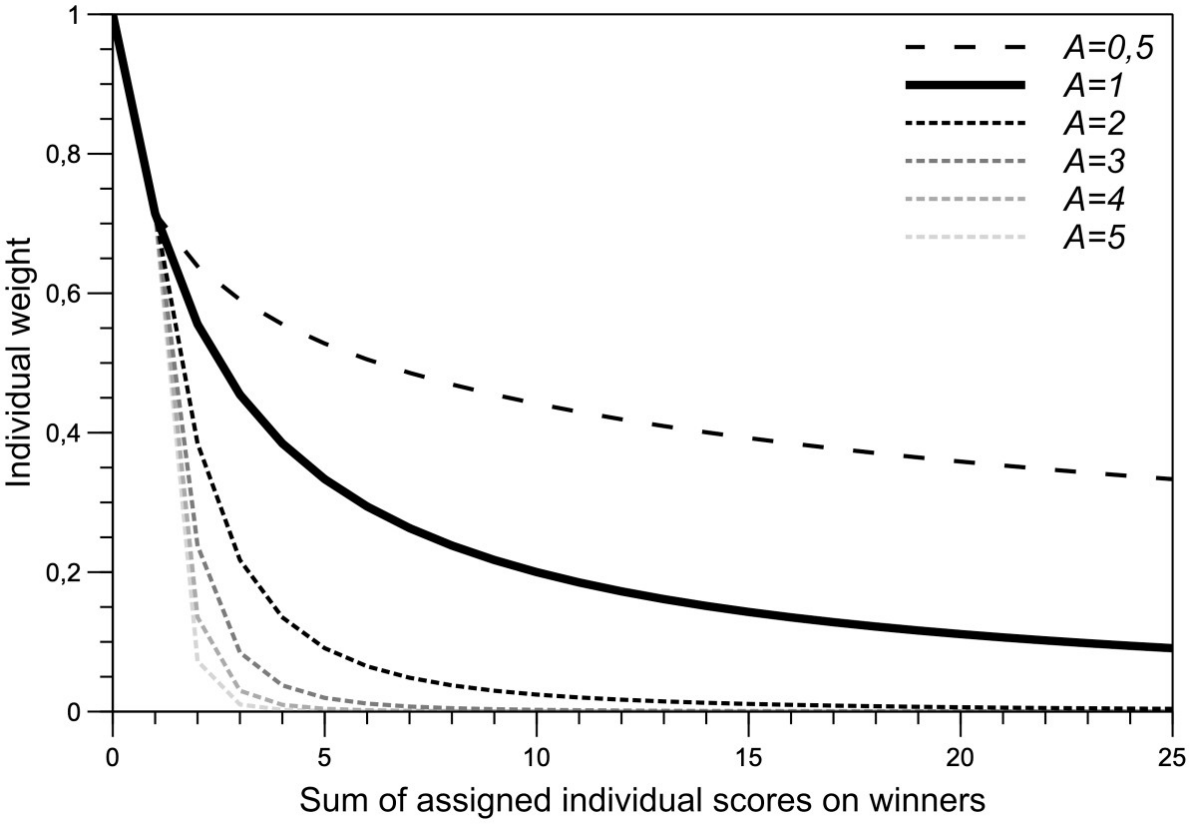
A graph showing the decay patterns of the individual weight. The decay-adjusted weighting method controls the decay pattern of the individual weights. Any number provided for A (i.e. decay aggression) larger than 1 results in a more aggressive initial decay of the individual weight when compared to the regular re-weighted range voting method (A = 1). Using A = 0.5 will ease the decay of the individual weights in comparison to the regular re-weighted range voting method. Weights in the graph were calculated using constant of 0.5 and a maximum scale score of 10.

The REPS-tool can be found in S4 File, programmed as an Excel-file. It was designed as a function component in a priority-setting assessment (Fig 3). Its input format is a matrix containing priority scores per participant per item, where rows represent participants (or subgroups) and columns represent items. The tool can highlight input outside the allowed range to validate the input and is capable of processing 100 items for 100 participants. A (subsequent) winner is automatically identified to which a rank can be semi-automatically assigned. To tool also identifies when and how many items are tied for a rank. Heterogeneity analyses within the REPS-tool could be decisive for which of the items to assign the tied rank to. Either the regular re-weighted range voting (Formula 1) or the decay-adjusted method (Formula 2) can be selected in the tool. In the latter, the decay aggression (*A*) is controlled in the tool’s parameter section. The decay adjusted method places additional emphasis on the participant’s first few assigned scores to the ranked winners. Fig 4 shows the pattern of the decay-adjusted individual weight depending on the value of *A*. Items with an assigned rank are ordered in a list by their rank and this output can be collected from the REPS-tool. More background information and guidance about how to use the REPS-tool can be found in the quick-start guide (S5 File).

### Real-word priority-setting

The priority-setting assessment in the Kleefstra syndrome CPG is displayed in an online supplementary file (S6 Fig 1 in S6 File), together with the assigned scores in an anonymized file (S7 File). Thirty-one panel members (including 10 patient representatives) assigned priority scores in the survey. The priority-setting assessment led to the final selection of 12 key questions by discussing the REPS-tool’s top-18 output (S6 Fig 2 in S6 File). Discussion resulted in merging four questions with other questions in the top-18 due to being similar topics. Three questions, ranked 13^th^, 15^th^, and 17^th^ in the top-18 output, were not selected. One question, initially not ranked in the top-18, was added through discussion. All 17 panel members, thus excluding the core group, voted in the poll regarding the priority-setting outcome. Eight members were ‘very content’ and nine were ‘content’ with the final priority-setting outcome as selected by the core group.

Four top-10 outputs were generated under different conditions (S6 Fig 3 in S6 File). Six of the same key questions remined in the tool’s output regardless of the condition, albeit different ranks were assigned to these questions in each respective condition. Seventeen panel members besides the core group participated in the online meeting where both the four top-10 outputs and the priority-setting outcome were evaluated. Sixteen panel members indicated which of the four top-10 outputs they preferred. Six panel members preferred the output generated from using scores of individual participants with a more aggressive decay (Option 4 from S6 Fig 3 in S6 File). Five panel members preferred the output obtained from the mean scores of sub-groups with regular re-weighted range voting (Option 1 from S6 Fig 3 in S6 File). Three panel members preferred the top-10 resulting from using the scores of individual participants with regular re-weighted range voting (Option 2 from S6 Fig 3 in S6 File). Using the scores of individual participants and a mild decay was preferred by 2 panel members (Option 3 from S6 Fig 3 in S6 File). The two most preferred options are compared in an online supplementary file (S6 Fig 4 in S6 File).

## Discussion

We developed a tool based on the re-weighted range voting mechanism which serves as a function component for CPG priority-setting assessments. While this function does not aim to achieve consensus, it can help narrowing down to a set of priority items by providing a ranked output. Additional process components within the priority-setting assessment can contribute to achieving consensus (e.g. discussing the tool’s output with the panel to determine a final outcome). The tool underwent user testing during its development phase. Based on the findings from testing and interviews, adjustments were made to increase the usability and a quick-start guide was developed. Thereafter, a real-world priority-setting assessment was performed to prioritize CPG questions using the REPS-tool as a function component. GPG panel members were (very) content with the final selection of 12 key questions resulting from the priority-setting assessment. Parallel to the real-world priority-setting assessment, we had the opportunity to examine various input formats and weighting methods to assess four hypothetical top-10 outputs generated by the REPS-tool. Interestingly, among the sixteen voting panel members only three preferred the use of individual scores with regular re-weighted range voting, which provided proportional representation. The most favored options were using mean sub-group scores as input with the regular re-weighted range voting weighting method, or using the individual scores with an aggressive decay (i.e. *A* = 4). These preferences warrant further examination and theoretical analysis of these conditions.

Using the mean scores of subgroups with regular re-weighted range voting, the proportional representation can be achieved at the subgroup level. This approach determines the ranked winners based on the priority assigned by subgroups, regardless of their size. This may be why panel members in the Kleefstra CPG identified this method as one of the two preferred options. However, it is not always feasible to predefine or differentiate subgroups. In such cases, obtaining mean scores per subgroup becomes impractical and individual scores are used as input instead. As an alternative option, an aggressive decay can be applied to quickly decrease the individual weights of participants. Those participants who had assigned (high) scores to the first few winners have their influence quickly reduced for identifying the subsequent winners. This could leave some room for smaller subgroups or individuals with high individual weights to have their priority items receive a higher ranking. However, there is a risk that a large group of participants with moderate or low individual weights might still outweigh a small group with high individual weights. Thus, theoretically, a more aggressive decay is needed to significantly reduce the influence of the participants initially ‘having their way’ with the first few winners and accommodate for smaller subgroups and individuals. This might clarify why an aggressive decay adjusted weighting method was the second of the two preferred options in the Kleefstra CPG priority-setting. It is therefore important to think about how the group of participants is composed and whether potential subgroup formation or unbalanced representation is considered (un)desirable. Subgroup formation or unbalanced representation might be desirable in some situations. For example, when the priority-setting assessment takes place in a more mono-disciplinary setting. A larger number of persons from one or a few selected disciplines will probably participate, because they will be affected most by the recommendations. One can argue that they then would need to have the most influence in the priority-setting assessment too. Theoretically, the regular re-weighted range voting mechanism could be used when subgroup formation or unbalanced representation is not considered problematic because of the mechanism’s proportional representation characteristic. However, subgroup formation or unbalanced representation might be undesirable in other situations. For example, when situations arise where the number of representatives within a subgroup might undesirably distort the outcome of the priority-setting assessment, leaving the important perspectives of small subgroups unexposed in the outcome. One could argue that smaller groups have important perspectives on priority too, but these are potentially underexposed due to a low number of representatives participating in the priority-setting assessment. For example, the patient representation during a priority-setting assessment for clinical practice guidelines can be smaller than the representation of clinicians, but they do have important perspectives on where their priorities lie. Such situations could also arise from practical limitations, as some disciplines may struggle to deliver representatives for all of the ongoing guideline developments and updates, let alone enough representatives to provide some counterweight in priority-setting assessments against large, well-represented disciplines. It seems that the decay-adjusted weighting method could be used to enhance less represented perspectives, although it remains challenging to determine which exact decay aggression should be used.

The REPS-tool uses a single priority score per participant per item. However, priority-setting assessments or tools described in literature seem to use multiple indicators [6, 7, 11, 13, 14]. When desirable, multiple scores could be averaged to comply to the input format requirement for the REPS-tool. A single overall score, however, might be sufficient for priority-setting in general [6, 7], although scores on individual indicators may help to resolve ties [6]. This could also be helpful for resolving ties in the REPS-tool. Furthermore, some basic heterogeneity analyses are available in the REPS-tool that can also assist in making such decisions. Another helpful tool for CPG question priority-setting is the UpPriority Tool, which contains six priority indicators [11, 13]. It furthermore contains guidance for applying the tool, rating the indicators, calculating scores, ranking scores, the priority decision, and reporting the outcome [11]. The REPS-tool does not contain any priority indicators or process components. Instead, users need to select their own process components (including the priority indicators if desirable) for their priority-setting assessment. This feature can be important in order to create or adapt assessments to the context of the priority-setting assessment when utilizing the REPS-tool.

Organizations performing priority-setting assessments may have different needs and operate within different contexts. Priority-setting assessments for CPGs in the context of venous thromboembolism [6] and COVID-19 dental care [7] showed differences in which indicators were significant predictors although using identical priority indicators. This could suggest that priority indicators might be dependent on the context [7], for example, the healthcare system an organization operates in. Using ‘the impact on the access to care’ as a priority indicator [11, 13, 20] may be less discriminating for organizations operating in socialistic healthcare systems, which allow a high degree of access by default. Also, the aim of priority-setting can be different. Existing CPG recommendations or sections can be prioritized for uptake in other CPGs [8], for implementation [9] or for updating [12]. All of such aims might require a different set of relevant priority indicators. Interestingly, literature shows that authors do select priority indicators based on the context, for example, of their national clinical practice [14]. Our frame of reference may aid in how priority-setting assessments are structured, while the steps, phases, and priority indicators described in literature [1] could guide decisions for process components regarding the organization’s needs and context within this structure. If an organization would consider implementing the REPS-tool, it would need to recognize that the tool is solely a function component (see Fig 3) in a priority-setting assessment (see Fig 2) and not a priority-setting assessment in itself. Organizations may self-tailor their priority-setting assessment as long as the process components in the priority-setting assessment lead to input that complies to the REPS-tool’s input format and when the organization desires a ranked list of items as output. Adaptations to the weighting methods can be made as all formulas and scripts are accessible and editable in the Excel-file when the current methods are not desired.

The theoretical nature of the REPS-tool as a function component is one of the limitations of the current study. Although we performed usability tests and piloted the tool in a real priority-setting assessment, there is still a need for a stronger evidence base regarding its usability. This includes determining which input format, weighting method, and decay aggression to use based on different contexts. We encourage others to use, test, compare, or adapt the REPS-tool and share their experiences. This collaborative effort will contribute to expanding the evidence base and help determine if the REPS-tool should be used in different contexts. We furthermore refined the tool based on the qualitative findings of the think aloud usability tests and semi-structured interviews. We did not perform a final check with the methodologists whether the adjustments addressed their usability matters and assumed the refinements were appropriate. Future plans involve a comprehensive evaluation of the tool’s usability, applicability, and viability in real-world assessments. However, at face value, most matters may seem to be addressable by providing background information about the tool (which the methodologists deliberately did not receive for the usability testing at the time) and by further automating the tool.

Future development of the REPS-tool could include development of the tool in software or apps linked to CPG databases for priority-setting in an online portal, including options to select different priority indicators and to elicit priority scores from stakeholders. Our pilot test focused on the satisfaction with the final outcome of a priority-setting assessment using the REPS-tool and four different outputs. Thorough evaluations of priority-setting assessment using the REPS-tool could be performed according to a pre-defined framework in the future [21]. Furthermore, an adapted version of the REPS-tool (not published) is currently being tested in-house at the Knowledge Institute of the Dutch Association of Medical Specialists with their stakeholders to assign priorities of CPG sections for updating as part of a continuous maintenance strategy. The use of the REPS-tool is also being considered in a second ERN-ITHACA CPG to select key questions for development after being well-received by the Kleefstra CPG panel. Furthermore, the tool could also be tested used outside the context of CPG development to prioritize any other item (e.g. knowledge gaps, patient reported outcome measures, systematic review questions, etc.). Hopefully, experiences with the REPS-tool and evaluations of priority-setting assessments using the tool will be shared.

## Conclusions

The REPS-tool has the theoretical capability to rank various items, such as key questions, recommendations, and CPG topics to support priority-setting during the development or updating of CPGs. It could also have applications for priority-setting outside the field of CPGs, such as research questions, patient reported outcome measures, knowledge gaps, and more. As a function component in priority-setting assessments, it allows for a wide range of choices within the priority-setting assessment as long as its input format requirement is met (i.e. a single score per item per participant). For example, different sets of priority indicators can be selected based on the specific context of the priority-setting assessment. While the REPS-tool assists in the ranking of items during a priority-setting assessment, it should be noted that it is not a consensus method in itself. When using the REPS-tool, it is important to consider the composition of the participants in the priority-setting assessment and determine whether there are (potential) imbalances and whether they are desirable or not. These considerations may affect decisions regarding the score input (e.g. individual or mean subgroup scores) and the choice of weighting method (i.e. regular or decay-adjusted). However, more empirical evidence may be needed to support these choices. The REPS-tool helps narrowing down a set of items, while additional steps in the priority-setting assessment may be used to obtain consensus for a final outcome. It’s worth noting that in the first real-world evaluation of priority-setting using the REPS-tool in CPG development, the panel members were (very) satisfied with the final outcome of the priority-setting assessment.

## Supporting information

S1 File Measurement protocol of the think aloud sessions and semi-structured interviews. This file shows the protocol for the tasks followed during the think aloud sessions and the questions asked during the semi-structured interviews.
(DOCX)

S2 File Published priority-setting assessments mapped over our frame of reference. This file shows priority-setting assessments mapped to process, function, and outcome components as described by our frame of reference.
(DOCX)

S3 File Qualitative analysis of the usability testing including the resulting action for improvement. This file shows quotes (Dutch) from the transcribed think aloud sessions and semi-structured interviews enriched with text describing the performed actions on-screen (in brackets). Quotes qualitatively received labels/observations (Dutch with English translation) and were clustered under a generalized interpretation of these labels/observations. Finally, actions for improving the tool’s usability were added for improvement of the tool.
(DOCX)

S4 File The REPS-tool programmed in microsoft excel. This file is the complete REPS-tool as a function, where input data can be entered in the function and the semi-automatic ranking within the tool results in output. Please refer to the quick-start guide for information about how to use the REPS-tool in Microsoft Excel.
(XLSM)

S5 File RE-Weighted priority-setting: A quick-start guide to the REPS-tool. This file is a guide to understand and use the REPS-tool in Microsoft Excel.
(PDF)

S6 File The priority-setting assessment and its outcomes in the Kleefstra guideline pilot. This file contains figures depicting the priority-setting assessment in the Kleefstra guideline pilot mapped to our frame of reference and its final outcome. Furthermore, the output generated by the REPS-tool under four conditions are shown and compared.
(DOCX)

S7 File The scores of all individuals participating in the Kleefstra priority-setting assessment. The anonymized file displays all scores the individual participants assigned to key questions in the priority-setting assessment of the Kleefstra guideline. This file is a real-world example that complies to the REPS-tool input format. The participants and their scores can be copy-pasted into the REPS-tool.
(XLSX)

## References

[1] El-Harakeh A, Lotfi T, Ahmad A, Morsi RZ, Fadlallah R, Bou-Karroum L, et al. The implementation of prioritization exercises in the development and update of health practice guidelines: A scoping review. PLoS One. 2020;15(3):e0229249. Epub 2020/03/21. doi: 10.1371/journal.pone.0229249 . 32196520 PMC7083273

[2] El-Harakeh A, Morsi RZ, Fadlallah R, Bou-Karroum L, Lotfi T, Akl EA. Prioritization approaches in the development of health practice guidelines: a systematic review. BMC Health Serv Res. 2019;19(1):692. Epub 2019/10/17. doi: 10.1186/s12913-019-4567-2 . 31615509 PMC6792189

[3] Martínez García L, Pardo-Hernandez H, Superchi C, Niño de Guzman E, Ballesteros M, Ibargoyen Roteta N, et al. Methodological systematic review identifies major limitations in prioritization processes for updating. J Clin Epidemiol. 2017;86:11–24. Epub 2017/05/28. doi: 10.1016/j.jclinepi.2017.05.008 . 28549931

[4] NIHR Global Research Health Unit on Global Surgery. Delphi prioritization and development of global surgery guidelines for the prevention of surgical-site infection. Br J Surg. 2020;107(8):970–7. Epub 2020/03/27. doi: 10.1002/bjs.11530 . 32212158 PMC7317442

[5] Ferguson M, Medley A, Rittenbach K, Brothers TD, Strike C, Ng J, et al. Priority setting for Canadian Take-Home Naloxone best practice guideline development: an adapted online Delphi method. Harm Reduct J. 2022;19(1):71. Epub 2022/07/03. doi: 10.1186/s12954-022-00650-4 . 35780136 PMC9250272

[6] Wiercioch W, Nieuwlaat R, Zhang Y, Alonso-Coello P, Dahm P, Iorio A, et al. New methods facilitated the process of prioritizing questions and health outcomes in guideline development. J Clin Epidemiol. 2022;143:91–104. Epub 2021/11/30. doi: 10.1016/j.jclinepi.2021.11.031 . 34843861

[7] Zaror C, Deana NF, Espinoza-Espinoza G, Aravena-Rivas Y, Muñoz-Millán P, Pineda P, et al. Questions and health outcomes prioritization for the development of a COVID-19 dental clinical practice guideline: A case study. J Eval Clin Pract. 2022;28(3):404–10. Epub 2022/01/27. doi: 10.1111/jep.13658 . 35080284

[8] NIHR Global Research Health Unit on Global Surgery. Global guidelines for emergency general surgery: systematic review and Delphi prioritization process. BJS Open. 2022;6(1). Epub 2022/02/25. doi: 10.1093/bjsopen/zrac005 . 35199142 PMC8867031

[9] Lynch EA, Lassig C, Turner T, Churilov L, Hill K, Shrubsole K. Prioritizing guideline recommendations for implementation: a systematic, consumer-inclusive process with a case study using the Australian Clinical Guidelines for Stroke Management. Health Res Policy Syst. 2021;19(1):85. Epub 2021/05/24. doi: 10.1186/s12961-021-00734-w . 34022906 PMC8140744

[10] Becker M, Neugebauer EA, Eikermann M. Partial updating of clinical practice guidelines often makes more sense than full updating: a systematic review on methods and the development of an updating procedure. J Clin Epidemiol. 2014;67(1):33–45. Epub 2013/10/16. doi: 10.1016/j.jclinepi.2013.06.021 . 24125894

[11] Sanabria AJ, Pardo-Hernandez H, Ballesteros M, Canelo-Aybar C, McFarlane E, Niño de Guzman E, et al. The UpPriority tool was developed to guide the prioritization of clinical guideline questions for updating. J Clin Epidemiol. 2020;126:80–92. Epub 2020/06/23. doi: 10.1016/j.jclinepi.2020.06.018 . 32565214

[12] Becker M, Jaschinski T, Eikermann M, Mathes T, Bühn S, Koppert W, et al. A systematic decision-making process on the need for updating clinical practice guidelines proved to be feasible in a pilot study. J Clin Epidemiol. 2018;96:101–9. Epub 2018/01/01. doi: 10.1016/j.jclinepi.2017.12.011 . 29289763

[13] Sanabria AJ, Alonso-Coello P, McFarlane E, Niño de Guzman E, Roqué M, Martínez García L. The UpPriority tool supported prioritization processes for updating clinical guideline questions. J Clin Epidemiol. 2021;139:149–59. Epub 2021/08/08. doi: 10.1016/j.jclinepi.2021.07.022 . 34363971

[14] Sereda M, McFerran D, Axon E, Baguley DM, Hall DA, Potgieter I, et al. A process for prioritising systematic reviews in tinnitus. Int J Audiol. 2020;59(8):640–6. Epub 2020/03/07. doi: 10.1080/14992027.2020.1733677 . 32134348

[15] Smith WD. Reweighted range voting–new multiwinner voting method. 2005.

[16] Central Committee on Research Involving Human Subjects. Non-WMO research [cited 2023 20th of October]. https://english.ccmo.nl/investigators/additional-requirements-for-certain-types-of-research/non-wmo-research.

[17] Central Committee on Research Involving Human Subjects. Your research: Is it subject to the WMO or not? [cited 2023 20th of October]. https://english.ccmo.nl/investigators/legal-framework-for-medical-scientific-research/your-research-is-it-subject-to-the-wmo-or-not.

[18] Kok J, Smith WD. Re-weigted Range Voting—a Proportional Representation voting method that deels like range voting [cited 2023 15th of May]. https://www.rangevoting.org/RRV.html.

[19] Eccles DW, Arsal G. The think aloud method: what is it and how do I use it? Qualitative Research in Sport, Exercise and Health. 2017;9(4):514–31.

[20] Agbassi C, Messersmith H, McNair S, Brouwers M. Priority-based initiative for updating existing evidence-based clinical practice guidelines: the results of two iterations. J Clin Epidemiol. 2014;67(12):1335–42. Epub 2014/09/14. doi: 10.1016/j.jclinepi.2014.06.013 . 25216900

[21] Sibbald SL, Singer PA, Upshur R, Martin DK. Priority setting: what constitutes success? A conceptual framework for successful priority setting. BMC Health Serv Res. 2009;9:43. Epub 2009/03/07. doi: 10.1186/1472-6963-9-43 . 19265518 PMC2655292

