# Supplementary material for: Introducing re-weighted range voting in clinical practice guideline prioritization: Development and testing of the re-weighted priority-setting (REPS) tool
Source: PLoS One. 2024 Apr 5;19(4):e0300619. doi: 10.1371/journal.pone.0300619 (PMC10997121; doi:10.1371/journal.pone.0300619)
Supplement: S1 File — This file shows the protocol for the tasks followed during the think aloud sessions and the questions asked during the semi-structured interviews. (DOCX) [file pone.0300619.s001.docx]

**Supporting information file 1 – Measurement protocol for the think aloud session and semi-structured interview**

**1. PREPARATIONS**

Email the participant the following documents prior to the session

- PRIORITIZATIONTOOL_EMPTY.XLSX
- SCENARIO_1.XLSX
- SCENARIO_2.XLSX
- FOCUS POINTS.DOCX
- AI_IMAGE3_MOERB.JPG

**2. ASK FOR CHARACTERISICS**

- Sex (m/f)
- Age (years)
- Overall working experience (years)
- Working experience in guideline development (years)
- Education (Msc./PhD)
- Experience with priority-setting (y/n)
- Have you seen the current priority-setting tool somewhere before this session? (y/n)

**3. INSTRUCTIONS (up to 5 minutes)**

[MAIL: FOCUS POINTS.DOCX]

This is a ‘think aloud’-session. Here, the purpose is to verbalize your thoughts while performing a task. The goal of the session is to research the usability and completeness of the priority-setting tool for future users. The tool is developed to allow for a ranked priority-setting. Outdated guideline modules are eligible for prioritization.

The session will consist of a warming-up based on a picture, a first introduction to the tool, the use of the tool in two scenarios, and finally a short semi-structured interview. During the two scenarios you will be receiving some specific tasks, however, don’t wait for these tasks to start verbalizing your thoughts.

Later on, I would like to ask you to share your screen so I can see how the priority-setting tool is being used. The session is being recorded to produce a transcription. The transcription will be qualitatively analyzed and all data will be anonymized in the data-analysis and research report. You can turn off your camera if you don’t want any video recordings of your persona being made.

There are some specific focus points in this think aloud session where I would like to ask some of your attention:

- Stay focused on your task.
- Try to verbalize your thoughts instead of describing your actual actions.
- You do not have to explain or substantiate your thoughts but keep verbalizing your thoughts.
- Ask any question out loud but the session leader will not answer any question.

Do you have any questions?

You are allowed to revisit these focus points during the session. Refer to the mail I had sent you (focus points.docx).

Are you ready to continue to the warming-up? I will then start the recording.

**4. WARM-UP SESSION (up to 5 minutes)**

[TURN ON RECORDING]

[SHARE SCREEN]

I will show you an image. Think of the focus points we just have discussed. Do you want me to repeat them before you start?

[SHOW IMAGE AI_IMAGE3_MOERB.JPG]


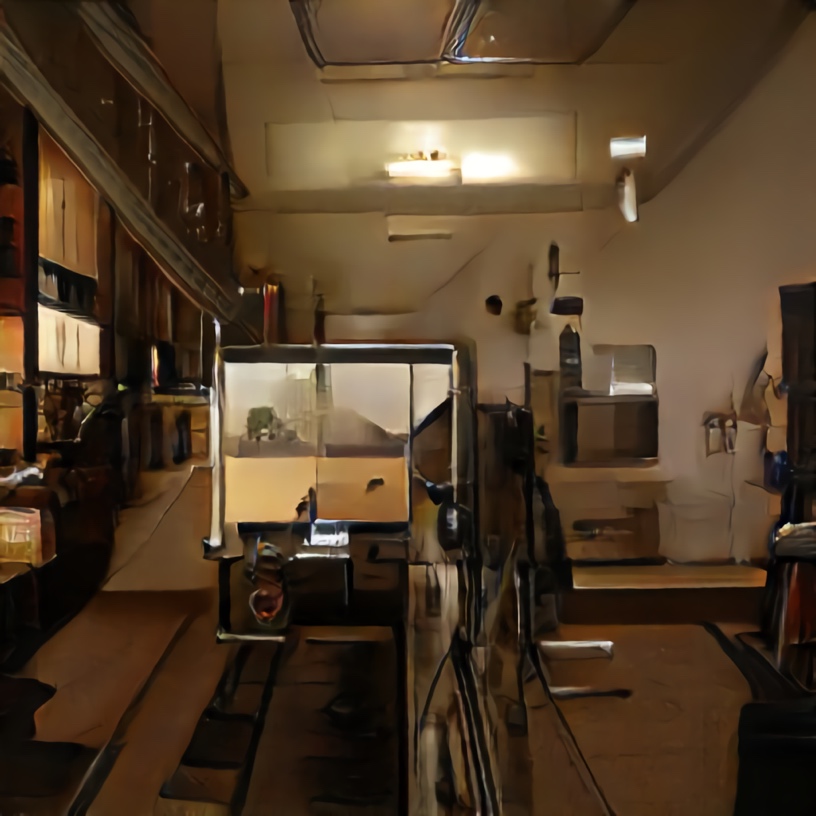


1. Analyze the image and use the think aloud-method to verbalize your thoughts.

[STOP SCREEN SHARE]

| *Note: The image was created using an artificial intelligence at Artbreeder.com under the creative commons CCO license.*  *The image is available through the following link: https://www.artbreeder.com/i?k=2c1196ef89bc996dfef0* |
| --- |

**5. FAMILIARIZATION WITH THE TOOL (up to 5 minutes)**

[MAIL: PRIORITYZATIONTOOL_EMPTY.XLSX]

Would you start to share your screen from this moment on?

[PARTICIPANT SHARES SCREEN]

Open the tool in Excel. The formulas underneath the spreadsheets are locked. Thus, you cannot break the tool.

1. Look at the tool and browse the tabs. Verbalize your thoughts.
2. Participants score from 0 to 10. Here, 0 = no priority, 1 = the lowest possible priority, and 10 = the highest possible priority. Randomly fill out some scores in the designated cells and see what the tool does.

**6. SCENARIO TESTS (up to 30 minutes)**

[MAIL: SCENARIO_1.XLSX]

[CHECK: MAX SCORE = 10, WEIGHT = 0.5, PENALTY METHOD = 0]

[CHECK: NO VALUES IN CELLS CONCERNING RANKING, SCORE, OR ORGANIZATION]

Scenario 1:

Open the Excel-file ‘Scenario_1.xlsx’. This is a file that contains priority scores assigned by participants.

1. Try to get the item names (see tab ‘modules list’) and scores from the scenario Excel-file into the priority-setting tool.
2. Try to rank the scored items by inserting their rank, as a number, above the winner.
3. Try some analyses concerning the central tendency and dispersion measures to discover some modules with a high variance in their scores.
4. See if you can get the ranked list with items from the tool into a Word-file for a hypothetical feedback moment with the working group.

| Note: Scenario 1 is a straightforward scenario with a tied ranking once (multiple winners). Scores were randomly assigned in Excel using the =RANDBETWEEN(0;10) function. Only two item scores were manually adjusted to set up a tied ranking for position two. |
| --- |

Scenario 2:

Open the Excel-file ‘SCENARIO_2.XLSX’. Participants voted along the interests of their association in this scenario. That is, modules of their ‘own’ association were assigned high priority scores and other modules were assigned no score or a low priority score. We can apply weights by activating a penalty method. The weight is adjustable in one of the penalty methods.

1. Try to get the item names (see tab ‘modules list’) and scores in the Excel-file into the priority-setting tool.
2. Try to rank the scored items in the priority-setting tool to create a top-15 by inserting their rank, as a number, above the winner.
3. See if you can get the ranked list with items from the tool into a Word-file for a hypothetical feedback moment with the working group.
4. Fill out the number of voters per association in the priority-setting tool. See the tabs ‘Voters’ in the priority-setting tool and the tab ‘Participation’ in SCENARIO_2.XLSX.
5. Clear your top-15 ranking in the row where you just had inserted the ranks. Choose a penalty method in the priority-setting tool and create a new top 15.
   1. [If the participant chooses method ‘2’: insert ‘4’ in the cell concerning aggression]
6. See if you can get the ranked list with items from the tool into a Word-file for a hypothetical feedback moment with the working group.

You can stop sharing your screen with me.

[STOP SHARE PARTICIPANT’S SCREEN]

| Note: Scenario 2 is a scenario where associations had different numbers of representatives to vote, and those delegates specifically voted along the interest of their ‘own’ association. Scores were manipulated to achieve this situation. For items of their own association the Excel-function =RANDBETWEEN(8;10) was used while =RANDBETWEEN(0;4) was used for all other modules. Some participating associations did not have ‘own’ items, the scores of their representatives were randomly assigned using =RANDBETWEEN(0;4). |
| --- |

**7. SEMI-STRUCTURED INTERVIEW (up to 15 minutes)**

We will now continue with a semi-structured interview. There are 10 questions where you can provide your opinion about the tool.

- What did you think of the ranked outcome in scenario 2 where delegates voted along the interests of their association?
- What do you think of the current features in the priority-setting tool?
- Which features do you miss when you want to use the priority-setting tool in a priority-setting meeting with a working group?
- What do you think of the overview and structure of the priority-setting tool?
- How could the overview and structure of the tool be further improved?
- What do you think of the ease of use of the priority-setting tool?
- In what areas can the ease of use be improved?
- What should you further know or be able to do before you can use this tool in a priority-setting meeting with a working group?
- What should a working group know or be able to do before this tool can be used in a priority-setting meeting with a working group?
- Do you have any thoughts about the priority-setting tool which have not been addressed until now?

**8. CLOSURE**

I will now stop the recording

[STOP RECORDING]

The session has ended. We will analyze your input and with that we hope to improve the priority-setting tool. Thank you very much for your participation!

[CLOSE VIDEO CONFERENCE CALL]

NB1: Two penalty methods were present in this preliminary version of the tool. In later versions we removed one of the methods, which also expired the need for using the tab ‘voters’ in the tool.

NB2: The remaining penalty-adjusted weighting method and penalty aggression parameter were later renamed to decay-adjusted weighting method and decay aggression, respectively.
