# Supplementary material for: Introducing re-weighted range voting in clinical practice guideline prioritization: Development and testing of the re-weighted priority-setting (REPS) tool
Source: PLoS One. 2024 Apr 5;19(4):e0300619. doi: 10.1371/journal.pone.0300619 (PMC10997121; doi:10.1371/journal.pone.0300619)
Supplement: S2 File — This file shows priority-setting assessments mapped to process, function, and outcome components as described by our frame of reference. (DOCX) [file pone.0300619.s002.docx]

**Supporting information file 2 – Mapping published priority-setting assessments to the frame of reference.**

***S2 Figure 1 – The priority-setting assessment described in Sanabria (2021) was mapped to the components in the frame of reference.***

Visualized by M.S. Oerbekke using the frame of reference. Assessment from: *Sanabria AJ, Alonso-Coello P, McFarlane E, Niño de Guzman E, Roqué M, Martínez García L; UpPriority Implementation Working Group. The UpPriority tool supported prioritization processes for updating clinical guideline questions. J Clin Epidemiol. 2021 Nov;139:149-159. doi: 10.1016/j.jclinepi.2021.07.022. Epub 2021 Aug 5. PMID: 34363971.*

**

***S2 Figure 2 – The priority-setting assessment described in Ferguson (2022) was mapped to the components in the frame of reference.***

Visualized by M.S. Oerbekke using the frame of reference. Assessment from: *Ferguson M, Medley A, Rittenbach K, Brothers TD, Strike C, Ng J, Leece P, Elton-Marshall T, Ali F, Lorenzetti DL, Buxton JA. Priority setting for Canadian Take-Home Naloxone best practice guideline development: an adapted online Delphi method. Harm Reduct J. 2022 Jul 2;19(1):71. doi: 10.1186/s12954-022-00650-4. PMID: 35780136; PMCID: PMC9250272.*

**

***S2 Figure 3 – The priority-setting assessment described in Wiercioch (2022) was mapped to the components in the frame of reference.***

Visualized by M.S. Oerbekke using the frame of reference. Assessment from: *Wiercioch W, Nieuwlaat R, Zhang Y, Alonso-Coello P, Dahm P, Iorio A, Manja V, Mustafa RA, Neumann I, Ortel TL, Rochwerg B, Santesso N, Vesely SK, Akl EA, Schünemann HJ. New methods facilitated the process of prioritizing questions and health outcomes in guideline development. J Clin Epidemiol. 2022 Mar;143:91-104. doi: 10.1016/j.jclinepi.2021.11.031. Epub 2021 Nov 26. PMID: 3484386*
