# Supplementary material for: Introducing re-weighted range voting in clinical practice guideline prioritization: Development and testing of the re-weighted priority-setting (REPS) tool
Source: PLoS One. 2024 Apr 5;19(4):e0300619. doi: 10.1371/journal.pone.0300619 (PMC10997121; doi:10.1371/journal.pone.0300619)
Supplement: S3 File — This file shows quotes (Dutch) from the transcribed think aloud sessions and semi-structured interviews enriched with text describing the performed actions on-screen (in brackets). Quotes qualitatively received labels/observations (Dutch with English translation) and were clustered under a generalized interpretation of these labels/observations. Finally, actions for improving the tool’s usability were added for improvement of the tool. (DOCX) [file pone.0300619.s003.docx]

**Supporting information file 3 – Qualitative analysis of the usability testing**

**S3 Table 1. Part 1: Familiarization**

| **P** | **Participant text** | **Label/observation** | **Interpretation** | **Actions to improve** |
| --- | --- | --- | --- | --- |
| 1 | Er staan heel veel items zo te zien. Het gaat door tot 100. Ehm, een aantal formules zie ik staan. Ik kan nergens op klikken [klikt willekeurig cellen aan op het tabblad RRV], ik weet niet of dat klopt… alleen de tabbladen. | 1^e^ indruk: veel items  [First impression: lots of items] | First impression: large and complex | Provide an introduction to the tool’s structure in an accompanying document |
| 1 | [klikt tabblad RRV aan] Ja, het eerste tabblad vind ik overweldigend [scrollt horizontaal], ziet er heel ingewikkeld uit. [scrollt verticaal] | 1^e^ indruk: overweldigend  [First impression: overwhelming] |  |  |
| 2 | Oke, ehmm. Op het eerste ook zie ik een best wel complex-ogend.. systeem. Ehm… Waar soort van optelsom van iets van lijkt te worden gemaakt. | 1^e^ indruk: complex ogend [First impression: looks complex] |  |  |
| 3 | Het eerste wat mij opvalt is dat hier allemaal ‘hash-tag getal uitroepteken’ staat. Dat zijn natuurlijk formules die er onder liggen. Tenminste dat eh, dat lijkt mij zo. Het ziet er wel een beetje daunting uit [scrolt naar boven en beneden], dat ik denk: oeh, daar moet ik iets invullen! Maar dat is vast niet zo. | 1^e^ indruk: afschrikwekkend  [First impression: daunting] |  |  |
| 4 | [[scrolt door tabblad ‘RRV’] Ja, nou, eerste wat ik opende dacht ik: wow wat is dit groot, ofzo. | 1^e^ indruk: groot  [First impression: large] |  |  |
| 2 | Nou de eerste feed is zeg m… Of de eerste tabblad is mij zeg maar niet per se duidelijk wat er nu moet komen te staan, behalve dat hier ‘winner’ [wijst met cursor naar rij 3 ‘Winner’] staat.  (…)  klikt op tabblad labels list, scrollt verticaal] Dit is gewoon allemaal leeg. Geen idee wat hier allemaal ingevuld moet worden, misschien de.. zo de individuele modules.  (…)  [klikt op tabblad verenigingen] Nou, hier nog een lijstje met de verenigingen… [scrollt verticaal] met de afkortingen er achter. Wat was dit dan? [klikt op tabblad labels list] Even kijken, hoor. [klikt op tabblad voters, scrollt verticaal] Oh daar kan je het waarschijnlijk, ’t is een soort van tabblad wat gewoon, [klikt op tabblad verenigingen] alleen de afkorting geeft. [klikt op tabblad RRV] Nog een keer naar het eerste tabblad. Dit is echt eindig, oneindig lang. Ik weet niet wat RRV ranking betekent en ik snap het ook niet.  (…)  [scrollt verticaal] Nou, ik denk dat dit uiteindelijk een soort van de overzichtspagina moet zijn waarop de scores straks komen te zien. Maar ik snap nog niet zo goed hoe dat precies moet gebeuren. | Structuur van de tool niet direct helder  [Structure of the tool is not immediately clear] | Structure may be unclear at first | Provide an introduction to the tool’s structure in an accompanying document |
| 4 | En dat ik nu even niet het idee heb wat moet ik nu hier mee doen. Ik heb echt geen idee wat de bedoeling hier bij is, als ik het zo zie.  (…)  En als ik vooral naar de eerste pagina [klikt op tabblad ‘rrv’], of het eerste tabblad, die lijkt mij het belangrijkste en dank ik… dan heb ik echt [scrolt naar beneden] even geen idee wat ik daar… in moet vullen.  (…)  Oh nee. Maar ik heb dus inderdaad geen idee hoe je dit in zou moeten vullen, nu zeg maar. | Structuur van de tool niet direct helder  [Structure of the tool is not immediately clear] |  |  |
| 3 | Ahja, dit is, dit is wel handig [wijst met cursor naar uitleg bij tool parameters]. Hier staan bepaalde scores, en ook al wat ingevuld met ook een uitleg erbij. Dat is prettig. | Ervaart de begeleidende teksten in de tool als prettig  [Experiences the guiding texts within the tool as helpful] | Helpful guiding texts | - |
| 3 | Ik krijg een klein beetje.. eh.. associaties met, eh, SPSS, waar je ook in verschillende tabbladen dan verschillende, eh, typen informatie kan invullen. | Vergelijking met andere software  [Comparing to other software] | Initial similarities | - |
| 3 | [klikt op tabblad ‘Ranking outcome’]  (…)  Dus dit is, dit is eigenlijk om het.. gebruikersgemak nog verder te vergroten. Namelijk, om een soort tool aan te bieden dat je de eh, de.. de ranking van de modules eh, gewoon ergens anders naartoe kan kopiëren. | Ziet hoe de manier van output is bedacht en ervaart dit als het vergroten van gebruiksgemak  [See show the output was designed and thinks this inreases usability] | Initial thoughts on tool output | - |

**S3 Table 2. Part 2: Adding scores**

| **P** | **Additional instruction** | **Participant text** | **Label/observation** | **Interpretation** | **Actions to improve** |
| --- | --- | --- | --- | --- | --- |
| 1 |  | [klikt op vergrendelde cellen] Maar waar kan ik dan klikken? Hier [selecteert cel waar scores ingevuld kunnen worden]? Bij scores?  (…)  [selecteert cel waar de rank kan worden aangegeven] Oh wacht, hier kan ik ook wat doen. En de bedoeling was dat ik scores ging invullen nu, he? [vult 0 in de cel in]  (…)  Van de deelnemers…. Maar ben ik dan zelf de deelnemer? [klikt tabblad Voters aan] Of moet ik hier… [klikt tabblad RRV aan] Nee… [selecteert cellen voor de rank en vult er willekeurig cijfers in] Ik neem aan dat ik hier dan wat moet invullen. Hoeveel wil je dat ik er doe? | Structuur niet helder: items, deelnemers, scores  [Unclear structure: placement of itemnames, participants, scores] | Unclear placement of item names, participants, and scores at first | Provide an introduction to the tool’s structure in an accompanying document |
| 2 |  | Ja. Even kijken, dan denk ik [klikt op tabblad ranking outcome, klikt op tabblad labelslist] dat we in.. Hmm.. Zal dat in deze moeten? Ik ga gewoon even proberen. [vult cijfers in de cellen die bestemd zijn voor de itemnamen in] Acht, Negen, drie, twee, vier. | Niet direct duidelijk waar de scores ingevuld moeten worden  [Unclear structure: placement of scores] |  |  |
| 2 |  | He?! Even kijken hoor. [selecteert de cel voor module 1 die bedoeld is voor de ranking] Oke, zeg dat ik een 10 geef aan de eerste module [vult 10 in de cel in]. Oke wacht, en een 7, [vult scores in voor modules 2 t/m6 in de cellen die bestemd zijn voor de rank] en een 8, en een 3, en een 2, en een 5. Hmm, even kijken wat er nu gebeurd. [scrollt verticaal] Ik zie eigenlijk nog… [kucht] Maximale score… [inaudible] Ehm, ik vraag me af of ik het wel goed doe, want… [klikt op tabblad voters] Of ik nou de opdracht wel begrepen heb? | Niet direct duidelijk waar de scores ingevuld moeten worden  [Unclear structure: placement of scores] |  |  |
| 4 |  | Dus als ik zeg: hier 2. [klikt de correcte cel aan voor de eerste persoon in de kolom voor namen] En hier dan even een naam noemen, [vult in: ‘module heup’] module heup. | Structuur: plaats scores en itemnamen niet direct helder  [Unclear structure: placement of scores and item names] |  |  |
| 4 |  | Maar als ik dan hier [klikt de cel voor organization aan voor de tweede persoon] nog eentje invul, bijvoorbeeld <*wetenschappelijke vereniging*> [kopieert en plakt de cel van de eerste persoon voor de tweede persoon], en dan hier weer module heup [kopieert ‘module heup’ in kolom naam van de eerste persoon naar de cel voor naam voor de tweede persoon] , wat gebeurt er dan? Krijg je dan twee mensen die…? Oh ja, twee mensen die het gescoord hebben. Totale… en het gemiddelde is dan 2. Ahh, oke. En als ik dan hier een andere module doe van de.. [selecteert cel voor de organisatie van de derde persoon, vult in ‘NOV ‘ en krijgt een melding dat deze organisatie niet voorkomt in de lijst] ho, nja, oh dan kan je natuurlijk een keuze maken [opent het drop-down menu] uit het tabblad die daar staat. Nou, als ik dan hier ook de <*wetenschappelijke vereniging*> doe… [selecteert <*wetenschappelijke vereniging*> uit het drop-down menu] en dan hier [selecteert de cel voor name van de derde persoon, vult in: ‘module knie’] ‘module knie’.. [selecteert de cel voor de score van persoon drie op item 1, vult 3 in] dan zou je hier score drie krijgen. Hey? Ik had gedacht dat je dan hier weer een nieuwe rij op rij G of wat is het?  (…)  modules moeten denk ik hier [klikt op tabblad ‘ranking outcome’]. Maar hier krijg ik dan niks [scrolt op en neer]. Je zou denken dat je dan hier een soort score uit krijgt [klikt op tabblad ‘verenigingen’, klikt op tabblad ‘ranking outcome’, klikt op tabblad ‘RRV’]. Maar ik snap ook überhaupt niet waarom je dan hier [wijst met cursor naar item namen in rij 1] nu pas de module knie ziet staan. En niet als ik hier [wijst met cursor naar de kolom voor namen van deelnemers] dan bij ‘naam’ module knie neer zet, dat het dan allemaal bij elkaar komt in deze rij. | Structuur: verwisseling van naam deelnemer met naam item in de tool  [Unclear structure: placement of participants and itemnames] |  |  |
| 1 | [I]: Hmm-Hmm. Zou je die rij eh eens voor mij helemaal leeg willen maken? Die rank?  [P01]: Die rank helemaal leeg?  [I]: Ja, ja wat je net hebt ingevuld? Oke, en bij de cellen bij scores… zie je ‘m staan? Daar, ja. Zou je daar eens getallen willen invullen? | [maakt de rij voor ranking leeg en selecteert een cel waar scores ingevuld kunnen worden] Hmm-Hmm. Even kijken, dan ga ik hier dingen invullen [vult voor item 1 meerdere willekeurige scores in]. Dan krijgen we een winner al te zien. [lacht]. Oh wacht, net heb ik de rank ingevuld en nu is.. pas de scores. Dan zat ik net denk ik verkeerd.  (…)  Maar nu staat er alleen bij item 1 wat, voor de rest nog niks. [vult één score in voor zowel item 2 als 3] Ah zie, dus dat doe je onder elk item. Oh dus nu heb ik voor verschillende organisaties wat ingevuld, eigenlijk voor verschillende mensen [selecteert lege cellen waar naam ingevuld kan worden]. Dus nu heb je meerdere voters voor 1, als ik het goed zie… | Structuur wordt helderder na een kleine aanwijzing: rank, score, deelnemers, items  [Structure becomes more clear after a small instruction: placement of rank, scores, participants, item names] | Placement of scores, participants, itemnames, and ranks becomes more clear after some instructions | Instruction and background information seem to be helpful to recognize the tool’s structure. Provide an introduction to the tool’s structure in an accompanying document |
| 2 | [I]: Oke, je mag de.. de rank wat je nu net hebt ingevuld in die rij…  [P02]: Deze? [selecteert de cel met de rank voor module 1 in rij 2]  [I]: Ja, die mag je even helemaal leeg maken.  [P02]: [verwijdert alle ranks in rij 2]  [I]: Oke, en onderin, als je ietjes naar beneden scrolt,  [P02]: [scrollt verticaal]  [I]: ..daar staan de scores.  [P02]: heb je het over maximum…? [wijst naar cellen m.b.t. max score en penalty] Waar precies moet ik kijken?  [I]: Nee, nog iets naar onder.  [P02]: [scrolt verticaal]  [I]: Daar heb je organisatie, name, scores…  [P02]: Ja.. [klikt de cel aan die bestemd is om scores in te vullen voor de eerste persoon en eerste module]  [I]: En bij scores mag je wat invullen als je wil.  [P02]: Ohke. [vult 8, 9, 1, 2, 4 in voor 5 verschillende personen voor module 1] Zoiets?  [I]: Ja. | Ja? [scrollt verticaal] Hey… Hmm? Oke, wacht even. Nu ineens is zeg maar de eerste module winnaar geworden omdat ‘ie 24 punten heeft. [scrollt verticaal] Ik snap alleen nu niet hoe dit nou kan, dat er… [scrollt verticaal] Waarom ‘ie dat nou alleen op de eerste module.. Ohh, wacht, omdat ‘ie in de kolom van 1 zit waarschijnlijk. Als ik het hier doe… [vult cijfers in voor 5 verschillende personen voor module 2] vijf, vijf, vier, drie, twee. [scrollt verticaal] Kijk, dan krijgt ‘ie ook een score. [scrollt verticaal] Ahh, oke. Ohh, oke. | Structuur voor scores invullen wordt duidelijker na een aanwijzing  [Structure becomes more clear after a small instruction: placement of scores] |  |  |
| 2 |  | Ah wacht, oke. [klikt op tabblad labels list] Dus dit is waarschijnlijk alleen maar een lijst waarin je… hier [selecteert de cel voor de eerste modulenaam] moet je waarschijnlijk je module zo neerzetten. [voert de tekst ‘module 1’ in de cel in, klikt op tabblad RRV]] En dan, ja.. Dit is… oke. Dus dit is alleen de titel. | Structuur: link tussen tab labels list en item namen in RRV tab is duidelijk  [Structure: link between labels list tab and RRV tab is clear] | Placement of item names, participants, organizations, and/or scores may be clear to some without prior instructions | - |
| 3 |  | Even kijken [scrollt naar beneden]. Ohja, hier kan ik dus de organisatie invullen [opent drop-down menu met alle organisaties] met een drop-down menu [scrollt door het drop-down menu]. Bijvoorbeeld de <*wetenschappelijke vereniging 1*> [klikt <*wetenschappelijke vereniging 1*> aan in het drop-down menu]. [klikt op cel D35 in de kolom ‘name’] En dit is dan de naam van degene die de score maakt, dus dat is, eh, dat ben ikzelf [vult haar eigen naam in]. En.. [klikt op het drop-down menu voor organisatie op rij 36] De <*wetenschappelijke vereniging 2*> [selecteert<*wetenschappelijke vereniging 2*> uit het drop-down menu]. Eh, noem eens wat. <naam persoon> [vult de naam van deze persoon in op de correcte plaats]. [selecteert NVDV in het drop-down menu in rij 37, vult een naam in in rij 37 op de correcte plaats] | Ziet zelf direct de structuur waar deelnemers en organisaties moeten worden ingevuld  [Immediately sees the structure: placement of participants and organizations] |  |  |
| 3 |  | [klikt op tabblad ‘Labels list’] Dat was deze. Even kijken [vult namen van modules in op de juiste plaats]. Ik doe het gewoon even makkelijk, want [inaudible]. Drie, vier, vijf. | Ziet zelf de structuur waar de itemnamen moeten worden geplaatst  [Sees the structure: placement of item names] |  |  |
| 3 |  | Nou, ik vind: drie, vier, een, acht, negen [vult deze score in op rij 35 op de juiste plaats]. De laatste vind ik dan heel belangrijk. <naam> vindt dan juist eh, deze en deze heel belangrijk [vult scores in op rij 36 op de juiste plaats]. Die medium, deze dan weer laag. En <naam> vindt eigenlijk alleen maar deze heel belangrijk [vult scores in op rij 37 op de juiste plaats]. Tien zelfs. | Ziet zelf de structuur waar de scores moeten worden geplaatst  [Sees the structure: placement of scores] |  |  |
| 3 |  | Oh kijk, nou komt er meteen al een ‘winner’ uit. Module 4. [scrolt naar beneden] Het lijkt er op, ja er zijn natuurlijk twee mensen die hem hebben geprioriteerd dus dat is ook wel logisch. | Ziet zelf direct dat de tool automatisch een winnaar identificeert  [Immediately sees that the tool automatically identifies a winner] | Automatic winner identification can be observed without prior instructions | - |
| 3 |  | Want [klikt op tabblad ‘ranking outcome’], staat dat dan meteen hier al in? Nee, nog niet [klikt op willekeurige cellen onder de kolommen ‘name’ en ‘rank’, klikt op tabblad ‘RRV’, klikt op tabblad ‘ranking outcome’]. Nou misschien heb ik het dan toch anders geïnterpreteerd dan ik dacht. Ik kijk in de ranking outcome en dan staat er nog niet iets | Manier van ranken nog onduidelijk: rol toekennen van ranks i.r.t. de ranking uitkomst nog onduidelijk  [Way of ranking still unclear: role of assigning ranks related tot he ranking outcome is still unclear] | Unclear role of assigning ranks | Provide information in an accompanying document about how to assign ranks and its role in relation tot he ‘ranking outcome’ tab. |
| 3 |  | Ik vraag mij af.. Ehm, moet ik dan hier ook nog number of voters invoegen [wijst met cursor cel aan bij number of voter bij <*wetenschappelijke vereniging*>] , aangezien we hier [klikt tabblad ‘RRV’ aan] ook de naam en organisatie invullen? Zal die dan dan ook niet automatisch kunnen… [klikt tabblad ‘Voters’ aan] eh… kunnen doorlinken naar deze tab [klikt tabblad RRV aan]? | Wens om invullen van aantal voters te automatiseren  [Wish to automate filling out the number of voters] | No automation for number of voters | We have decided that we will use a different weighting method independent of the number of voters per organization. Therefore, the number of voters per organization does not have to be filled out in the next iteration of the tool. |
| 1 |  | Even testen of dat nu ook zo is als ik meer invul [vult meerdere scores in voor items 2, 3, 4]. Even kijken. Wordt gewoon random… Oh 52 kan niet, ik krijg geen foutmelding als ik 52 invul. | Check voor (non) valide scores in tool  [Check for (non)valid scores in the tool] | Missing check for valid scores | We will add a button that checks for input larger than the maximum scale score and less than 0 in the score matrix. |

**S3 Table 3. Part 3: Using the tool in scenario 1**

| **P** | **Additional instruction** | **Participant text** | **Label/observation** | **Interpretation** | **Actions to improve** |
| --- | --- | --- | --- | --- | --- |
| 1 |  | Maar dit [selecteert cellen met modulenamen in de header op tabblad database in scenario 1] is makkelijker kopiëren als het in de itemlijst moet, toch? Of hoe zag dat ding er uit? [klikt op de cellen met itemnamen in het tabblad RRV] Kan hier niks in knippen, plakken… [klikt op tabblad voters, klikt op tabblad labels list] Dat moest natuurlijk hier. Ja. Oke… Dan is deze [klikt op modulenamen in tabblad modulelijst in scenario1] inderdaad makkelijk. | Input: plaatsing itemnamen eenvoudig  [Input: easy placement of itemnames] | Getting data into the tool is generally easy | - |
| 2 |  | Even kijken, dan ga ik naar eh [klikt op tabblad labelslist] die, denk ik. Label list. [plakt modulenamen in de correcte cellen]  (…)  Ik kopieer er nog een van de richtlijn duizeligheid bij ouderen. [plakt modulenamen in de correcte cellen]  (…)  Oke, ik heb nu in [selecteert alle cellen met module namen] totaal 19 labels gekopieerd, [klikt op tabblad RRV] die staan nu in mijn overzichtspagina ook netjes bovenaan. | Input in tool: eenvoudige input van item namen in de tool  [Easy input of item names in the tool] |  |  |
| 2 |  | Eehm, even kijken, ik heb hier eerst de vereniging. Die zet ik bij organizations. [plakt de verenigingen in de juiste cellen]  (…)  [plakt deelnemersnamen in de correcte cellen] De namen van de mensen er bij. En dan heb ik…. Kijk dit gaat gesmeerd. Hatsjee. [plakt de scores van de deelnemers in de correcte cellen] Ik plak het er in, en als het goed is.. [scrollt verticaal] Ha, kijk! Lichamelijk onderzoek bij duizeligheid is onze winnaar. | Input in tool: eenvoudige input van organisaties, deelnemers en scores in de tool  [Easy input of organizations, participants, and scores in the tool] |  |  |
| 3 |  | Ehm, dan kopieer ik eh.. de namen van de modules van de eerste richtlijn. Die zet ik dan in de tool… [klikt op tabblad ‘labels list’] bij labels list [selecteert 5 cellen, plakt module titels in de 5 correcte cellen]. Ik doe dan eigenlijk niks met… de namen van de richtlijnen [klikt op tabblad ‘RRV]. Volgens mij is dat ook.. niet zo’n probleem, want die kan ik ook niet kwijt verder. Eh, en het is ook niet zo relevant natuurlijk omdat we richtlijnoverstijgend gaan denken. Dus, de modules kopieer ik. Ik ga met het volgende rijtje modules door. Eh.. bij labels list [klikt op tabblad ‘labels list’], zo, [plakt volgende set moduletitels aansluitend in tabblad ‘labels list’] copy-paste. En de laatste richtlijn. Hier. Zo [plakt volgende set moduletitels aansluitend in tabblad ‘labels list’]. [klikt op tabblad ‘RRV’]Even kijken hoe dat er uit ziet. Inderdaad, ik heb nu… de modules [scrolt horizontaal heen en weer], de naam van de modules boven in staan. | Input: manier van itemnaam input naar de tool krijgen gaat eenvoudig  [Manner of item name input to the tool is easy] |  |  |
| 3 |  | Met de namen er in te zetten.[scrolt naar beneden, plakt de rij namen in de juiste kolom] En de organisaties die daar bij horen [plakt de organisaties in de juiste kolom]. Yes, dat pakt ‘ie ook. | Input: manier van deelnemers en organisaties input naar de tool krijgen gaat eenvoudig  [Manner of organization input to the tool is easy] |  |  |
| 3 |  | Oke, dus dan hebben we de scores nog, die zal ik dan ook even kopiëren. Zo. En dan… zet ik die ook [plakt de matrix van scores op de juiste plaats] hierin. | Input: manier van score input naar de tool krijgen gaat eenvoudig  [Manner of score input to the tool is easy] |  |  |
| 4 |  | [gaat naar de tool, klikt op tabblad ‘labels list’] In deze. [selecteert en kopieert de moduletitels van de eerste richtlijn in het databestand en plakt deze in de tool] | Input: manier van itemnamen input naar de tool krijgen gaat eenvoudig  [Easy way of getting item names into the tool] |  |  |
| 4 |  | Ja, ik weet niet [plakt de namen op de juiste plaats in de tool] of ik het nu in een keer weer goed zou doen, dat ik dan zou, zeg maar, nu weet ik dat er dan hier wordt bedoeld dat je dan al die scores zo daar neer zet. [kopieert de scores uit de dataset] Maar ik weet niet of ik dat dan de volgende keer… [plakt de scores op de juiste plaats in de tool] gebaseerd op wat hier dan nu alleen staat ook weer zou doen. Dan zou ik toch hier [wijst met de cursor naar rij 34 boven waar scores ingevuld worden] misschien ook wat neerzetten ofzo. Of dit [klikt op de tekst ‘scores’ in rij 34] iets aanpassen, waardoor dat dan duidelijker is dat je hier dus los de scores weergeeft. | Plaatsing correct, maar wens meer sturing over de plaats van de input van scores in de tool  [Place of score input in the tool is correct, but wishes for more guidance in the tool] |  |  |
| 4 |  | Maar de richtlijn weet ik niet of je die er dan ook bij moet benoemen. Maar ik zou zeggen, zelf zeggen, dat je dit [selecteert en kopieert de moduletitels van de tweede richtlijn in het databestand] hier in zet [plakt de module titels aansluitend in de tool]. Lijkt mij wel handig dat je dan ergens zometeen ook kan zien… [selecteert en kopieert de moduletitels van de laatste richtlijn in het databestand, plakt de moduletitels aansluitend in de tool] wat dan de richtlijn is.  (…)  Want nu zie ik al die namen hier wel staan, maar ik zou ook willen [klikt op tabblad ‘labels list’, klikt op tabblad ‘ranking outcome’] dat je dan kan zien… [klikt op tabblad ‘labels list’] uit welke richtlijn het komt.  (…)  Stel je hebt toevallig twee richtlijnmodules met precies dezelfde naam en je gaat ‘m prioriteren en.. je komt eruit, dan weet je niet meer uit welke richtlijn het komt. Ho. [klikt op Word ipv Excel] Dus dan zou ik toch zeggen, omdat ik nu niet zo heel goed weet hoe het werkt, om dan toch hier achter even die naam te kopiëren [kopieert de richtlijn naam van de eerste richtlijn in de dataset en plakt deze achter de module titel van het eerste item]. | Brontitel wordt meegenomen als item in de tool  [Title of the item’s source is used as an item in the tool] | There is a wish or consideration to identify the source of the items in the tool | We will not adjust the tool, but rather add guidance about source identification in a document accompanying the tool. |
| 1 |  | Moet de richtlijn er zelf ook bij? Ik denk het niet. [kopiëert en plakt modulenamen in de cellen in tabblad labels list] | Identificatie van de bron van items  [Identification of the source of the items] |  |  |
| 1 |  | Dan moet ik denk ik toch hier zijn. Dan verenigingen [kopieert en plakt verenigingen van scenario1 naar de correcte cellen in de tool ], dan namen…[kopieert en plakt de namen van scenario1 naar de correct cellen in de tool] en dan de scores per.. ding [selecteert alle scores in scenario1]. | Leereffect na aanwijzing in ‘scores invullen’ (vorige opdracht)  [Learning effect after instructions in previous assignment] | When the structure of the tool was unclear, participants were able to correctly place the tool’s input after a little instruction | Provide an introduction to the tool’s structure in an accompanying document |
| 1 | [I]: Net.. Net vulde je wat in op die eh.. ranking, die rij.  [P01]: Ja [klikt de eerste cell in de ranking rij aan]  [I]: Eh, als je nu boven de Winnaar eh.. 1 zet… Ja.. | [vult 1 in de cel boven winnaar in] Dan krijg je andere soort [inaudible], maar moet ik dan alsnog…? Oh wacht deze [selecteert de cel boven een MULTI(2)] is dan nu gelijk tweede. Krijgen ze dan allebei een 2? [vult 2 in boven de cellen van MULTI(2)] Oh, hij geeft nu zelf aan wat een… [vult ranking in voor 4 en 5] De winnaar is als je wat invult. [vult rank 6 in] Oke.. Ik heb nu 3 overgeslagen trouwens omdat ik twee keer 2 had, maar… maarja die staat er dan ook. Zes, Zeven [vult ranks in]. | Leereffect: Begrijpt de manier van ranken na kleine aanwijzing  [learning effect after a little instruction] |  |  |
| 4 | I]: oke, ik ga je wat dat betreft… Want dit punt eh, hier zat je net ook op en dus dat wordt wel helder uit de analyse. Ik ga je hier eventjes een klein beetje sturen. De naam, dat zijn de namen van de mensen die scoren. | Ohh. En de organisatie is dan… oke! [selecteert de rij met namen uit de dataset en kopieert deze] De naam van de mensen die scoren.[plakt de lijst met namen op de correcte locatie in de tool]  (…)  En dan de vereniging, die moet dan daarbij. [selecteert en kopieert de lijst met verenigingen in de dataset, plakt de lijst met verenigingen op de juiste plaats in de tool] En dan de scores die kan ik dan ook gewoon… [kopieert de scores voor het eerste item uit de database en plakt deze op de juiste plaats in de tool] Oh, wacht, denk ik, ofniet? Nu, hij komt natuurlijk gewoon onder de module denk ik, ahh! [kopieert de scores van item 2 in de dataset] Nu snap ik het denk ik, [ plakt de scores voor item 2 op de juiste plaats] want dan zet dit hier onder, jaa! Oh ik moest even een beetje, [lacht][kopieert de scores voor module 3 uit de dataset en plakt deze op de juiste plaats in de tool]. Nah, [kopieert de matrix van scores van de overige items uit de dataset] anders zou er misschien ook wel modules staan ofzo. | Herkent de structuur na een kleine aanwijzing en vult de input op de correcte plaats in de tool in.  [Recognizes the structure after a little instructin and fill out the tool’s input in the correct place] |  |  |
| 1 |  | Ik ga er even vanuit dat het op de juiste volgorde staat [plakt de scores in de juiste cellen in de tool]. Als het goed is.. klopt het dan nu [vergroot de tool naar full screen]. Ja, volgens mij is het nu compleet. | Check volgorde items en volgorde scores  [Checking the order of items and scores] | Participants seem to understand that the order of the scores must align to the order of the items. | Although it seems to be generally clear, it is an important aspect. We will elaborate about the input format in an accomanying document to the tool. |
| 3 |  | Even kijken, posterieur kanaal diagnose. Dat komt overeen met wat onder de richtlijn benigne paroxysmale positieduizeligheid valt. Ja, het zijn er heel veel, en de volgorde komt volgens mij ook overeen. | Check volgorde van lijst met itemnamen en scores of deze overeenkomen  [Checks the order of the item names and scores] |  |  |
| 3 |  | Ik zou eigenlijk moeten checken of alle modules, of de volgorde van alle modules helemnaal overeenkomt… met de volgorde van de modules.. die ik net in dat lijstje heb gezien. Nja, voor de snelheid ga ik er maar even vanuit dat het klopt. | Check volgorde van lijst met itemnamen en scores of deze overeenkomen  [Checks the order of the item names and scores] |  |  |
| 4 |  | Klopt het dat dat dan van, dat die andere modules niet gescoord zijn? Dus tot: ziekte geassocieerd. [controleert of alle items in rij 2 een score hebben in de kolom] Maar dat komt dan niet. Oh, dat komt bij mij natuurlijk niet overeen omdat ik richtlijn…, ahh, daarin heb genoemd. | Controle of scorematrix overeenkomt met de itemlijst; komt niet overeen omdat de brontitels zijn meegenomen als item  [Check is the score matrix align with the number of items; it doesn’t because source titles were used as items] |  |  |
| 1 |  | Opzich werkt dit wel handig zo, als er steeds een winnaar in beeld komt. [vult ranks in t/m 11] | Automatische identificatie van winnaar: positieve ervring  [Positive experience with the automatic identification of the winner] | Automatically showing the next winner in the tool is consedered healpful | - |
| 2 |  | Hebben we meerdere winnaars? [scrolt horizontaal] Nee. Oke, zit er in. | Controleert of de tool meerdere winnaars identificeert  [Checks if the tool identifies multiple winners] | Checking for multiple winners in the tool | - |
| 1 |  | Hoe ga ik dat dan weer doen..? Dat staat denk ik niet hier. [opent scenario1 ] Hm, hier staat het niet. [sluit scenario 1] Oke. Sum-scores… Winnaar… [klikt tabblad ranking outcome aan] Ik had verwacht dat je hier dan wat in zou zien [klikt tabbladen labelslist, ranking outcome, verenigingen, ranking outcome aan] , maar dat gebeurt dus niet automatisch. [klikt tabblad voters aan] Voters… [klikt tabbladen labels, RRV aan] | Manier van handmatig ranken niet direct duidelijk  [Method of manual ranking is not immediately clear] | It is generally not immediately clear where and how to rank in the tool without additional instructions | We will make the ranking more easy by automating the ranking process in the next iteration of the tool. This will ensure that it is no longer needed to manually assign rangs to winning items (except for ties) and that the user no longer needs to understand where and how to rank (except where to click a button for automated ranking and how to deal with ties). |
| 2 |  | [klikt op tabblad ranking outcome, klikt op tabblad RRV, klikt op tabblad ranking outcome] Kan je, [klikt op tabblad RRV] kan je nog één keer herhalen wat je vroeg? De, De… He? | Onduideljk waar er handmatig geranked moet worden  [Unclarity about where th manual ranking takes place] |  |  |
| 2 |  | Heb ik daar data voor gekregen? [scrollt verticaal] Even kijken... Dit zijn gewoon alleen maar gemiddelden. Hoe kan ik dit het gemakkelijkste doen? Ik ga eerst proberen te knippen en te plakken. [klikt op vergrendelde cellen] Even kijken, kan ik dat doen? Doet mijn Excel dat wel? Ik kan deze cel niet aantikken. [klikt op tabblad ranking outcome] Waarom werkt dat niet? Even kijken. [klikt op tabblad RRV] Misschien moet ik [klikt op vergrendelde cellen] dit [inaudible] doen. Wacht, kan ik die… [klikt op tabblad labelslist] hier in plakken? [selecteert en kopieert de modulenamen, klikt op tabblad ranking outcome, probeert de modulenamen te plakken in kolom B (gerankte modulenamen) te plakken maar krijgt een foutmelding] Nee, dat werkt niet. | Onduidelijk hoe er (handmatig) geranked moet worden  [Unclarity about how manual ranking takes place] |  |  |
| 3 |  | En dan heeft ‘ie al meteen al een winner aangewezen. [scrolt naar beneden] Ehm… Waar ik op zoek naar ben is ehm. D’r is nu steeds één winner [scrolt naar boven], maar je kan vast ook aangeven dat je een, een range wilt hebben van eh, weet ik veel, dat je de eerste vijf wilt meenemen [klikt op tabblad ‘voters’]. | Manier van handmatig ranken is nog onduidelijk  [Method of manual ranking is still unclear] |  |  |
| 4 |  | Ja, ik zou dan hier [klikt op tabblad ‘ranking outcome’] spieken, maar dan doet ‘ie dus niks. [klikt op tabblad ‘RRV’] Ehm, maar, ja ik… ik..  (…)  Ik ga gewoon even heel stom, ehh, tellen. [vult een 3 correct in] Maar nu schuift het op. Oh wacht, dat is dan drie. [vult 3 in op rij 2 bij een item die niet als winnaar werd geïdentificeerd] Ehh, dat is dan weer… [wijzigt de eerste 3 naar 4 in rij 2] Maar… Misschien. Vijf en zes [vult 5 en 6 niet correct in]. Ehm deze begint bij 68 en dan moet ik naar.. 59 [vult 7 in rij 2 niet bij winner in], 57 [vult 8 in rij 2 niet bij de winner in].. 56 [vult 9 in rij 2 niet bij de winner in], 54 [vult 10 in rij 2 niet bij de winner in]. Oh dan heb ik er twee met dezelfde dus dat wordt dan eh.. | Manier van handmatig ranken is onduidelijk  [Method of manual ranking is unclear] |  |  |
| 4 |  | Maar ik snap, ik heb dit nu gebaseerd op dit he? [wijst met cursor naar rij 4 met de ongewogen som scores] Van totaal op die totaal scores, alleen ik ben, ik sta er niet helemaal achter en ik snap niet wat er nu gebeurd. Ja ik, dat ‘ie dat nu gaat delen denk ik ofzo, maar ik snap niet waarom dit [wijst naar de cel met ‘winner’ in rij 3] dan gaat verschuiven zeg maar. Dat die winnaar dan hier naartoe gaat [wijs met de cursor naar de kolom waar ‘winner’ in rij 3 staat]. | Manier van handmatig ranken is onduidelijk  [Method of manual ranking is unclear] |  |  |
| 2 | [I]: Oke, ik zal ietsjes, ik zal ietsjes helpen in de.. in de richting.  [P02]: Ja,  [I]: Je ziet dat er een winnaar gekozen is,  [P02]: Ja.  [I]: En je kan boven die cel van de winnaar…  [P02]: [dubbelklik op de cel boven de winnaar]  [I]: Die cel inderdaad. Daar kun je zeggen van: oke, dit is dus de winnaar, deze heeft rank 1. | Ja. [vult 1 in de cel in] Uh ohw! Oke, maar nu is alles veranderd.  (…)  Hmmm, [klikt op tabblad ranking outcome] wat is er nu met ranking outcome gebeurd? Hey… Oke, maar hier is nu iets… Dat heeft ‘ie daar dus daar zelf neer gezet.  (…)  [scrollt verticaal] Ik heb het gevoel dat ik nu zelf moet kijken welke dan.. nu, zeg maar, het hoogste gemiddelde heeft. Even kijken, 38 lijkt dan de hoogste. [scrollt horizontaal] Staat ‘ie ook…? Nee hij staat niet op [inaudible]… Dat is deze dus nu. [selecteert de correcte cell voor de ranking, vult 2 in de cel boven één van de MULTI(2)] Wat is er nu gebeurd? [klikt op tabblad ranking outcome] Ranking outcome, ja dan staat die dus daar [wijst met cursor naar kolom patient score (kolom C), klikt op tabblad RRV]. Oke, uhm, ik ga gewoon proberen om.. [scrollt horizontaal] het aflopend gemiddelde tot 10 te ranken. Even kijken, 21,8.. 22 is het hoogste, 19, 15, 18.. Hier ook 22… 22,6. Oh wacht, er staat winnaar bij zelfs. [vult rank 3 in de correcte cel in] Wacht, misschien ben ik niet zo snugger. Ja, ohh kijk, dat is automatisch.[vult de ranks in de correcte cellen in] Vier, vijf, vijf modules geprioriteerd. Zes. [drukt op enter en de selectie verspringt naar beneden vanwege vergrendelde cellen] Waarom gaat ‘ie naar onder toe? Even kijken, zeven. Acht. Negen. Tien. | Ontdekt na aanwijzing hoe en waar de ranking in de tool plaats vindt.  [Discovers how and where manual ranking takes place in the tool] | Where and how to rank may become clear after a little instruction | Provide background infrmation to the tool’s automatic ranking in an accompanying document |
| 3 |  | Eh, ja. Dan deze dus 4 [vult correct 4 in]. [scrolt horizontaal] Dit wordt dan 5 [vult 5 in]. Eh [vult 6 in], op deze manier kun je dus ook aangeven hoeveel modules je wil eh, prioriteren. Dus hoeveel modules je wilt [scrolt horizontaal] meenemen eigenlijk. Deze zijn, oh dit is eh, ja, tot hier.. [klikt eerste cel in rij 2 aan die geen te ranken module bevat] gaan de items. [scrolt horizontaal] Dat zijn 19 modules?  (…)  7 [vult 7 in], [scrolt horizontaal] 8… [vult 8 in] Ho.. [vult 9 en 10 ook in] | Manier van ranken wordt iets helder door een richting in de opdracht  [Method of ranking becomes somewhat clear through some direction in the assignment] |  |  |
| 3 |  | Aha, oke [klikt op de juiste cel boven de winnaar in rij 2]. De rank als cijfer. Dus de winnaar wordt dan 1 [vult 1 in in de cel]. Ah, dat is het! Dit wordt dan 2 [vult 2 in in de juiste cel van een multi-winner, negeert MULTI(2) in rij 3]. Dan 3 [vult 3 in in de juiste cel]. Heel goed. | Manier van handmatig ranken wordt duidelijk door de opdracht maar negeert MULTI(2)  [Method of manual ranking becomes clear through the assignment, but ignores MULTI(2)] | It is not immediately clear how to deal with items tied for the same rank in the tool without additional instructions | When using the automatic ranking, we will ensure that Excel prompts a message when there is a tie that needs manual assignment. Additional information on an option how to deal with ties will be provided in an accompanying document (heterogeneity analysis). |
| 1 |  | Oh wacht deze [selecteert de cel boven een MULTI(2)] is dan nu gelijk tweede. Krijgen ze dan allebei een 2? [vult 2 in boven de cellen van MULTI(2)] | Onduidelijkheid over gelijke ranks: welke rank in te vullen?  [Lack of clarity about tied ranks: which rank to fill out?] |  |  |
| 4 |  | Dan geef ik ze allemaal dezelfde s.. score [vult 10 in op rij 2 bij een item met dezelfde ongewogen totaalscore], want ze hebben een gedeelde plek. Geen idee. | Vult bij gelijke ranks hetzelfde nummer in  [Fills out the same number for identical ranks] |  |  |
| 4 |  | Oh! De ger.. oh. [klikt op tabblad ‘ranking outcome’] Ik snap niet waarom er dan hier N/B komt? [wijst met cursor naar een item waar er een #N/B melding in Excel staat] Oh omdat ik 10 [klikt op tabblad RRV] had ik natuurlijk twee keer had gedaan omdat die allebij dezelfde score kregen. [klikt op tabblad ‘ranking outcome’] | Heeft bij gelijke rank twee keer dezelfde rank toegekend aan twee verschillende items en krijgt daarom foutmelding op ranking outcome tab  [Assigned the same rank twice to different items which causes the error on the ranking outcome tab] |  |  |
| 3 |  | Waar is die volgende winnaar nou gebleven? Hier. Ehm. Waar was ik? 6? [scrolt horizontaal] Nee 6 had ik gehad, 7 dus. | Handmatig ranken en zelf tel bijhouden van ranks kan foutgevoelig zijn  [Manual ranking and keeping up with the numbered ranks may be prone to errors] | Scrolling horizontally to find winners and manually keeping track of the ranking order may prove to be difficult | We will automate the ranking process in the next iteration of the tool. |
| 1 |  | Max score is 10… Moet je dit dan nu zelf met de hand gaan doen? Dat lijkt mij nogal veel werk. Lijkt mij makkelijker als het automatisch kan gaan. Ehm… Maar wat ik zo kan zien staat nog nergens, automatisch. [ wijst naar cellen met somscores van patiënten] | Wens voor automatisering van de ranking  [Wish to automate the ranking method] | Wish for an automatic ranking system/method | We will automate the ranking process in the next iteration of the tool. |
| 2 |  | Maar wat ik [klikt op tabblad RRV] wel een beetje vreemd vind, is dat je dat je dus handmatig.. [selecteert de cel naast rank 1] dat moet doen. Ik snap niet zo goed waarom ‘ie dan niet zelf automatisch die dingen van hoog naar laag kan sorteren. | Wens om ranking te automatiseren  [Wish to automate the ranking] |  |  |
| 4 |  | [klikt op tabblad ‘voters’] Nee, hier moet ik wel even zeggen hoe veel mensen er stemmen. Dus dan… Van de <*wetenschappelijke vereniging 1*> is er één iemand [telt in dataset, vult 1 correct in in de tool], van de <*wetenschappelijke vereniging 2*> zijn er 2 [telt in dataset, vult 2 correct in in de tool], van de <*wetenschappelijke vereniging 3*> zijn er 4? [telt in dataset] Zou misschien handig zijn als je dat soort van automatisch uit kan vissen ofzo. [vult 1 correct in in de tool] | Wens om aantal voters te automatiseren  [Wish to automate the count of the number of voters] | Wish for an automatic method to count the number of participating voter | It is possible to automate counting the number of voters. However, its purpose was to adjust the weighting method for individual weights. We have decided that we will use a different weighting method independent of the number of voters per organization. Therefore, the number of voters per organization does not have to be filled out in the next iteration of the tool. |
| 1 |  | Oke. [klikt op tabblad ranking outcome] Dus daar moet ik ook iets in kunnen doen. [klikt op tabblad RRV, selecteert gele cellen ‘mark when ≤’] Oke, staat er ergens… De mean is vooral 5 als ik het even zo grofweg bekijk. [inaudible] .. 10.. Dit gaat over de variantie. De variantie is gemiddeld 8, 7, hmm 11, 8. [vult 8 in de cel ‘mark when ≤’ voor variantie] Als ‘ie kleiner is dan 8, of als ie groter is dan.. [selecteert cel ‘mark when ≥ voor variantie] Kan natuurlijk niet hetzelfde.. 8,1 [vult 8,1 in de vel ‘mark when ≥’ voor variantie]. Ach, [inaudible] 8,01 we [inaudible] risico [vult 8,01 in de cel ‘mark when ≥’ voor variantie en selecteert ‘mark when ≥’ voor std. deviatie]. Ja. Nu zie ik welke hoger dan 8 variantie hebben.. en lager dan 8. | Werking van de heterogeniteits-analyse meteen zelf ontdekt  [Functioning of the heterogeneity analysis was discovered without instructions] | Use and functioning of the heterogeneity analysis is generally clear | - |
| 2 |  | En volgensmij moet je hier [selecteert de cel ‘mark when ≤’ voor variantie] kunnen aangeven wat, eh. Dat je een kleurtje of een markering wil als er ‘ie een bepaalde spreiding boven of onder een bepaald getal heeft.  (…)  Oke, ik zeg alles wat onder de 8 zit [vult 8 in de cell ‘mark when ≤’ voor variantie in], en wat uhm, ehh, wacht, nee dit is alles onder de 8. Ahh wacht [verwijdert 8 in de cell ‘mark when ≤’ voor variantie, selecteert ‘mark when ≥’ voor variantie]]. En dan hier doe ik [vult 8 in de cell ‘mark when ≥’ voor variantie in] alles boven de 8. [scrollt horizontaal] Hmm kijk, dat zijn er dus best wel veel. Dus je kan checken.  (…)  Ehm, dus je kan hier [verwijderd 8 en vult 10 in de cel ‘mark when ≥’ in] gewoon handmatig eh, zeggen: variatie meer dan 10. Dan zie je dat er nog best wel wat items zijn die een hoge … [scrollt horizontaal] variantie hebben.  (…)  Hmm, wat is dit? [wijst met cursos naar cellen ‘mark when’ voor kwartielgrootten] Oh dat gaat over de kwartielen. Oke, en wat is dit nog? [selecteert de cel ‘mark when ≥’ voor std deviatie] Doet dit ook nog iets? Dit is de, oh, de standaarddeviatie. [vult 1 in de cel ‘mark when ≥’ voor std deviatie]Oke. | Zelf relatief eenvoudig ontdekt hoe de heterogeniteitsanalyse werkt  [Functioning of the heterogeneity analysis was discovered relatively easy without instructions] |  |  |
| 3 |  | [scrolt naar boven] Ehh.. Ohja, hier [ wijst met cursor naar de heterogeniteitsanalyse weergave van de items] geeft ie eh.. hier geeft ‘ie daar informatie over. [klikt op gele cel ‘mark when ≤’ voor variance in rij 12] Oke, dus als de variantie groot is, eh en de standaard deviatie groot is, ehm.. dan kan ik dat hiermee [klikt op gele cel ‘mark when ≤’ voor standaard deviatie in rij 13], dan kan ik dat hiemee laten… even kijken hoor. Ehm. Even kijken welke nou een grote eh.. standaard deviatie hebben. [scrolt horizontaal even heen en terug][inaudible] Nou laten we bijvoorbeeld eens eh, als ‘ie tussen de.. 3.. [vult 3 in gele cel ‘mark when ≤’ voor standaard deviatie] en 3.5 is. [inaudible] [vult 3,5 in rode cel ‘mark when ≥’ voor standaard deviatie] | Gebruikt uit zichzelf de heterogeniteits-analyse correct, zonder aanwijzingen  [Correctly uses the heterogeneity analysis without instructions] |  |  |
| 4 |  | Markeer wanneer het groter is dan… [klikt op cel ‘mark when ≤’, scrolt naar beneden, scrolt naar boven] dan… zes [vult 6 in bij ‘mark when ≤’ voor variantie] en kleiner dan 2 [vult 2 in bij ‘mark when ≥’ voor variantie]. Groter dan 6. Alles is eigenlijk groter dan 6. Nee maar waarom gaat deze dan, waarom wordt deze [wijs met cursor een rood gemarkeerde cel aan] dan rood. Deze is 4,75. En… kleiner dan… 1 [vult 1 in bij ‘mark when ≥’ voor variantie]. Huh? [verwijdert de 1 in de cel van ‘mark when ≥’ bij variantie] Markeer wanneer, nu wordt ‘ie r… groter dan 2 [vult 2 in bij ‘mark when ≤’ voor variantie]. Maar dan zegt ‘ie niks. Groter dan 6 [vult 6 in bij ‘mark when ≤’ voor variantie]. Wil gewoon groter dan [vult 8 in bij ‘mark when ≤’ voor variantie] acht, hier. He? Waarom wordt het dan 7,10 [wijst naar een geel gemarkeerde cel], dat is toch… kleiner dan 8, ohja. Kleiner dan… acht. [vult 8 in bij ‘mark when ≤’ voor standaard deviatie] Kleiner dan 8. Is alles. [verwijdert 8 in de cellen van ‘mark when ≤’ bij variantie en standaard deviatie] Kleiner dan 2 is bijna niks. [vult 3 in bij ‘mark when ≤’ voor variantie] Kleiner dan drie en of groter dan 8 [vult 8 in bij ‘mark when ≥’ voor variantie]. Kleiner dan 3 of groter dan 8? Wat was ook al weer de opdracht? Groter? | Gebruikt het principe van heterogeniteits-analyse correct, maar draait ≥ en ≤ om in het gebruik.  [Uses the principles of the heterogeneity analysis correctly, but confuses ≥ and ≤] |  |  |
| 3 |  | Hoe groter de spreiding, ehm, ja, wat zegt dat? Dat mensen er heel verschillend over denken, in feite. Ehm. Ja, en wat ga je dan uiteindelijk met die informatie doen? Dat betekent in ieder geval dat er discussie over zal zijn… maar is dat dan een reden om ‘m wel of niet te prioriteren? | Vraagt zich af wat het nut is van de heterogeniteits-analyse  [Wonders what the use of the heterogeneity analysis is] | It may be unclear why one wants to use the heterogeneity analysis | We will provide additional information in an accompanying document about a possible role of the heterogeneity analysis (ranking with ties). |
| 1 |  | De lijst met ranking.. [klikt tabblad ranking outcome aan] Die had ik hier weer gezien. Dus volgens mij kan ik die heel makkelijk kopiëren [kopieert de ranking outcome] naar een.. Word-bestand. Word-bestand zei je he? [plakt de ranking in een Word bestand] | Correcte en eenvoudige output uit de tool  [Correct and easy output from the tool] | Getting the output out of the tool is generally easy to perform | - |
| 2 |  | Oke. Ehh, [klikt op tabblad ranking outcome] ik ga naar het tabblad.. [selecteert de correcte cellen] van eh.. ranking outcome en ik ga dit gewoon selecteren en even [kopieert de tabel] control-c doen en dan kijk ik eventjes of ik dit in een Word-document kan plakken. Maar volgens mij kan je het niet zien dat ik dit nu open. Ja, ik heb het geplakt. Het staat gewoon in een word-document, dus ik kan het zo, eh.. | Output: locatie voor output helder en daarna eenvoudige output uit de tool  [Location for output of the tool was clear, and thereafter the output of the tool was easy] |  |  |
| 3 |  | Oke. [klikt op tabblad ‘ranking outcome’] Nou, dit is de ranking. Ehm. [selecteert de cellen met de header rank, name, patientscore en de bijbehorende gerankte items] Als ik dat gewoon copy-paste in Word… Dan krijg ik dus een lijstje met de volgorde waarin ‘ie geprioriteerd is. | Output: eenvoudige manier om output uit de tool te krijgen  [Easy output out of the tool] |  |  |
| 4 |  | Nou, dan ga ik [selecteert en kopieert 13 items met rank / naam / patiënt score], dit zou ik gewoon kopiëren. Ehh… Ja gewoon plakken. [plakt de lijst in Word] | Output: eenvoudige manier van output uit de tool krijgen  [Easy way of getting output out of the tool] |  |  |
| 4 |  | Dat ik dit zeg maar [selecteert de eerste 5 items in de tool] even naar beneden… [verplaatst de geselecteerde items 1 rij naar beneden] Ik zal denk ik zo doen zeg maar [kopieert en plakt de richtlijntitel vanuit de dataset naar de tool in de lege rijd die door het verplaatsen is ontstaan], dan toch maar.  (…)  Ja, maar waarom krijg ik dan hier… [wijst met cursor naar een item in rij 2 met een #VERW! melding door Excel] Oh dat mag je natuurlijk niet zeggen, maar ik zou nu denken: eh, waarom krijg ik dan hier verwijderd? | Verplaatsen van cellen binnen de tool is niet te adviseren  [Moving cells within the tool not advisable] | It is not advisable to move or cut/paste data within the tool to prevent errors | We will provide additional information in an accompanying document that will advise against moving or cutting/pasting cells within the tool. |
| 4 |  | Zo [verwijdert de richtlijn titel van richtlijn 2], zo [verwijdert de richtlijntitel van richtlijn 1, selecteert de module namen van richtlijn 1, knipt en plakt de geselecteerde modulenamen 1 rij naar boven, selecteert de module namen van richtlijn 2, knipt en plakt de geselecteerde module namen 2 rijen naar boven, verwijdert de naam van richtlijn 3, selecteert de modulenamen van richtlijn 3, knipt en plakt de geselecteerde modulenamen 3 rijen naar boven]. Zo, nu moet het wel kloppen denk ik [klikt op tabblad RRV]. Alleen nu zie ik hier ‘verwijderd’ [wijst naar #VERW! In rij 2 van een aantal items], maar dat is denk ik, eh, ahh. | Knippen/verplaatsen binnen de tool is niet te adviseren  [Cut/paste within the tool is not advisable] |  |  |
| 4 |  | Als je dan hier bijvoorbeeld invult [klikt op tabblad ‘voters’]: <*wetenschappelijke vereniging 1*> 1. Wat gaat er dan gebeuren? [klikt op tabblad RRV] Gebeurt er dan wat? [klikt op tabblad ‘voters’, vult 1 bij <*wetenschappelijke vereniging 1*> correct in, klikt op tabblad ‘RRV’] Zou het even… [klikt op tabblad ‘voters’] Is dat zinvol om te doen?  (…)  Ehhh… Nou dan moet ik bijvoorbeeld kijken waar dan meerdere <*wetenschappelijke vereniging 2*> mensen op gestemd hebben, dus dat is dan… Ohja, dat is natuurlijk bij alle modules. Ehm pff [klikt op tabblad ‘voters’] zie ik wat veranderen? [klikt op tabblad RRV] Ehmm… 3.1, 3.2, 3.4 [klikt op tabblad ‘voters’] Ja, [klikt op tabblad RRV] eh drie, ja volgens mij wel: 3.1 zei ik net 3.2, 3.4. [klikt op tabblad ‘voters’] Als ik dan hier doe, [klikt op tabblad RRV] nu staat er: 3.3, 3.3, 3.5 [klikt op tabblad ‘voters’, klikt op tabblad RRV] / 3.3, 3.3, 3.5, nee heh? [klikt op tabblad ‘voters’] Nee. [klikt op tabblad ‘RRV’] | Tool geeft geen feedback over wat er gebeurt met aantal voters en de invloed op de weging  [The tool does not provide feedback about what happens with the number of voters and its influence on the weighting method] | The tool does not provide real-time feedback as to what happens with the number of voters and its influence on the weigthing method | We have decided that we will use a different weighting method independent of the number of voters per organization. Therefore, the number of voters per organization does not have to be filled out in the next iteration of the tool. |

**S3 Table 4. Part 4: Using the tool in scenario 2**

| **P** | **Additional instruction** | **Participant text** | **Label/observation** | **Interpretation** | **Actions to improve** |
| --- | --- | --- | --- | --- | --- |
| 1 |  | Oke. Modulelijst… Krijg nou eerst alle modules weer… [kopieert en plakt module namen uit scenario 2 naar de correcte cellen in tabblad labels list] Dat zijn maar twee richtlijnen zo te zien. [kopieert en plakt overige module namen uit de lijst in de daarvoor bestemde cellen] | Input: zelfstandig itemnamen in tool gevoerd  [intem names were independently entered into the tool] | Input of data into the tool is easy to perform | - |
| 1 |  | De naam… [kopieert alle namen uit scenario2] Het staat net andersom, na de vereniging. [plakt alle namen in de RRV tool in de correcte cellen] [selecteert en kopieert alle verenigingen uit scenario2] Kopiëren… [plakt alle verenigingen in de correcte cellen in de RRV tool] En dan kunnen we de scores overzetten… [kopieert alle scores in scenario2] En plakken. [plakt de scores in de correcte cellen] | Input: correcte input van deelnemers, organisaties, scores  [Correct input of participants, organizations, scores] |  |  |
| 2 |  | Ja, [plakt de modulenamen in de correcte cellen] even kijken, [scrollt verticaal] postoperatief beleid moet de laatste zijn die ik geselecteerd heb. Dan nog oesofagus [inaudible] carcinoom. Ehhm, [plakt modulenamen inde correcte cellen] die plak ik er ook in. [scrollt verticaal] Oke, even kijken. Uit scenario 2: ik heb alle modules, dan heb ik een database, ik heb een vereniging. Die ging ik eerst plakken. En.. [klikt op tabblad RRV] die ging ik op de RRV pagina doen, oeps. Even kijken bij [plakt de verenigingen in de correcte cellen] organisatie. En ik heb dan de namen van alle dokters. [plakt de deelnemersnamen in de correcte cellen] Die plak ik er ook in, en dan… moest ik nog iets plakken. Natuurlijk de scores.  (…)  [plakt alle scores in de correcte cellen]. Eh, alle scores staan er nu in. | Input: voert geheel zelfstandig direct alle data correct in de tool in  [Immediately and independently fills out all input data correctly into the tool] |  |  |
| 3 |  | klikt op tabblad ‘labels list’] Oke [plakt item namen in de correcte cellen, scrolt naar beneden]. Dit zijn er ook echt, eh, wat meer.  (…)  [plakt een tweede set modules aansluitend in de lijst]. Even kijken, dit zijn alle modules [klikt op tabblad ‘RRV’]. Zo. En dan de… eh.. Ohja. De namen, en verenigingen. Ik begin met de namen. [inaudible] kopiëren. [plakt alle namen in de correcte cellen] Zo. Organisaties… Verenigingen.  (…)  plakt alle verenigingen in de juiste cellen]  (…)  Ehm, dan de scores. Ja, het zijn nog best wel wat modules.  (…)  [plakt de matrix met scores in de juiste cellen] | Input: eenvoudige manier om input in de tool te krijgen  [Easy to place input into the tool] |  |  |
| 4 |  | Oke, en dan dit [wijst me cursor naar modulenamen in de database] moet je, dus dit is dan de item-lijst [selecteert en kopieert de modulenamen van de eerste richtlijn in de database].  (…)  Hmm. [opent de tool, klikt op tabblad ‘labels list, plakt modulenamen correct in de lijst, selecteert en kopieert de modulenamen van de tweede richtlijn in de database] Dit dan daar [plakt modulenamen correct in de lijst]. En dan deelname… [selecteert cellen met deelname aantallen in de dataset] Oh dit kan je dan, dit is nu dan wel nodig. Ehm.. die tool [opent de tool, klikt op tabblad ‘voters’], met alle mensen die stemmen. <*wetenschappelijke vereniging 1*> 2 [vult 2 correct in], <*wetenschappelijke vereniging 2*> 1 [vult 1 correct in], <*wetenschappelijke vereniging 3*> 3 [vult 3 correct in], <*wetenschappelijke vereniging 3*>.. hee, ohja hier. 1 [vult 1 correct in]. <*wetenschappelijke vereniging 5*> 1 [vult 1 correct in], <*wetenschappelijke vereniging 6*> 1 [vult 1 correct in], <*wetenschappelijke vereniging 7*> 9 [vult 9 correct in], <*wetenschappelijke vereniging 8*> is 2 [vult 2 correct in], <*wetenschappelijke vereniging 9*> is 1 [vult 1 correct in], <*wetenschappelijke vereniging 10*> is 2 [vult 2 correct in], <*wetenschappelijke vereniging 11*> is 2 [vult 2 correct in], de <*wetenschappelijke vereniging 12*> is 1 [vult 1 correct in]. En dan moest ik [klikt op tabblad ‘RRV’] dit nog even doen. Naam [selecteert en kopieert de kolom met namen uit de dataset, plakt deze correct in de tool]. Vereniging [selecteert en kopieert de kolom met verenigingen uit de dataset, plakt deze correct in de tool]. [selecteert en kopieert de matrix met scores uit de dataset, plakt deze correct in de tool] Scores. | Input: vult de input direct op je correcte plaats in de tool in  [Fills out the input data corretly into the tool] |  |  |
| 1 |  | [klikt tabbladen outcome ranking, RRV, outcome ranking aan] Even kijken, de ranking staat dan hier [selecteert en kopieert de gehele ranking in tabblad ranking outcome]… Pak gewoon weer even hetzelfde Word-bestand. Ik haal deze weer weg [verwijdert tabel van scenario1]. [plakt en formatteert de gerankte lijst in Word] Eh, als tabelletje vind ik fijner. Ja. [klikt tabblad RRV aan in de tool] | Output: eenvoudige output uit de tool  [Easy output out of the tool] | Output of data out of the tool is easy to perform | - |
| 1 |  | [klikt tabblad ranking outcome aan] Even kijken, want zou eerst gelijk hetzelfde als de andere lijst. [kopieert de hele ranking] Maar als ik ‘m hier onder plak [plakt de ranking onder de tabel zonder penaltymethode in Word]… dan zou ik denk ik verschil moeten zien. | Output: eenvoudige output uit de tool  [Easy output out of the tool] |  |  |
| 2 |  | [klikt op tabblad ranking outcome] Oke. Even kijken, ik heb hier 15 eh.. [selecteert de correcte cellen voor de top15 om te kopieren] dingetjes geselecteerd.  (…)  [kopieert de correcte cellen]  (…)  Ik open even mijn word, eh-documentje. Ja, hij staat nu in Word. | Output: eenvoudige output uit de tool  [Easy output out of the tool] |  |  |
| 2 |  | [klikt op tabblad ranking outcome, selecteert de correcte cellen] Oke, [kopieert de geselecteerde cellen] dan heb ik weer geen patiënten die, eh.. Plak ik in hetzelfde Word-bestandje. | Output: eenvoudige output uit de tool  [Easy output out of the tool] |  |  |
| 3 |  | Ja. [ klikt op tabblad ‘Ranking outcome’, selecteert en kopieert de gerankte modules (rank / name / patient score) zonder header] Dan copy-past ik dit even. Zo.. Ja..? | Output: eenvoudige output uit de tool  [Easy output out of the tool] |  |  |
| 3 |  | Dit lijstje kopiëer ik dan naar mijn Word-bestand. [klikt op tabblad ‘ranking outcome’] Ranking outcome. Zo [selecteert en kopieert alle ranks / names / patient scores onder header]. | Output: eenvoudige output uit de tool  {Easy output out of the tool] |  |  |
| 4 |  | [selecteert en kopieert de gerankte lijst met rank / name / patient score zonder header] Maar ik weet niet wat dat dan… Ehm… [plakt de gerankte lijst in Word] Eh, ik doe het gewoon even helemaal volgens het boekje, zo [herstelt de opmaak van de gerankte lijst in Word]. | Output: eenvoudige output uit de tool  [Easy output out of the tool] |  |  |
| 4 |  | En dan is het denk ik de bedoeling dat ik nu weer hier dit [selecteert de cellen met rank / name / patient score van de gerankte items], deze lijst kopieer/plak?  (…)  [kopieert de geselecteerde cellen] En dan daar onder, dan kan ik het goed met elkaar vergelijken straks.  (…)  [plakt de lijst met een andere opmaak in Word, verwijdert de lijst] Ho, [onverstaabaar, plakt de lijst met de oorspronkelijke opmaak in Word]. | Output: eenvoudige output uit de tool  [Easy output out of the tool] |  |  |
| 4 |  | [scrolt horizontaal, klikt op tabblad ‘ranking outcome’, selecteert en kopieert de cellen van items met rank/ name / patient score zonder header, plakt de lijst in word] Ehm… [verwijdert de lijst in word, plakt de lijst in word met de oorspronkelijke opmaak]] Zet ik ‘m er naast, dan kan ik beter vergelijken. | Output: eenvoudige output uit de tool  [Easy output out of the tool] |  |  |
| 1 |  | Ja. [klikt op tabblad deelname in scenario2] En hier staat.. oh deelname. Mooi dan kan ik het mooi overnemen. <*wetenschappelijke vereniging 1*> heeft er 2 [vult aantal stemmers in], de <*wetenschappelijke vereniging 2*>… heeft er 1, <*wetenschappelijke vereniging 3*>…3. Dit zou misschien makkelijker moeten kunnen, maar.. | Aantal voters invullen lijkt onhandig  [Filling out the number of voters seems inconvenient] | Filling out the number of participating voters seems to be inconvenient en potentially prone to errors | We have decided that we will usr a different weighting method independent of the number of voters per organization. Therefore, the number of voters per organization does not have to be filled out in the next iteration of the tool. |
| 2 |  | En… [klikt op tabblad voters] Ik ga even naar de voters. Ah ja, en nu vind ik het wel onhandig dat ik dat handmatig bij de vereniging moet zoeken. Even kijken waar dat staat. <*wetenschappelijke vereniging*> zijn er 2, kan ik dit niet slimmer doen? <*wetenschappelijke vereniging*>.. [zoekt <*wetenschappelijke vereniging*> in de lijst] pfff. Hmmm. [vult 2 in de correcte cel in] Oh wacht, ik ga even mijn schermen naast elkaar zetten want dan kan ik het veel makkelijker lezen [wijzigt schermweergave in windows].  (…)  Het staat op alfabetische volgorde dus dat is toch wel iets handiger, maar.. wel een beetje zoeken en ik denk ook ook zo’n beetje fout gevoelig. | Invullen van aantal voters lijkt onhandig en onverzichtelijk te zijn  [Filling out the number of voters seems to be inconvenient] |  |  |
| 3 |  | Ja. [inaudible] [klikt op tabblad ‘voters’] Eh, ja dus <*wetenschappelijke vereniging 1*> twee dat klopt inderdaad. Dat staat daar ook. <*wetenschappelijke vereniging2*>.. eh, <*wetenschappelijke vereniging 2*> 1, <*wetenschappelijke vereniging3* > 3. <*wetenschappelijke vereniging 3*> 3…[vult 3 in de correcte cel in] <*wetenschappelijke vereniging 2*> 1.. [vult 1 correct in] Ehm.. <*wetenschappelijke vereniging 4*>, <*wetenschappelijke vereniging 5*> allemaal 1… [vult beide correct in] Ja. <*wetenschappelijke vereniging 6*> 5, <*wetenschappelijke vereniging 7*> 9 [vult beide correct in]. Ja zie je, de <*wetenschappelijke vereniging 7*> is inderdaad met veel en dat zie je ook wel echt terug in de uitkomst. En de <*wetenschappelijke vereniging 8*> 2 [vult 1 in]. Hmm. Even kijken hoor, waar staat ‘ie? [vult 1 in voor <*wetenschappelijke vereniging 9*>] Ehm, dit waren er 2 [vult 2 in voor <*wetenschappelijke vereniging 9*>]. Nee, wacht nu doe ik het verkeerd. 2 en 1. [vult 2 in voor <*wetenschappelijke vereniging 7*>, vult 1 in voor <*wetenschappelijke vereniging 9*>] Ehm. <*wetenschappelijke vereniging 10*> 2, <*wetenschappelijke vereniging 11*> 2… [vult 2 in voor <*wetenschappelijke vereniging 10*>. Hier, 2 [vult 2 in voor <*wetenschappelijke vereniging 11*>]. Het staat natuurlijk op alfabetische volgorde. En dan de <*wetenschappelijke vereniging 12*> nog 1, dat is de laatste. Hier. [vult 1 in voor <*wetenschappelijke vereniging 12*>] Oke. In totaal 30 [scrolt naar boven en naar beneden], dat klopt volgens mij ook nog [klikt op tabblad ‘RRV’]…. Met het aantal stemmers. Oke. | Handmatig invullen van aantal voters lijkt onhandig en foutgevoelig  [Manually filling out the number of voters could be inconvenient and prone to errors] |  |  |
| 1 |  | Top, volgens mij staat het er zo in. [klikt tabblad RRV aan] Staat er dan hier [scrollt verticaal] ook iets bij? Niet dat ik zo kan zien. Oke. [klikt tabblad ranking outcome aan] Het staat er wel in nu. [klikt tabblad voters aan] | Tool geeft geen feedback over wat het doet met het aantal voters  [Tool does not provide feedback regarding the number of voters] |  |  |
| 1 |  | [selecteert de rij met ranks] Deze kan dus weer weg. [verwijdert de inhoud van de geselecteerde cellen] Is nu alles weg? [scrollt horizontaal, verwijderd laatste cellen met ranks] Nee. Ja hij is weer leeg, oke? | Verwijderen van de ranks in de tool moet handmatig gebeuren en lijkt onoverzichtelijk door horizontaal scrollen  [Deleting the ranks is a manual task and seems inconvenient by scrolling horizontally] | Deleting ranks from the designated row in the tool seems inconvenient | A button to automatically clear all ranks will be developed in the next iteration of the tool, so that this won’t have to be performed manually. |
| 1 |  | Dan vul ik hier 2 in [vult 2 in de correcte cel in]. En gebeurt er dan wat? [scrollt naar boven voor de somscores/ranking/winner] Oh, en dan moet ik misschien ook dit invullen [klikt op cel waar penalty aggression ingevuld kan worden], aggression.. Met 2.. null.. simple reweighted… | Tool geeft geen feedback over wat het doet bij invullen van penalty method en aggression  [Tool does not provide feedback about how it uses the penalty aggression] | The tool does not provide real-time feedback when tool parameters change, since most formulas are calculated/used in the background. There is a desire to see/know more about the penalty method and/or penalty aggression. | Because most formulas operate in the background of the tool it is dificult to provide real-time feedback when tool parameters are changed. We will provide additional information on the penalty method and the penalty aggression in an accompanying document. |
| 2 |  | Wat is penalty aggression dan? Dat is… Only applicable to method 2, a penalty aggression of 0 equals a reweighted range voring method without weights. Wat dat is, echt geen idee? Maar ik kan dus volgens mij gewoon twee soorten penalties geven. | Geen achtergrond-informatie over penalty aggression  [There is no additional information about the penalty aggression] |  |  |
| 2 |  | [vult 1 in de correcte cel voor penalty method] Ik kan een eentje geven. Even kijken wat er dan gebeurt. [scrollt verticaal, scrollt horizontaal] Wacht even, nu weet ik niet meer welke module de winnaar was met de gewone penalty [vult 0 in de correcte cell voor penalty method]. Ik zet ‘m even terug op 0. [scrollt virticaal, scrollt horizontaal] Dat was de herstadiëring. En als ik een ééntje geef [vult 1 in de correcte cel voor penalty method in] dan doe ik dus alleen op basis van statische groepsgrootte. [scrollt horizontaal] Dan krijg ik… een andere module… nee dezelfde. Shit ik weet het niet meer. Even kijken, [vult 2 in de correcte cel voor penalty methode] twee. [scrollt horizontaal] Ah, ik weet niet of ik nu niet scherp ben of dat ik nou niet goed zie. Wacht even, [vult 0 in de correcte cell voor penalty methode] nul..  (…)  Maar dan lijkt er in de winnaar nu niks te veranderen met de penalty die ik geef. Waarschijnlijk dan andere dingen, maar dat heb ik dan niet gechecked. [klikt op tabblad ranking outcome] Kan ik dat bij mijn ranking zien? | Onvoldoende achtergrond over werking pentaly methode  [Not enough information provided about the penalty method] |  |  |
| 3 |  | En.. Dan zouden we dus kunnen spelen van: wat er gebeurt als je dan op 1, als je daar 1 van maakt of 2 van maakt, of.. nou, hoeveel, 23 van maakt. | Geen kader in de tool gegeven van de welke mate van aggression te kiezen  [No background provided for which magnitude of aggression to use] |  |  |
| 3 |  | Herstadiëring na neoadjuvant. Ehm.. Dat is nog steeds wel dezelfde winnaar. | Achtergrond-informatie over wegingsmethode is wenselijk (1^e^ winnaar is altijd hetzelfde)  [First winner is always the same. Background information about the weighting methods seem desirable] |  |  |
| 3 |  | En neoadjuvante chemoradiatie was hier twee. En dat is hier … ook twee. Minimale invasieve oesofagusresectie is drie. Dat was ook al zo. Hé dit lijkt toch niet zo heel anders. Chemotherapie … chemoradiatie, endoscopische behandeling. De top 5 lijkt eigenlijk niet… ehm. Oh nee wacht. Type anastomose <*wetenschappelijke vereniging*> staat hier op 5. En hier [wijst met cursor naar #5 in de ranking] staat een andere op 5. Oke, dus de top-4 is eigenlijk hetzelfde nog steeds. | Geen kader in de tool gegeven van de welke mate van aggression te kiezen (door a=2 is er weinig verschil)  [No background provided for which magnitude of aggression to use (a=2 causes little difference)] |  |  |
| 4 |  | Maar is het dan de bedoeling dat, dat bedenk ik mu nu he [misklik waardoor het werkblad verschiet, scrolt horizontaal en verticaal terug], dat als je dit dan zo meteen gaat doen met je cluster, dat je dit dan zelf inschat wat dan de waardes zijn? Maar goed, dat mag je natuurlijk nu nog niet [onverstaanbaar] [verwijdert rank 1 uit rij2, werkblad nog steeds op 60% grotte]van oke, waar is dat dan op gebaseerd [verwijdert rank 2 uit rij 1] en hoe [vult 1 correct in op rij 2] moet ik dat dan straks kiezen? [vult 2 correct in op rij 2] En wat zegt dat [vult 3 correct in op rij 2], wat houdt het in? | Onduidelijkheid over welke waarde van de aggression ingevuld moet worden, tool geeft geen info  [Unclear which value of aggression is needed, the tool does not provide information] |  |  |
| 1 |  | Eén tot en met 15, oke. [vult rank 1 in] Het is een stuk lastiger zoeken als het er zo veel zijn… | Automatische identificatie winnaar: winnaar is lastig te vinden bij veel items  [The automatic identification of the winner is hard to find when there are lots of items] | Ranking manually is more difficult when there are lots of items present in the tool. Participant sneed to scroll horizontally often to follow the changing winner after assigning a rank. | We will automate the ranking process in the next iteration of the tool. This way, the changing winner does not have to be followed in the work sheet and a rank is assigned by clicking on a button. |
| 1 |  | Oke, dit [selecteert de cel boven de winnaar] is volgens mij nog steeds dezelfde Winnaar [vult 1 in als rank boven de winnaar]. Een, twee, drie, [vult ranking verder in] Ik wilde het veel te snel doen en dan is het frustrerend je niet gelijk die… Vijf, zes, zeven, acht, negen, volgens mij is het nu wel anders als eerst. Elf, twaalf, kijken.. dertien, veertien. En vijftien waar ben je? Daar. Yes. | Handmatig ranken lijkt onoverzichtelijk te zijn (horizontaal scrollen bij veel items en zelf ranks tellen)  [Ranking mannually seems inconvenient (scrolling horizontally when there are lots of items)] |  |  |
| 2 |  | Zoomen, [zoomt uit in het Excelbestand naar 40%] kan ik misschien iets sneller zien waar de winnaar zit. | Handmatig ranken bij veel modules is mogelijk onoverzichtelijk  [Manual ranking with lots of items is possibly inconvenient] |  |  |
| 3 |  | Het is nog best wel een klusje eigenlijk [vult 6 correct in]. Zeker als het een hele grote.. eh [scrolt horizontaal, vult 7 correct in]], een heel groot cluster is. Nouja hoewel [vult 8 correct in], aan de andere kant, ehm, hangt het natuurlijk ook af van hoeveel modules je kan prioriteren [scrolt horizontaal]. En dan valt het wel mee hoe groot dit klusje is. 9 [vult 9 correct in, scrolt horizontaal] | Handmatig ranken kan misschien een flinke klus zijn bij veel items  [Ranking manually might be a big chore with lots of items] |  |  |
| 3 |  | [scrolt horizontaal, vult 2 correct in] Ho, ik klik iets te ver door [klikt verkeer op de scoll bar]. Ben de tel kwijt [lacht]. Drie… Volgens mij was het drie. Wat gebeurde er nou? Drie [vult 3 correct in, scrolt horizontaal], vier [vult 4 correct in], vijf [vult 5 correct in, scrolt horizontaal, vult 6 zin, scrolt horizontaal]… Zeven [vult 7 correct in, scrolt horizontaal], acht [vult 8 correct in], negen [vult 9 correct in], tien [vult 10 correct in, scrolt horizontaal], elf [vult 11 correct in], twaalf [vult 12 correct in, scrolt horizontaal, vult 13 correct in]… veertien [vult 14 correct in], en de laatste. Vijftien [vult 15 correct in]. | Veel scrollen om de verspringende winnaar te volgen  [Lots of horizontal scrolling to follow the changing winner] |  |  |
| 4 |  | [scrolt horizontaal] Oh, dat gaat dan… Oh! [vult 2 correct in op rij 2, scrolt horizontaal] Oh, dan moet je me maar een keer uitleggen hoe dat dan gaat, maar goed. Eh! [lacht, vult 3 correct in op rij 2]  (…)  Vier [vult 4 correct in op rij 2], dat gaat iets sneller dan toen net [vult 5 correct in op rij 2]. Zes [vult 6 correct in op rij 2, scrolt horizontaal]. 7 [vult 7 correct in op rij 2], 8 [vult 8 correct in op rij 2], 9 [vult 9 correct in op rij 2], 10 [vult 10 correct in op rij 2, scrolt horizontaal], ehmm, 11 [vult 11 correct in op rij 2, scrolt horizontaal]. He, 12 [vult 12 correct in op rij 2, scrolt horizontaal]. 13 [vult 13 correct in op rij 2, scrolt horizontaal]. 14 [vult 14 correct in op rij 2] vijftien [vult 15 correct in op rij 2, vult 16 correct in op rij 2]. | Veel horizontaal scrollen om verspringende winnaar te volgen  [Lots of horizontal scrolling to follow the changing winner] |  |  |
| 4 |  | Ehm nou ik ehm, jaa. [verkleint het werkblad naar 60%] Ik denk nu gewoon vooral zo snel mogelijk invullen [scrolt horizontaal, vult 9 correct in op rij 2, vult 10 correct in op rij 2]. Heel veel meer dan dat denk ik niet, denk ik [vult 11 correct in op rij 2]. | Verkleint het werkblad om de verspringende winnaar te kunnen volgend voor de handmatige ranking  [Zooms out in the work sheet to follow the changeing winner fort he manual ranking] |  |  |
| 2 |  | Ik heb hier weer de gewogen gemiddelden en ik heb een winnaar [scrollt horizontaal]. Ehm, ik ben alleen even vergeten: moest ik dit nu ook gaan ranken als nummer 1? [vult 1 in de correcte cel voor de ranking in (boven ‘WINNER’)]  (…)  Oke. [vult de ranks in de correcte cellen in] Twee… [scrollt horizontaal]Waar zit nummer 3. Whoops, 3. Vier, Vijf, | Voert ranking zelfstandig en correct uit  [Carries out het ranking independently and correct] | Carrying out the ranking is generaly easy with the prior experience of getting to know the tool/ scoring / scenario 1. | We will automate the ranking process in the next iteration of the tool and provide guidance in an accompanying document. |
| 2 |  | Oke. Ehm, even kijken. [vult de ranks in de correcte cellen in] Een eentje.  (…)  Even.. Drie, volgens mij is dit inderdaad een hele andere nummer drie. Want dat was volgens mij net een van de laatste. Vier. Vijf. Zes. Zeven. Acht. Negen. Tien. Elf. Twaalf. Dertien. Viertien. | Vult de ranking uit zichzelf correct in  [Independently completes the ranking correctly] |  |  |
| 3 |  | scrolt horizontaal Ja, dat is 1 [vult 1 correct in]. [scrolt horizontaal] 2 [vult 2 correct in]. Hmm, [scrolt horizontaal] 3 [vult 3 correct in]. 4, 5 [vult 4 en 5 correct in]. Het is nog best wel een klusje eigenlijk [vult 6 correct in]. Zeker als het een hele grote.. eh [scrolt horizontaal, vult 7 correct in]], een heel groot cluster is. Nouja hoewel [vult 8 correct in], aan de andere kant, ehm, hangt het natuurlijk ook af van hoeveel modules je kan prioriteren [scrolt horizontaal]. En dan valt het wel mee hoe groot dit klusje is. 9 [vult 9 correct in, scrolt horizontaal] Tien. [vult 10 correct in, vult 11 correct in, scrolt horizontaal]. 12 [vult 12 correct in, scrolt horizontaal, vult 13 in, scrolt horizontaal, vult 14 correct in]. 15… [scrolt horizontaal] hier, 15 [vult 15 correct in]. | Voert de ranking correct uit  [Performs the ranking correctly] |  |  |
| 3 |  | Dan is dit 1 [vult 1 als rank correct in in de cel boven de winnaar]. Dan willen we natuurlijk het verschil zien tussen deze top-15 en de vorige top-15. Ehm… [scrolt horizontaal, vult 2 correct in, scrolt horizontaal, vult 3 correct in, vult 4 correct in, scrolt horizontaal] Vijf [vult 5 correct in]. | Voert de ranking correct uit  [Performs the ranking correctly] |  |  |
| 3 |  | [scrolt horizontaal, vult 2 correct in] Ho, ik klik iets te ver door [klikt verkeer op de scoll bar]. Ben de tel kwijt [lacht]. Drie… Volgens mij was het drie. Wat gebeurde er nou? Drie [vult 3 correct in, scrolt horizontaal], vier [vult 4 correct in], vijf [vult 5 correct in, scrolt horizontaal, vult 6 zin, scrolt horizontaal]… Zeven [vult 7 correct in, scrolt horizontaal], acht [vult 8 correct in], negen [vult 9 correct in], tien [vult 10 correct in, scrolt horizontaal], elf [vult 11 correct in], twaalf [vult 12 correct in, scrolt horizontaal, vult 13 correct in]… veertien [vult 14 correct in], en de laatste. Vijftien [vult 15 correct in]. | Voert de ranking correct uit  [Carries out the ranking correctly] |  |  |
| 4 |  | [vult 4 correct in op rij 2, vult 5 correct in op rij 2, vult 6 correct in op rij 2, vult 7 correct in op rij 2, vult 8 correct in op rij 2, vult 9 correct in op rij 2, vult 10 correct in op rij 2, vult 11 correct in op rij 2, vult 12 correct in op rij 2, vult 13 correct in op rij 2, vult 14 correct in op rij 2] Oke [vult 15 correct in op rij 2], 15, he? | Correcte uitvoering van de handmatige ranking  [Carries out the manual ranking correctly] |  |  |
| 4 | [I]: Je mag eerst een top-15 maken, door de rank als cijfer boven de winnaar te zetten. Dus je ziet, ehm, en dat is even.. Nu ga ik ook eh, even wat anders doen dan in de vorige scenario 1. Nu zie je, zeg maar, ‘winner’ staan.  [P04]: [scrolt horizontaal] Ja?  [I]: Daar oven mag je de rank telkens zetten.  [P04]: [klikt op de cel in rij 2 boven ‘winner’]  [I]: Dus dit is 1.  [P04]: Ohhh! [vult 1 correct in op rij 2]  [I]: En dan gaat ‘ie verspringen en dan de volgende winnaar is 2. Die heeft de tweede rank. | [scrolt horizontaal] Oh, dat gaat dan… Oh! [vult 2 correct in op rij 2, scrolt horizontaal] Oh, dan moet je me maar een keer uitleggen hoe dat dan gaat, maar goed. Eh! [lacht, vult 3 correct in op rij 2]  (…)  Vier [vult 4 correct in op rij 2], dat gaat iets sneller dan toen net [vult 5 correct in op rij 2]. Zes [vult 6 correct in op rij 2, scrolt horizontaal]. 7 [vult 7 correct in op rij 2], 8 [vult 8 correct in op rij 2], 9 [vult 9 correct in op rij 2], 10 [vult 10 correct in op rij 2, scrolt horizontaal], ehmm, 11 [vult 11 correct in op rij 2, scrolt horizontaal]. He, 12 [vult 12 correct in op rij 2, scrolt horizontaal]. 13 [vult 13 correct in op rij 2, scrolt horizontaal]. 14 [vult 14 correct in op rij 2] vijftien [vult 15 correct in op rij 2, vult 16 correct in op rij 2]. | Manier van ranken lukt na aanwijzing  [Method of ranking is clear after additional instruction] | Some additional information may still need to be provided about where and how to rank in the tool, whereafter ranking can be carried out correctly. | We will automate the ranking process in the next iteration of the tool and provide guidance in an accompanying document. |
| 4 |  | [vult 1 correct in op rij 2, scrolt horizontaal, vult 2 correct in op rij 2, vult 3 correct in op rij 2, scrolt horizontaal, vult 4 correct in op rij 2, scrolt horizontaal, vult 5 correct in op rij 2, vult 6 correct in op rij 2, vult 7 correct in op rij 2, scrolt horizontaal, vult 8 correct in op rij 2] | Ranken lukt meteen na een eerdere aanwijzing tijdens scenario 2  [Carrying out the ranking correctly after prior intructions in scenario 2] |  |  |
| 2 |  | [drukt op enter zodat de selectie verschiet naar de eerstvolgende onvergrendelde cel] Hey.. Waarom verschiet ‘ie nou de hele tijd naar beneden? Huu. | Verspringen van selectie naar onbeveiligde cellen (door enter)  [Pressing enter causes the selected cel lto jump tot he next unprotected cell] | Securing cells in het worksheet allow only some cells to be selected. Pressing enter will switch to the next available cell. | We keep the option to secure cells to guide the user through the available cells. We will add additional information to an accomanying document how to disable secured cells. |
| 2 |  | Mijn gedachten hierbij is dat het wel, best wel veel handmatig veel werk is wat je volgens mij ook automatisch door Excel zou moeten kunnen laten doen [scrollt horizontaal]. Als het toch al zo mooi automatisch kan. | Wens om de ranking te automatiseren  [Wish to automate the ranking] | Wish to automate the method of ranking because manually ranking is considered to be a lot of work. | We will automate the ranking process in the next iteration of the tool. |
| 4 |  | En kan ik dan niet dit [selecteert de cel voor penalty method B29 in de tool parameters] dan op 2 aanpassen, en dat ‘ie dan..  (…)  [scrolt horizontaal] Maar als ik dan, oh dan.. Dan zijn die, dan is dit [selecteert een aantal cellen in rij 2 met de ranks van items] natuurlijk niet meer kloppend, want dit weegt waarschijnlijk mee in hoe ‘ie dan de winnaar beoordeelt. Dus dan moet je dat eerst leeg maken [selecteert een reeks cellen met ranks in rij 2 en verwijdert de inhoud], denk ik. | Verwachting dat de eerder ingevulde ranking direct wijzigt als er een andere penalty methode wordt toegepast  [Expectation that the filled out ranking immediately changes when selecting a different penalty method] | Some might expect that the tool automatically adjusts all prior filled out rankings when tool parameters change. However, this is currently not the case. | It is dificult to provide real-time feedback when tool parameters are changed. We will develop a butten to clear all rankings at once and provide additional guidance about changing tool parameters when a ranking was performed. |

**S3 Table 5. Part 5: Semi-structured interview**

| **P** | **Participant text** | **Label/observation** | **Interpretation** | **Actions to improve** |
| --- | --- | --- | --- | --- |
| 1 | Maar op zich is het… goed dat als je daar dus een penalty voor toepast dat de ranking ook wel veranderd. Dat er dus wel rekening mee wordt gehouden dat mensen ook echt volgens hun eigen belangen stemmen. Zeker als er.. Volgens mij was er 1 vereniging waar er 5 mensen aan meededen, ja, die modules worden dan automatisch hoog gezet. Ik denk dat dit een mooie manier is om mee te nemen. | Ziet nut van optie voor penalty, disproportionele representatie  [Sees use for a panalty: disproportionate representation] | There are some considerations about whether and when to use (dis)proprotional representation methods | We replace the current adjusted weighting methods (based on the number of voters) with another adjusted weighting method. There are pros and cons to both proportional en disproportional representation, depending on the context. We aim to keep both types of weighting methods in the tool. |
| 1 | Ik denk dat dat wel eerlijk is, want volgens mij was van de <*wetenschappelijke vereniging*> waren er 5 die gestemd hadden als ik het mij goed.. ik mij niet vergis. Dus ik denk dat het beter is dat je dan dus ook andere verenigingen modules toebedeeld. | Proportionele representatie lijkt niet altijd per se wenselijk  [Proportional representation does not always seem tob e desirable] |  |  |
| 2 | Nou ik, ja, ik, i-ik vraag mij eigenlijk een beetje af in hoeverre dat zeg maar wenselijk is. Ehm…, wat ik mij wel kan voorstellen, dat je inderdaad situaties hebt waarin je… ehm, met je belangrijke partners, eh.. in dit geval is de <*wetenschappelijke vereniging*> waarschijnlijk de hoofd WV en is het met je partners waarschijnlijk belangrijk dat je samen met je partners, zeg maar, samen gaat prioriteren maar tegelijkertijd… eh, als ik denk aan mijn eigen cluster benigne gynaecologie, dan zijn zeg maar… ja dan kan ik mij ook voorstellen dat het voornamelijk alle gynaecologische modules zijn die geprioriteerd worden, omdat dat, ja eh.. het doel van het cluster denk ik ook dient. Dus ik weet niet zo goed of dit… eh, per se de oplossing is om tot een betere prioritering te komen. | Afweging proportional vs disproportional representation  [Consideration for proportional vs disproportional representation] |  |  |
| 2 | Ehm, het heeft ook het voordeel dat je, bijvoorbeeld, voor een kleine partij als de <*wetenschappelijke vereniging*>, die vaak maar met 1 persoon in zo’n cluster zal zitten, dat je wel je stem, eh, kan inbrengen. Zeker als je in zo’n groot chirurgisch cluster zit. Ehm.. Maar ja… Nah ik weet ook niet zo goed wat je wel als prioriteringsmethode… Misschien moet je.. Ja, afspraken maken afhankelijk van het gewogen… He, hoeveelheid… | Afweging proportional vs disproportional representation  [Consideration for proportional vs disproportional representation] |  |  |
| 2 | Wat ik gewoon nu nog niet zo goed zie in dit voorbeeld is, het lijkt nu zeg maar alsof er soort van heel mooi divers palet, zeg maar, in het tweede scenario eh, ge..geranked is. Maar als dat helemaal niet reflecteert dat, zeg maar, 95% van het cluster puur <*wetenschappelijke vereniging*> modules zijn, dan ..voelt het meer alsof je een soort van dansje doet om alle kleine partijen tegemoet te komen dan dat je.. recht doet aan de inhoud van je cluster. | Afweging proportional vs disproportional representation  [Consideration for proportional vs disproportional representation] |  |  |
| 3 | Ehm, en eh, de groepsgrootte van een aantal afgevaardigden vanuit de WV zou er niet al te veel invloed op moeten hebben. En het lijkt erop dat dat als je dat beter spreidt, dat als je wel een penalty toevoegt, dat dan in ieder geval meer WVen hun zin krijgen. En tegelijkertijd zijn er natuurlijk veel argumenten te bedenken waarom je module prioriteert ja of nee, behalve alleen je WV belang. | Afweging proportional vs disproportional representation  [Consideration for proportional vs disproportional representation] |  |  |
| 4 | ..dat is natuurlijk meer bij die eerste. En dus dan denk ik dat dat dus wel beter verdeeld is hier, want dan is dit, hier zie, bij die tweede, bij die zeg maar die eerste van die ranking, dus de tweede, daar zijn er maar twee modules van de <*wetenschappelijke vereniging*> geprioriteerd. En bij die laatste ook twee, drie, iets meer, vier. Ja, dus dat is wel beter verdeeld dan bij de, zeg maar, de echt, wat we helemaal als eerste deden.  (…)  In verhouding? Ja dan vind ik dat wel terecht, denk ik. Ehm, omdat anders wel is ehm, de meeste stemmen gelden. Aan de andere kant… Ehh, Ja, als er meer mensen van dezelfde vereniging in een werkgroep of, zeg maar, in een clusterwerkgroep zitten, dan wil je denk ik wel voorkomen dat er, dat.. dat is dan de meeste st… dat.. dat dat zeg maar.. dat dat zeg maar, dat uiteindelijk alleen maar de modules, dat de modules van die partij alleen, uiteindelijk alleen geprioriteerd worden. Aan de andere kant wil je dan ook weer niet helemaal dat het, zeg maar, is dat het… stel het is wel een cluster wat wel heel chirurgisch is, net zoals bijvoorbeeld traumatologie bij mij, dat het dan, dat dan, dat het dan zo streng gerekend wordt dat er dan uiteindelijk nog maar heel weinig komen vanuit de <*wetenschappelijke vereniging*> en dan heel veel andere modules. Want, dat het dan ook wel een heel chirurgisch onderwerp is, zeg maar. Dus dat het dan daarom veel chirurgen zijn | Afweging proportional vs disproportional representation  [Consideration for proportional vs disproportional representation] |  |  |
| 1 | Het is wel een beetje een black-box nu voor mij, van nou.. we vullen 2 en 4 in en er komt wat anders uit. | Tool geeft geen feedback over de berekening van het individuele gewicht  [Tool does not provide feedback in the calculation of individual weights] | The tool does not provide feedback when changing tool parameters or when calculating individual weights | Since most formulas run in the background, it is dificult to provide real-time feedback. We will provide additional information in an accompanying document |
| 1 | Ja ik zou zelf wat meer achtergrond willen in hoe het wordt berekend, maar dat is misschien als onderzoeker dat je dat meer wilt weten van: hoe dan? Ik weet niet, staat het ergens in de tool ofniet? | Tool geeft geen feedback over de berekening van het individuele gewicht  [Tool does not provide feedback in the calculation of individual weights] |  |  |
| 1 | Ja, want jij zei bij die penalty aggression: vul daar maar 4 in. Maar ik heb nergens kunnen zien waar tussen je kon kiezen ofzo. En dat is misschien fijn als daar net nog als bij dat penalty method iets achter staat. Wanneer je wat moet invullen. | Tool geeft geen feedback over het gebruik van de penalty methode/ aggression  [The tool does not provide feedback about the use of the penalty method and aggression] |  |  |
| 4 | Ja dus dan, ja maar de verschillende was dat dan nummer 2 en 3? Ja ik, vind het dan heel lastig om te zien, zeg maar wat dan nummer drie doet. En ik heb voor mijzelf dan, denk ik, nu niet echt, dat het best wel snel ging voor mij dat ik dacht van: het beeld van hoe dan het verschil tussen 2 en 3 tot stand is gekomen om dan een goede mening te formuleren wat ik dan van 2 vond. | Tool geeft niet direct feedback over penalty methode  [Tool does not provide feedback about the penalty method] |  |  |
| 1 | Je vult alles in en er komt vanzelf een winnaar uit. Ik dacht eerst van: oh dat moet je nu zelf gaan berekenen, maar de winnaar had ik nog even niet gespot. Maar op zich, dat groene vlak valt heel goed op waardoor je heel snel de ranking kan toepassen. Dat werkt wel heel prettig, ja. | Automatisering in de tool wordt op prijs gesteld  [Automation in the tool is being appreciated] | Automation within the tool is being appreciated | - |
| 1 | Die ranking, ja die wordt ook automatisch berekend als je die score hebt ingevuld, dus dat werk heel prettig. | Automatisering van detectie winnaar wordt op prijs gesteld  [automatic detection of the winner is being valued] |  |  |
| 4 | En ik vond het op zich wel goed dat je dan, kijk normaal heb je natuurlijk nog instructie bij hoe zo’n tool werkt, maar dat je dan die winner, dat je dan zo die, die eh, ranking zelf in kan vullen, maar dat je daar zelf verder niks voor hoeft te berekenen, dat ‘ie dat dan automatisch volgt. Ja… | Automatische identificatie van de winnaar is handig  [Automatic identification of the winner is helpful] |  |  |
| 1 | Ja het werkt wel heel prettig. Ja. Het zou mooi zijn als je scherm, als je dit aan het invullen bent, want zeker als er straks 100 zijn ben je best wel aan het scrollen, dat ‘ie automatisch naar het stukje gaat waar dan winnaar staat. Dat.. Dat maakt ‘m nog makkelijker om te gebruiken, maar ik weet niet of dat mogelijk is in Excel. Dat soort dingen. | Wens om de ranking te automatiseren, anders veel scrollen  [Wish to automate the ranking method, otherwise it is a lot of scrolling] | There seems to be a wish to automate the ranking method in order to avoid acrolling through the worksheet] | We will automate the ranking process in the next iteration of the tool. |
| 2 | Dat handmatig de ranking invullen, dat zou ik automatisch door Excel, zeg maar, laten doen. Dat je dat naar je tabblad laat, eh… invullen zeg maar. Dat lijkt mij eigenlijk wel makkelijk, want dat zit je niet de hele tijd te tutten, en dan kan je niet een fout maken dat je net een getal mist, ofzo. | Wens om ranking te automatiseren  [Wish to automate the ranking method] |  |  |
| 2 | Ja, maar dan zou je wel, eh.. een filter er in kunnen zetten en gewoon aanklikken: sorteer van hoog naar laag, bijvoorbeeld. En dan doet ‘ie het automatisch, denk ik, voor je dat je dan.. ehm, je hoogst scorende module bovenaan krijgt. | Wens om ranking te automatiseren  [Wish to automate the ranking method] |  |  |
| 3 | Ja, dat zou misschien ook een goede toevoeging zijn. Dan moet je dus wel goed kunnen aangeven tot waar je wilt ranken. Eh, ja… tegelijkertijd houd je misschien iets meer feeling er mee als je het handmatig doet? Ja, ik weet niet of dat heel veel zou toevoegen eigenlijk. Je ziet natuurlijk ook de volgorde vanzelf. | Twijfel of automatisch ranken zorg voor verlies van gevoel voor data  [Doubt whether automated ranking resukts is loss of felling with the data] | There might be some worries that automated ranking may cause a loss of feeling with the data | We will automate the ranking process in the next iteration of the tool. This will be done semi-automatically (by pressing a button to assign the next rank) in order to be able to trace the ranking process. |
| 2 | Maar als je natuurlijk heel veel modules hebt, zoals ik bij benigne gynaecologie hebben wij 186 modules, uit mijn hoofd. Ja, dat werkt natuurlijk niet als je die ranking [lacht] moet gaan zoeken, zeg maar, eh, eh, van waar nou de winnaar zit. Dus dan is het misschien toch handiger om die modules onder elkaar te listen. Om het overzicht te krijgen, zit ik mij zo gauw te bedenken. | Overzicht om handmatig te ranken bij veel modules ontbreekt  [There is a lack of overview when assigning ranks manually if there are lots of items] | Overview is lacking when manually assigning ranks in the tool when there are lots of items | We will automate the ranking process in the next iteration of the tool. This will reduce the need for horizontal scrolling in the worksheet (except for ties). |
| 2 | Nee, nouja ik denk, nee. Behalve dat ik dus denk dat de uitdaging vooral ligt van hoe je dat.. hoe je dit nu overzichtelijk houdt, als je dus heel veel modules hebt en dat je dan handmatig die ranking moet doen. Ik dat dat gewoon niet… ik denk dat dat niet werkt. | Handmatig ranken lijkt onoverzichtelijk indien er veel items in de tool staan  [Ranking manually is inconvenient when there are lots of items] |  |  |
| 3 | Ehm, het maken van die ranking, dat is nog wel eventjes een soort uitzoekwerkje. | Manier van ranken is uitzoeken  [Manner of ranking is something to figure out] |  |  |
| 4 | Want ik heb straks eh, volgens mij zijn het er 2… ik noem 200 ofzo, maar dat zijn natuurlijk enorm veel kolommen. Ehm, en ook als je dan zeg maar zelf de, die, die ranking moet doen. Dan moet je natuurlijk wel de hele tijd scrollen van links naar rechts. | Veel horizontaal scrollen bij handmatige ranking  [Lots of horizontal scrolling when ranking manually] |  |  |
| 1 | Nou, die verenigingen hebben we volgens mij niks mee gedaan. Alleen die voters zat ik een beetje van, want dan ben je aan het zoeken. Ehm, maar ik weet niet of dat makkelijker kan op een andere manier. Want nu zijn er heel veel partijen die je eigenlijk niet nodig hebt. Misschien zou je nog.. Want hier hebben we natuurlijk… Je ziet natuurlijk mijn scherm niet meer. Eh, het eerste tabblad, dat RRV, daar heb je nu organisaties ingevuld. Dat zou ergens makkelijk zijn als ‘ie dat automatisch dan ook bij het aantal voters optelt. Dan hoef je het niet meer zelf in te vullen.  (…)  Maar dat maakt in ieder geval het tabblad voters nog weer gebruiksvriendelijker | Automatisering van invullen aantal voters zou wenselijk zijn  [Automation of counting the number of participating voters would be appreciated] | Thereis a wish to automate the counting of the number of participating voters | We have decided that we will use a different weighting method independent of the number of voters per organization. Therefore, the number of voters per organization does not have to be filled out in the next iteration of the tool. |
| 2 | Maar ik weet niet of dat onder dit kopje valt: maar bijvoorbeeld bij het kopiëren van hoeveel mensen er in een vereniging zitten uit jouw cluster, zou je het misschien makkelijker kunnen maken door ofwel, ehm, de lijst waaruit je kunt kopiëren dat dat eigenlijk altijd een standaard lijstje is die je er gewoon overheen kunt plakken. Ehm, of iets anders dat je niet… nou goed, dat je zo min mogelijk typfouten, zeg maar, kan maken bij het overtypen. Dat is misschien nog handig. | Wens om het invullen van het aantal voters te automatiseren  [Wish to automate the counting of the participating voters] |  |  |
| 3 | Eh, ja ik vroeg mij af of het niet in te bouwen is dat het aantal voters per vereniging, dat dat ook automatisch berekend wordt vanuit het RRV-tabblad. Want je geeft… Ja misschien is dat wel lastiger te programmeren, maar je geeft aan dat er een naam achter een WV komt, dat zou je dan toch wel moeten kunnen optellen. Dat zou misschien het gebruiksgemak nog wel vergroten, in plaats van dat je dat nog handmatig invoegt. | Wens om het invullen van het aantal voters te automatiseren  [Wish to automate the count of participating voters] |  |  |
| 1 | Zeker als er dan straks, eh, als je dus die penalty toepast en dat het dan in een keer anders wordt. Ik kan mij voorstellen dat de <*wetenschappelijke vereniging*> dan opeens denkt: we hadden eerst negen modules en nu nog maar 5. Dat je dan kan uitleggen hoe dat gedaan is. Ik weet niet of mensen gelijk genoegen nemen met: ik heb een penalty toegepast en daarom zijn er nu minder modules. | Achtergrond-informatie over penalty nodig om keuzes en uitkomsten te onderbouwen  [Background information about penalty methods is needed to provide an argument for choices and outcomes] | There is a lack of background information about how the tool works and there is a lack of guidance which method and aggression to use. | We will provide additional information in an accompanying document about the workings of the tool, including the penalty methods and penalty aggression. |
| 1 | In principe wel, alleen ik zou dan de achtergrond van die penalty methode willen weten. | Achtergrond-informatie over penalty methode wenselijk  [Background information about the penalty method seems desirable] |  |  |
| 2 | Ja, ehm… Ik moet eerlijk zeggen dat ik het eigenlijk nog niet zo goed volg. Ik zit… Ik heb het Word-bestand er nog even bij geopend.  (…)  Maar het gaat mij een beetje te snel om te zien waar precies de verschillen in zitten, maar ik zie inderdaad wel er v.. dat er gewoon andere modules eh,tussen de twee dingen zitten, maar ik snap niet , ik snap nog niet wat ik gedaan heb met die penalty. | Werking penalty methode nog onduidelijk  [It is unclear how the penalty method works] |  |  |
| 2 | Ik denk.. Het is een mooie tool. Ehm, ik snap nog niet helemaal hoe die werkt. | Werking/ achtergrond tool onduidelijk  [Mechanism / background of the tool is unclear] |  |  |
| 2 | Misschien het laatste wat me als laatste nog te binnen schiet is dat op die RRV, eh, dat eerste tabblad, daar staat natuurlijk super veel data, ehm… waar je misschien wel helemaal niks mee doet en dan… weet ik niet zo goed of dat alleen maar ruis veroorzaakt. Misschien als je als adviseur uitgelegd krijgt wat je er mee kan dan is het handig. | Achtergrond-informatie is wenselijk om de tool te begrijpen  [Background information to understand the tool is desirable] |  |  |
| 2 | Uhm, ja je moet als adviseur een goede instructie van jou of van iemand anders die weet hoe het werkt. | Achtergrond-informatie of instructie over de werking van de tool lijkt wenselijk  [Background information about the tool’s working seems desirable] |  |  |
| 2 | je moet wel heel goed aan een werkgroep kunnen uitleggen waarom je een penalty van een bepaalde zwaarte of iets, zeg maar, toe..kent. Ehm… En goed je mannetje kunnen staan, zeg maar, om daar een discussie over aan te gaan. Want.. ik kan nu, als ik aan de werkgroep moet uitleggen waarom ik die penalty 1 score heb gegeven, kan ik dat niet, zeg maar, makkelijk aan ze uitleggen. Dus dat is denk ik wel een vereiste, dat je goed ehm… beslagen ten ijs komt en ik denk, als laatste, dat je aan de werkgroep alleen het simpele overzicht moet laten zien. Ehm. En dat we als adviseurs daar allemaal uniform in geïnstrueerd moeten worden van: dat je niet nog de situatie zou krijgen dat in het ene cluster penalty 1 wordt gekozen en in het andere cluster niet d.. zo’n penalty, terwijl je dezelfde situatie hebt. | Achtergrond-informatie over de werking lijkt wenselijk om beslagen ten ijs te komen bij de vergateringen met deelnemers  [Background information about the tool’s working seems desirable to come prepared to participant-meetings] |  |  |
| 4 | Alleen dit was mij nog niet helemaal duidelijk, oh dat kun je natuurlijk nu niet zien, bij die penalty, waarom je dan voor 4 kiest, maar goed dat mag je misschien nu ook nog niet zeggen. Ehm, maar dat is misschien meer achtergrondinformatie dan dat je dat echt in zo’n formulier moet zetten. | Keuze voor welke mate aggression onduidelijk  [Choice for which size of aggression is unclear] |  |  |
| 4 | Ehh… ik vond eigenlijk de sessie uiteindelijk best wel kort om nu goed in te kunnen schatten hoe het dan echt werkt en of ik dan echt dingen mis, ofzo. Snap je wat ik bedoel?  (…)  Ja, misschien dan toch iets meer van die achtergrondinformatie over die, die, dat penalty, die methode, ofzo? | Geen achtergrondinformatie gegeven  [No background information provided] |  |  |
| 4 | Ehm, alleen dat je dat hier bij de penalty, dat je dan zelf iets in moest vullen dat wist ik dan ook niet, maar goed daar krijg je dan misschien ook instructie voor. | Achtergrond informatie over penalty (aggression) ontbreekt nog  [Background information about the the penalty (aggression) is still missing] |  |  |
| 4 | Ja wat ook natuurlijk, eigenlijk wat we al soort van benoemd hebben, dat iets meer achtergrond wilde hebben over die, dat penalty eh, methode, die drie methodes die daar staan, twee? | Meer achtergrond informatie over penalty methode  [More background information about the penalty method] |  |  |
| 3 | Ehm, sowiezo moet je natuurlijk weten wat we precies bedoelen met prioriteren. Ehm, en ehm… Je moet natuurlijk ook.. je moet eigenlijk ook de tool een keer hebben gezien voordat je gaat prioriteren.  (…)  Dus eigenlijk is het wel handig dat je een keer het gezien hebt of een keer er in gespeeld hebt, zeg maar, voordat je daadwerkelijk met het prioriteren aan de slag gaat, van start gaat.  (…)  Ik denk dat de allerbeste methode is dat je er zelf een keertje wat mee hebt gedaan. Ehm, maar een demonstratie zou wel helpen. | Enige achtergrond lijkt noodzakelijk voordat je de tool daadwerkelijk in gaat zetten  [Some background seems necessary when actually using the tool] |  |  |
| 1 | Ik denk een handleiding, inderdaad.  (…)  Dat hoeft niet in de tool om het onnodig ingewikkeld te maken. | Achtergrond-informatie niet in de tool maar in een handleiding  [Provide background information in a manual and not in the tool] | Background information can be provided in a manual, not necessarily within the tool itself | We will focus on providing additional information within an accompanying document, rather than extending the guiding information within the tool. |
| 1 | Ik denk dat het goed is en ook de kracht is van de tool nu en ik zou dit soort informatie er dan inderdaad niet bij zetten. | Achtergrond-informatie niet in de tool maar in een handleiding  [Provide backgroundinformation in a manual and not in the tool] |  |  |
| 1 | Ja… dan zou je bijna met tekstballonnetjes moeten werken met: stap 1 vul hier wat in, ofzo. Maar ik weet niet of dat mogelijk is en… in principe als je gewoon uitleg er bij hebt is het ook niet nodig. | Achtergrond-informatie in een apart document lijkt voldoende  [Background information in a manual may be sufficient] |  |  |
| 1 | Het lijkt in het begin als je ‘m ziet heel overweldigend, met alle valkjes en dingetjes. Maar als je ‘m eenmaal gaat gebruiken valt het reuze mee. | De tool lijkt relatief gebruiksvriendelijk na de eerste indruk en gebruik  [The tool seems relatively user-friendly after the first impression and use] | It seems dificult to grasp the tool at first, but seems to get more structured/user-friendly after it is used and some background information is received | We understand that the first impression of the tool and its structure is complex. We hope by providing additional background information about the structure and workings of the tool that the tool is immediately usable. |
| 2 | ik denk, zeg maar, als je als adviseur snapt hoe die werkt dat het dan, dat het dan een overzichtlijke en goed gestructureerde tool is. Maar in eerste instantie is het denk ik best wel complex om eh.. om te vatten. | Overzichtelijk en gestructureerd als de gebruiker de tool kent  [Clear and structured tool once the user understands the tool] |  |  |
| 1 | Ik vind hem best overzichtelijk, maar het is wel dat je ‘m even moet gebruiken voor je door hebt hoe ‘ie werkt. Het is niet dat je in een oogopslag denk van: oke, dit moet ik nu doen. | De tool lijkt relatief overzichtelijk, maar niet in eerste oogopslag  [The tool seems relatively clear, but not in the first impression] |  |  |
| 1 | Nee, want ik het begin ging ik de mist in met die ehh. Ranking. Dat ik hier de scores ging invullen. Later, als je dat zegt: ohja hier staat scores. Dan denk ik ja, dan is het ook dom dat ik het daar invul, maar… Automatisch begint je ook bovenaan in de tool en niet onderaan. | Structuur van de tool is niet meteen helder  [Structure of the tool is not immedialtely clear] |  |  |
| 3 | Ja ik vind het best logisch op elkaar aangrijpen. Ehm, misschien komt dat wel omdat ik een beetje weet wat de bedoeling is van de tool en waar het heen moet. Maar eh ik vond het best wel intuïtief werken, eigenlijk. | Gebruik van tool lijkt intuitief met enige achtergrond  [Use of the tool seems intuitive when provided some background] |  |  |
| 4 | Ja, nadat je mij zeg maar, nou, een paar keer geholpen had werd het duidelijk. Ehm, maar goed, we hebben het natuurlijk, over die scores hadden we het natuurlijk al een keer gehad he, dat ik, dat het voor mij niet helemaal overzichtelijk was, dat je, hoe je dan eh… | Structuur van de duidelijker na enkele aanwijzingen  [Structure of the tool is more clear after some instruction] |  |  |
| 1 | ja er zijn best wel beperkte mogelijkheden om te klikken en dingen in te vullen, dat werkt wel prettig. En… Ja het is fijn dat er dus dingen zwart zijn, dingen grijs. Dat je gewoon weet waar je wel iets mee kan doen en waar je niks mee kan doen. | Lay-out werk sturend  [lay-out provides guidance for its use] | Using colors and restricting cells may guide the user experience within the tool | We will provide some information about resticting cells in worksheets in an accompanying document. |
| 1 | Ja die items erin gaat heel makkelijk, gewoon met knippen en plakken vanuit zo’n ander document. | Eenvoudige input naar de tool  [Easy input into the tool] | Easy way of input and output to/from the tool | - |
| 2 | Ja, weetje, op zich wel prima, als je.. als je gewoon zo kan knippen en plakken uit die voorbeeldscenariotjes is denk ik super fijn dat dat zo werkt. | Manier van input en output is gebruiksvriendelijk.  [Manner of input and output is user-friendly] |  |  |
| 2 | Het formatje, zeg maar, waarin je dat kan aanleveren, eh, kan je zo super makkelijk er zo in plakken, dus dat is ook niet echt moeilijk. | Manier van input naar de tool toe is eenvoudig  [Easy input into the tool] |  |  |
| 3 | Hmm, ik vind sowiezo dat er een paar handige functionaliteiten zijn toegevoegd. Eh, zoals dat je makkelijk kan copy-pasten d’r uit. Eh, dat je ook kan copy-pasten vanuit een Word-bestand of een ander Excel-bestand naar de tool en dat het ook allemaal naar elkaar verwijst. | Manier van input naar en output van de tool is handig  [Input and output is easy] |  |  |
| 4 | nou, ik vond het best wel, op zich wel, redelijk duidelijk, alleen voor mij was het dan niet helemaal duidelijk nog hoe dat je dan de scores dan, zeg maar, er onder moest zetten. Ik dacht natuurlijk dat ‘namen’ dan de namen van de modules was, maar dat is dan de naam van de deelnemer eigenlijk. | Locatie van scores in tool kan onduidelijk zijn  [Location fort he placement of scores in the tool might be unclear] | Score placement in the tool might be difficult te understand at first. | We hope by providing additional background information about the structure and workings of the tool that the tool is immediately usable. |
| 1 | Ik denk dat het goed is om de verenigingen in ieder geval te laten weten dat je, ehm, een soort penalty kan gaan toepassen. Ik weet niet of dat standaard altijd gedaan wordt of dat dat alleen wordt gedaan, zoals je zei, omdat er dan volgens belangen van de eigen vereniging wordt gestemd. Ik weet niet of dat makkelijk inzichtelijk is of dat altijd zo gebeurt. En.. Misschien is het goed om dan in ieder geval mensen van te voren op de hoogte te stellen van: mocht.. mochten ze dat doen, onbewust of bewust, dat we daar voor corrigeren. Aan de andere kant, misschien als je dat van tevoren al zegt gaan ze juist volgens de belangen stemmen, maar… Ik weet niet of dat erg is. [lacht] | Onduidelijk in hoeverre deelnemers inzicht moeten krijgen in de penalties en werking van de tool  [It is unclear to what extent participant should have insights into the penalties and working of the tool] | Some instructions for participants seem appropriate. | Information about assigning priority scores are desirable. We provide additional information about the score (e.g. maximum score) and its interpretation in an additional document. However, we refrain from providing an example text for participants since we believe priority-setting is context dependent and instructions should be tailored accordingly. |
| 1 | Ik denk dat daar iets van achtergrondinformatie wel handig voor is. Dat ze straks niet verrast worden als ze gestemd hebben, van: ja maar we hebben allemaal vanuit de <*wetenschappelijke vereniging*> op die module gestemd, bij wijze van spreken ze zijn allemaal bij elkaar gaan zitten en hebben het samengedaan, en ze komen er niet uit? Ik denk dat dat wel een beetje voor onbegrip zorgt, als je dat niet uitlegt. | Enige achtergrond-informatie voor deelnemers lijkt wenselijk  [Some background information for participants seems desirable] |  |  |
| 2 | Ehm, nou ik denk dat ‘ie heel complex is om aan werkgroepleden zonder uitleg te tonen, zeg maar, en om ‘m te laten zien. Dus misschien dat je, als je dat zou willen doen, dat je dat, dat je daar een soort van nog gemakkelijker dashboard voor maakt of dat je echt alleen maar.. eh, dat kopietje van de ranking laat zien, maar niet het.. die hele eerste tablad, dat is denk ik veel te complex. | De tool zelf is te complex om aan deelnemers te laten zien zonder achtergrond-informatie  [The tool seems too complex to show to participants without providing background information] |  |  |
| 2 | Oke. Ehm, ja als ze hun scores gaan invullen hebben ze gewoon natuurlijk duidelijke instructies nodig en ik denk dat je ze vooraf wel iets moet vertellen over de, eh, penalties die je, zeg maar, kan toedienen. Niet… Ik weet niet zo goed of je daar strategisch stemmen nog vooraf mee kan beïnvloeden op een negatieve manier, maar.. Ik denk wel dat het goed is dat mensen vooraf geïnformeerd, zeg maar, worden over.. nouja gewoon, hoe het werkt en hoe ze het moeten invullen. | Duidelijke instructies voor deelnemers lijkt wenselijk  [Clear instructions for participants seem desirable] |  |  |
| 2 | Maar heel veel van die tekst die dan in het lichtgrij.. sorry, in het lichtgrijs staat, daar vraag ik mij van af van: ja wil je dat eigenlijk wel weten, of eh.. ja… anders denk ik altijd, zeg maar, zo van zo simpel mogelijk is toch zo mooi mogelijk dan. | Inhoud van de instructie/ tekst in de tool is misschien niet relevant  [Contents of the instruction/tekst within the tool might not be relevant] | Contents of the instructions/texts within the tool may not be relavant to all users. | We keep the brief guiding/informational texts within the tool, since it might help some users. |
| 3 | Verder vind ik het ook heel fijn dat er heel veel uitleg bij staat. Dus dat er precies bij staat wat het betekent en wat wat voor consequentie heeft. Dus dat als je bijvoorbeeld voor penalty-methode 2 kiest, dat je dat ook de penalty aggression moet invullen, maar dat als je voor penalty 1 kiest dat het dan niet zo is. Ja, dat vind ik heel verduidelijkend. | Voldoende info gegeven in de tool  [Enough information is provided in the tool] | Contents of the instructions/texts within the tool may be sufficient for some users. | We keep the brief guiding/informational texts within the tool, since it might help some users. |
| 3 | Ik denk zeker dat ‘ie er voor zou kunnen corrigeren ja, ja. En ik denk ook zeker dat je zo een beteren spreiding krijgt wat welke WV belangrijk vindt. Maar ik denk dat het wel altijd nog belangrijk blijft om ook op inhoudelijke gronden te kijken. Dus ook als er zo’n penalty, bijvoorbeeld, over is gegaan, te blijven kijken: zijn de modules die nu zijn geprioriteerd, zijn dat nog steeds de relevante modules op inhoudelijke grond? | Uitkomst van de tool hoeft niet de uitkomst van de priority-setting assessment te zijn  [Output of the tool is not necessarily the same as the outcome of the priority-setting assessment] | The tool’s output may not bet he outcome of the priority-setting assessments and additional steps in the assessment may lead to a definitive outcome. | - |
| 4 | Mean, variantie, standaardafwijking… Nee, ik dank dat dat op zich voor mij wel duidelijk.. dat je zo wel een goed idee krijgt van hoe de res.. of hoe noem je dat? Dat je de spreiding van de scores wel goed kan inschatten zo. | Heterogeniteits-analyse lijkt duidelijk te zijn  [Heterogeneity analysis seems to be cleer | It seems clear how heterogeneity analyses work within the tool. | - |
| 4 | Alleen zat ik nog een beetje mee van: wil je, maar dat weet ik dus niet goed, wil je dan ook nog terug kunnen vinden uit welke richtlijn de module komt? Want dat kan je natuurlijk nu niet zo heel goed terugvinden.  (…)  Ja, nouja, som heb je best wel algemene titels ofzo. Bijvoorbeeld ‘nazorg’, ofzo, in noem maar wat.  (…)  Eh… En dan heb je later een module geprioriteerd, en dan moet je wel, hoe ga ik dan zelf weer terugvinden bij welke richtlijn die hoort? Dan moet je weer soort van naar je basis-bestand en dat daarin bekijken ofzo.  (…)  Ja, ik zit nou, ja richtlijn dubbelepunt modulenaam, dan wordt dit, of tenminste dit, dan wordt de eerste rij in die tabel natuurlijk best wel lang. Ehm, waardoor je dat wel minder goed kan lezen, dus dat zou ik denk ik niet willen, maar hmmm.. ja… ik zou het wel fijn vinden dat je het toch ergens, toch ergens in terug kan vinden.  (…)  Ja, of kan je dan niet bijvoorbeeld hier dat je dan zegt, jaa ik weet niet of daar ruimte voor is, dat je dan op die, in die eerste rij bij die prioriteringstool, dat je dan soort bolletje maakt ofzo, d’r boven, dat je dan kan zien welke richtlijn die modules horen? | Identificatie van de bron van de items ontbreekt  [Identification of the source of the item is missing] | The tool is missing a function to identify the source (title) of an item in the tool. | We will not adjust the tool, but rather add guidance about source identification in a document accompanying the tool. |
| 4 | want als je dan zeg maar die gegeven krijgt, dan ga je als eerste waarschijnlijk de labels invullen. Dan zet je die daar na de RRV tabblad doet. Na die tool, zeg maar, ofzo. En dat je dan zegt van: het aantal stemmers, en dan hoe je het rank, ofzo. | Volgorde van input/ tabbladen inrichten  [Order of tabs] | There might be a desire to change the order of the tabs within the tool according tot he priority-setting process. | Users may change the order of tabs according tot heir own use. |
| 4 | Maar, je moet er wel, denk ik, kijk, je moet er misschien wel best wel nog veel invullen. Maar geen 200, maar… ‘t zijn er misschien wel 50 ofzo. | Zorgen om hoeveelheid in te vullen ranks  [Worries about the number of ranks to assign] | There might be worries that there are a lot of items to assign ranks to. | - |

NB1: Two penalty methods were present in this preliminary version of the tool. In later versions we removed one of the methods, which also expired the need for using the tab ‘voters’ in the tool.

NB2: The remaining penalty-adjusted weighting method and penalty aggression parameter were later renamed to decay-adjusted weighting method and decay aggression, respectively.

NB3: Names of persons and organizations were anonymized and placed between angle brackets.
