# Supplementary material for: Introducing re-weighted range voting in clinical practice guideline prioritization: Development and testing of the re-weighted priority-setting (REPS) tool
Source: PLoS One. 2024 Apr 5;19(4):e0300619. doi: 10.1371/journal.pone.0300619 (PMC10997121; doi:10.1371/journal.pone.0300619)
Supplement: S5 File — This file is a guide to understand and use the REPS-tool in Microsoft Excel. (PDF) [file pone.0300619.s005.pdf]

RE-weighted Priority-Setting (REPS)

A quick-start guide to the REPS-tool

Contents

1. Purpose of the REPS-tool *3*
2. Role in the priority-setting assessment *3*
3. Re-weighted range voting introduction *4*
4. Tool overview *5*
5. Input format *7*
6. Data entry *9*
7. Weighting methods and parameters *11*
8. Assigning ranks *13*
9. Heterogeneity analyses *15*
10. Tool output *16*
11. Adapting the REPS-tool *17*
12. Reference list *17*

Note: the REPS-tool is currently programmed in Microsoft Excel and uses macros. Make sure that Microsoft Excel allows macros on your computer, otherwise the REPS-tool cannot be used. Microsoft Excel might also automatically block macros in Excel-files downloaded from the internet. To use such Excel-files, it is necessary to check the 'unblock' checkbox in the file properties of the Excel-file. To learn more, see: https://learn.microsoft.com/en-gb/DeployOffice/security/internet-macros-blocked

1. Purpose of the REPS-tool

The main purpose of the REPS-tool is to aid in the priority-setting of any item (e.g. clinical practice guideline, systematic review, recommendation, key question, etc.) based on priority scores assigned by participants in the priority-setting assessment by providing a ranked list of items as its output. It aims to be a flexible tool that allows for all different kinds of procedures in the priority-setting assessment before and after using the tool, as long as the input complies to the input format requirement.

Two different types of weighting methods are available [1, 2]:
- The regular Re-weighted Range Voting method
- The decay-adjusted weighting method, based on the regular weighting method, where the decay pattern of individual weights can be adjusted

> Note: the REPS-tool is not a consensus method. It ranks items based on priority scores assigned by a group of participants. Though, consensus might later be achieved using the tool's output by additional steps in the priority-setting assessment when desirable.

2. Role in the priority-setting assessment

The REPS-tool is a function component in a priority-setting assessment. The function needs input according to a specific input format and produces an output in the form of a ranked list. The function's mechanism is re-weighted range voting to assign ranks.

All steps prior to using the function can be considered procedural steps ultimately leading to the correct input format for the function. Organization may wish to use their own set of selected priority indicators, may wish to ask participants to score multiple indicators per item and use the mean score, may wish to use mean scores of groups of stakeholders, and/or any other preferred procedural step. However, it should lead to data satisfying the input format for the REPS-tool.

Once data is entered in the REPS-tool and ranks are assigned, the tool's output can immediately be used as the outcome of the priority-setting assessment (Fig. 1). However, organizations may wish to add one or several procedural steps after the function component. For example, the tool's ranked top 15 output could be used to discuss and form a top 5 priority items as a definitive outcome of the priority-setting assessment.

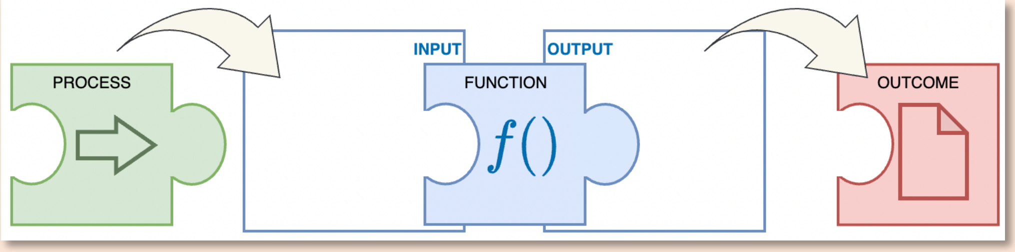

***Figure 1** – A process component leading to the input of the function component. The output of the function component is immediately used as the outcome of the priority-setting assessment, although the latter may not always desirable.*

3. Re-weighted range voting introduction

The REPS-tool uses re-weighted range voting as a mechanism to form a list of items ordered ascendingly by their assigned ranks. The ranks are assigned based on the highest sum score of the items. However, after every assignment of a rank to an item, a weight for each of the participants in the priority-setting assessment is being (re)calculated based on the scores that the participant already assigned to the previously ranked item(s). All participants have a weight starting at '1'. The higher the scores on the previous ranked items for each participant, the lower the individual weight when recalculated. The lower the weight, the less influence on the next item to be ranked as the individual weights are multiplied by the participant's item scores. The re-weighted participants scores are summed to form new item sun scores and the highest item sum score is assigned the subsequent rank.

The calculation of the individual weights is, among others, based on the maximum scale score. Every participant scores items on a scale from 0 to a maximum predefined score (e.g. 5). There is no limit for items to receive the same priority score, so, in theory, a participant could rate all items with the same priority score.

The scale uses a distinctive description. It is not allowed to assign negative priority scores (e.g. -1). The scores can be interpreted as follows:

0 = No priority
1 = Lowest priority possible
…
Maximum scale score = Highest priority possible

The REPS-tool will not adjust the individual weight when a participant assigned a score of 0 or refrained from assigning a score to a winning item.

4. Tool structure

4.1 Introduction to the structure

The REPS-tool consists of three worksheets: RRV (Fig. 2), Labels list (Fig. 3), and Ranking outcome (Fig. 4).

Entry of participant data and priority scores, heterogeneity analyses, and ranking is conducted in the 'RRV' worksheet. It contains six sections: item labels, ranking information, heterogeneity analysis, tool parameters, participants and scores, and ranking buttons.

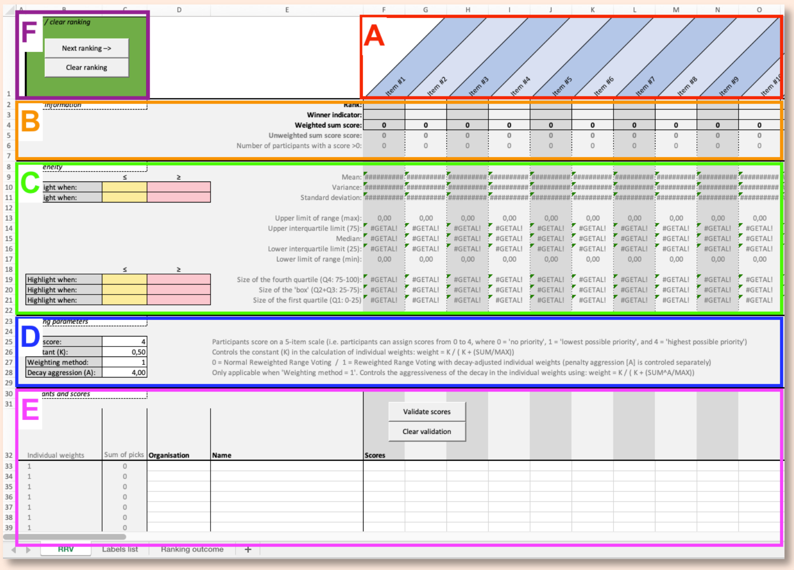

***Figure 2** – Overview of the 'RRV' worksheet in the REPS-tool opened in Microsoft Excel.*

4.2 Section A (Fig. 2A) – Item labels

Item names cannot be filled out in section A in the RRV work sheet. Rather, they are filled out under the Labels list worksheet and projected in section A (Fig. 2A). Item names of items to be prioritized (e.g. CPG names, section titles, recommendations, key questions, etc.) can be placed in the 'Labels list' worksheet (Fig. 3). If a list of item names is available, then the list can be copy/pasted into the Labels list worksheet (Fig. 3).

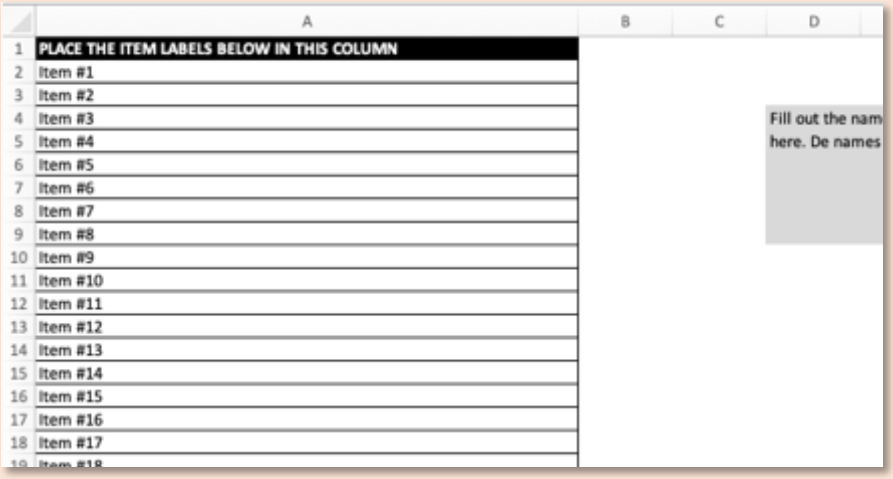

***Figure 3** – Detail of the 'Labels list' worksheet in the REPS-tool opened in Microsoft Excel.*

4.3 Section B (Fig. 2B) – Ranking information
Here, (un)weigted sum scores can be seen per item in each respective column for informative reasons. A winner is automatically detected by the tool, showing a green cell with 'WINNER' in the winning item's column. Although assigning ranks to winning items can be performed semi-automatically, a rank can also be assigned manually (e.g. in case of a tie) by inserting a number in the item's cell on the row named 'Rank' (i.e. immediately above the green cell indicating 'WINNER').

4.4 Section C (Fig. 2C) – Heterogeneity analysis
This section concerns heterogeneity analyses. For each item some measures of central tendency and measures of dispersion are displayed in the item's respective column.

4.5 Section D (Fig. 2D) – Tool parameters
Tool parameters can be adjusted here. Available tool parameters are the constant, the maximum scale score, the weighting method, and the decay aggression (only available for the decay-adjusted weighting method). Changing these parameters will not result in the tool providing any direct feedback, but rather different formulas will operate in the background.

4.5 Section E (Fig. 2E) – Participants and scores
The columns indicating the 'individual weight' and 'sum of picks' are informative for the user and are part of the calculations in the background. The column 'organization' and 'name' are optional, but can be used to identify the participant. Importantly, the field with 'scores' align with the items (columns) and participants (rows) and therefore forms a matrix.

4.6 Section F (Fig. 2F) – Ranking buttons
This section contains two buttons: one to assign a rank to the identified winner and one to clear all assigned rankings. Items with assigned ranks are displayed in the Ranking outcome worksheet (Fig. 4). This list can be copy/pasted to word-processing software and is, effectively, the output of the REPS-tool.

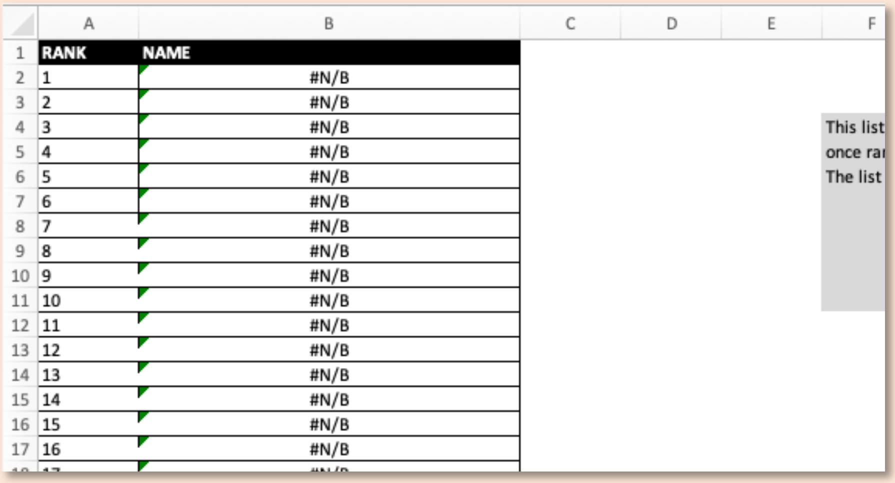

	A	B
1	RANK	NAME
2	1	#N/B
3	2	#N/B
4	3	#N/B
5	4	#N/B
6	5	#N/B
7	6	#N/B
8	7	#N/B
9	8	#N/B
10	9	#N/B
11	10	#N/B
12	11	#N/B
13	12	#N/B
14	13	#N/B
15	14	#N/B
16	15	#N/B
17	16	#N/B

***Figure 4** – Detail of the 'Ranking outcome' worksheet in the REPS-tool opened with Microsoft Excel. Excel shows #N/B because none of the items were currently ranked.*

Note: Worksheets can be protected (whether or not by password) so that cells and formulas are locked in their respective cells when desirable. Restricting cells may also result in users not able to select irrelevant cells guiding their experience in using the REPS-tool.

5. Input format

From Fig. 2A and Fig. 2E it could already be deduced that the columns represent the items to be prioritized and that the rows represent participants. The input format for the tool is thus a matrix with priority scores corresponding to an item and a participant:

***Matrix 1** – Input format for the REPS-tool.*

	Item 1	Item 2	Item 3	Item …
Participant A	Score 1,A	Score 2,A	Score 3,A	Score …,A
Participant B	Score 1,B	Score 2,B	Score 3,B	Score …,B
Participant C	Score 1,C	Score 2,C	Score 3,C	Score …,C
Participant D	Score 1,D	Score 2,D	Score 3,D	Score …,D
Participant E	Score 1,E	Score 2,E	Score 3,E	Score …,E
Participant…	Score 1,…	Score 2,…	Score 3,…	Score …,…

Any priority-setting process may precede, as long as the eventual data used for the REPS-tool complies to the input format in Matrix 1. For example, the scores in Matrix 2 (hypothetical

example) are overall scores the participants assigned based on four priority indicators. However, it is also possible to provide the mean or median of separately scored priority indicators per item

as input for the REPS-tool. Nonetheless, the options in the score scale should always include 0 (i.e. no priority), as a score of 0 will not adjust the individual weight.

***Matrix 2** – Hypothetical example of a dataset (with a maximum scale score of 5) used for input in the REPS-tool to prioritize key questions for development.*

	What is the role of MR imaging in patients with a hepatocellular carcinoma?	What is the role of biopsy in the detection of a hepatocellular carcinoma?	What is the role of preoperative portal vein embolization in patients with a cholangio-carcinoma?	What initial approach should be used in patients with any biliary carcinoma: surgery or systemic therapy?
Oerbekke	4	4	4	5
Gaasterland	2	3	1	4
Van der Laan	0	4	4	2
Hooft	5	2	2	3

Furthermore, when there are significant uneven numbers of delegates from participating stakeholders and this is considered undesirable, the mean or median of the participating delegates of stakeholders can be used as an input in the REPS-tool. Rows then become the stakeholder organizations or (sub-)groups instead of individuals (Matrix 3).

***Matrix 3** – Input format for the REPS-tool when using, for example, the mean of participating delegates per organization.*

	Item 1	Item 2	Item 3	Item …
Organization A	Mean 1,A	Mean 2,A	Mean 3,A	Mean …,A
Organization B	Mean 1,B	Mean 2,B	Mean 3,B	Mean …,B
Organization C	Mean 1,C	Mean 2,C	Mean 3,C	Mean …,C
Organization D	Mean 1,D	Mean 2,D	Mean 3,D	Mean …,D
Organization E	Mean 1,E	Mean 2,E	Mean 3,E	Mean …,E
Organization …	Mean 1,…	Mean 2,…	Mean 3,…	Mean …,…

6. Obtaining priority scores

It is important to recognize that the REPS-tool uses a single score or no score (when participants refrained from assigning a score) per item. Thus, either the cell contains a number or is left blank. There are several ways to obtain a single score, for example:

- Ask to assign an overall priority score for each item
- Ask to assign a priority score on a single priority indicator for each item
- Ask to assign priority scores on multiple priority indicators for each item and average the assigned scores on the priority indicators for each item.

Organizations may have different needs and may operate in different contexts. It might be helpful to select priority indicators which seem relevant to the context and needs. For example,

priority indicators might be dependent of the field the priority-assessment takes place, or for which purpose the item is prioritized for (e.g. update, development de novo, implementation), or even the health care system the organization is operating in.

Scores can be elicited by sending out an (online) survey, for example. In the future, priority-setting tools may be programmed into applications from where the scores might also be elicited.

Note: Literature reviews reporting priority indicators for guidelines are being published in scientific journals. Guideline developing organizations may use such overviews to select priority indicators relevant for their priority-setting assessment. Such literature includes:

- Martínez García L, Pardo-Hernandez H, Superchi C, Niño de Guzman E, Ballesteros M, Ibargoyen Roteta N, McFarlane E, Posso M, Roqué I Figuls M, Rotaeche Del Campo R, Sanabria AJ, Selva A, Solà I, Vernooij RWM, Alonso-Coello P. Methodological systematic review identifies major limitations in prioritization processes for updating. J Clin Epidemiol. 2017 Jun;86:11-24. doi: 10.1016/j.jclinepi.2017.05.008. Epub 2017 May 24. PMID: 28549931.
- El-Harakeh A, Morsi RZ, Fadlallah R, Bou-Karroum L, Lotfi T, Akl EA. Prioritization approaches in the development of health practice guidelines: a systematic review. BMC Health Serv Res. 2019 Oct 15;19(1):692. doi: 10.1186/s12913-019-4567-2. PMID: 31615509; PMCID: PMC6792189.
- El-Harakeh A, Lotfi T, Ahmad A, Morsi RZ, Fadlallah R, Bou-Karroum L, Akl EA. The implementation of prioritization exercises in the development and update of health practice guidelines: A scoping review. PLoS One. 2020 Mar 20;15(3):e0229249. doi: 10.1371/journal.pone.0229249. PMID: 32196520; PMCID: PMC7083273.

7. Data entry

The item names in Matrix 2 are hypothetical examples of questions prioritized to be developed de novo. Item names are automatically displayed in the corresponding columns in the 'RRV' worksheet when the names are placed in the list in the 'Labels list' worksheet (Fig. 5).

Organization, participant names and scores are easily copied and pasted in the tool when using a dataset structured according to the input format. Names and priority scores from Matrix 2 were pasted in the REPS-tool (see Fig. 6). The item names can also be copy/pasted in the 'Labels list' worksheet. It is advisable not to cut/paste or move cells within the tool as underlying cell references may become dislocated, even when the worksheet is protected.

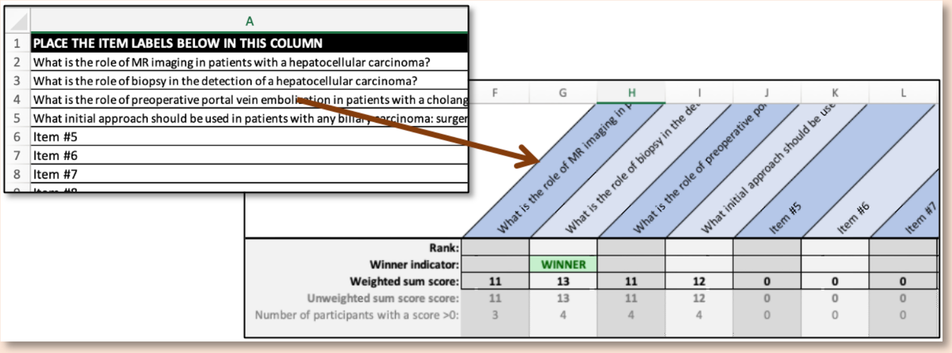

Figure 5 – Item names placed in the 'Labels list' worksheet (Fig. 2) are automatically displayed in the 'RRV' worksheet (Fig. 1A)

Note: Using the same order of participants and items from the dataset in the REPS-tool (i.e. in the 'RRV' and 'Labels list' worksheets) ensures that priority scores can be pasted in the tool without data entry errors. The item names may be horizontally oriented in the dataset. The transpose function Microsoft Excel (e.g. in a new workbook) can be used to create a vertically oriented list following the order of items used in the dataset. The vertical list can be pasted in the 'Labels list' worksheet and item names will appear automatically in the corresponding columns in the 'RRV' worksheet (Fig. 1A).

Note: When items have generic or similar names, consider adding identifiers to their labels. For example, when items are named 'Diagnosis of the primary tumor' and are sections from two different guidelines (e.g. biliary tract cancer and hepatocellular cancer):

- Diagnosis of the primary tumor [BTC]
- Diagnosis of the primary tumor [HHC]

The columns 'Name' and 'Organization' do not necessarily have to contain data to perform the ranking of items in the REPS-tool. However, it is suggested to at least fill in the participant's name (or identifier) when later identification is desirable (e.g. to check for errors in specific rows of data).

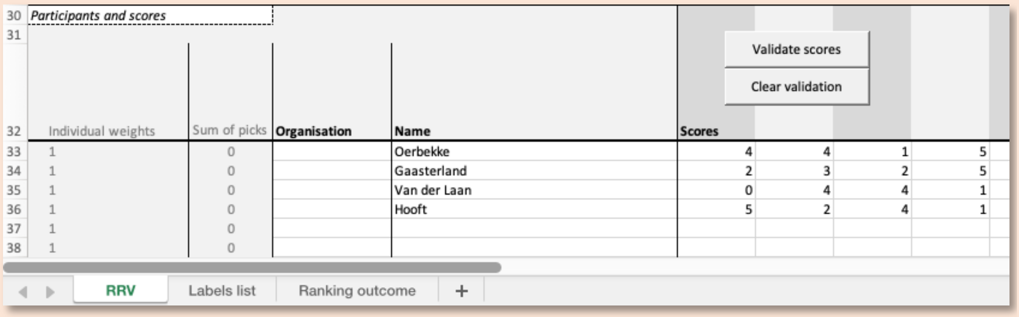

Figure 6 – Names and scores were entered into the REPS-tool ('RRV' worksheet)

In this section the scores can be validated as well. That is, cells with input can be highlighted in color that contain text, values smaller than 0, or values larger than the maximum scale score. Clicking on the button ‘Validate scores’ will highlight the cells (Fig. 7). The button ‘Clear validation’ will remove the highlights.

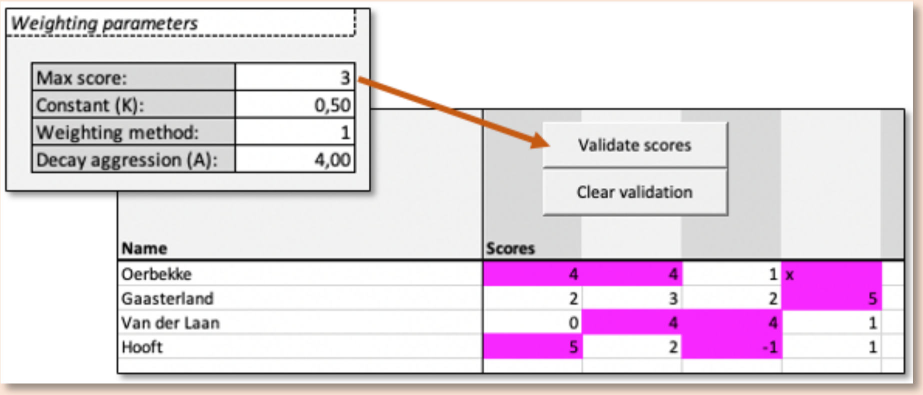

Figure 7 *– With a maximum scale score of ‘3’ the ‘Validate scores’ button will highlight text, any score above 3, and any score below 0. The ‘Clear validation’ button will remove all highlights.*

8. Weighting methods and parameters

The REPS-tool has two weighting methods for individual weights: the regular re-weighted range voting method (Formula 1) and the decay-adjusted weighting method (Formula 2) [1, 2]. The latter formula can be used for disproportionate representation [2].

(1) $$individual\ weight = \frac{constant}{(constant + \frac{sum\ of\ participant's\ scores\ on\ ranked\ winners}{maximum\ scale\ score})}$$

(2) $$individual\ weight = \frac{constant}{(constant + \frac{sum\ of\ assigned\ individual\ scores\ on\ winners^{A}}{maximum\ scale\ score})}$$

The regular re-weighted range voting method will result in a proportional representation (as assigned with priority scores) reflected in the ranking [1, 2]. The decay-adjusted weight (with A>1) results in a more aggressive decay of the individual weights (Fig. 8). A relative aggressive decay (e.g. A=4) causes a steep decline of the individual weight immediately with the first few points assigned to a winning item. This could theoretically leave room for boosting less represented perspectives in the ranking outcome, as participants ‘having their way’ with the first few winners will have a very low individual weight thereafter.

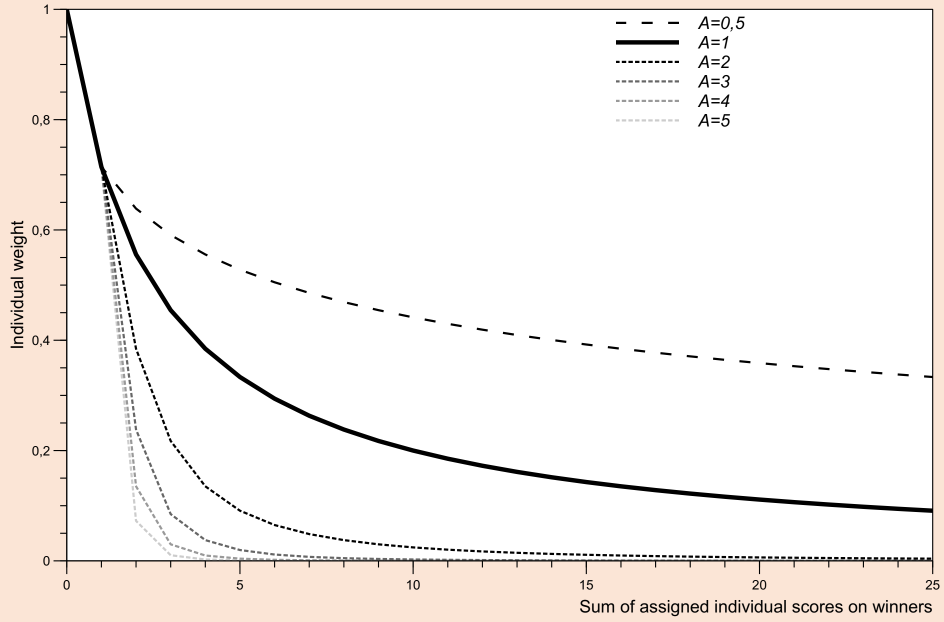

***Figure 8** – A graph showing the decay of individual weights using the decay-adjusted weighting method (Formula 2), a constant of K=0.5, and a maximum scale score of 10. The regular re-weighted range voting method (Formula 1) is identical to A=1.*

The weighting parameters of the individual weights can be selected in the weighting parameters section in the 'RRV' worksheet of the REPS-tool (Fig. 2D, Fig. 9). The value in the cell right to 'Max score:' indicates the maximum scale score. That is, the maximum score a participant is allowed to assign to an item. A brief explanation is provided on the right of the value in the REPS-tool which adapts according to the input value of the maximum scale score. For example, using a maximum scale score of 7, the participants essentially score on an 8-item scale ranging from 0 to 7.

The formulas to calculate individual weights need a constant in both the numerator and denominator in order to result in a weight of '1' when no items were ranked or when no scores were assigned to the previous ranked item(s). The constant can be any positive number [1].

Weighting parameters

Max score:	5
Constant (K):	0,50
Weighting method:	1
Decay aggression (A):	4,00

***Figure 9** – Weighting parameters in the 'RRV' worksheet.*

By changing the value of the cell right to ‘Weighting method:’, the weighting method can be changed. The regular re-weighted range voting method (Formula 1) is used when the value is ‘0’, while the decay-adjusted method (Formula 2) is used when the value is ‘1’. The value next to the cell ‘Decay aggression (A):’ is the value of exponent A in Formula 2.

Note: The tool does not provide immediate feedback when changing parameters. It switches formulas in the background to calculate the individual weights, for example when switching between 0 and 1 as the weighting method.

9. Assigning ranks

The REPS-tool immediately indicates the first ‘winner’ in green when scores are entered (Fig. 10). Make sure not to assign ranks until all data required following the input format is entered to prevent a faulty ranking. In our hypothetical example from Matrix 2, the first winner is “*What is the role of biopsy in the detection of a hepatocellular carcinoma?*” because it had the highest sum score (i.e. 13).

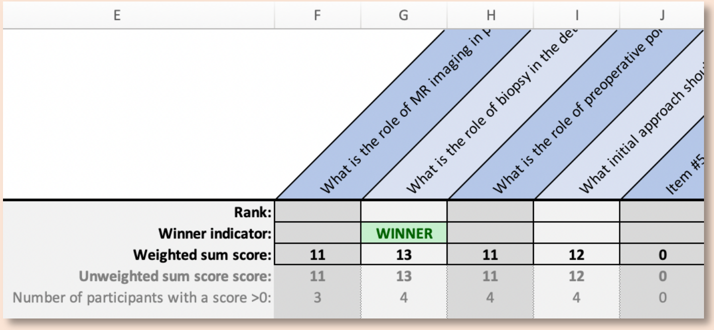

***Figure 10** – Automatic indication of the winner in the ranking information section (also see Fig. 2B). A rank is not yet assigned to the winner at this point.*

By clicking on the ‘Next ranking –>’ button (Fig. 11), the identified ‘winner’ receives its rank and the next ‘winner’ is identified to receive a rank. The button enables a semi-automatic ranking of items (Fig. 12). A message will appear to inform the user there is no item left to rank when the button is clicked and there are no more items left. Ranks can also be manually entered in the row ‘Rank:’ (e.g. fill out ‘1’ in the cell above the cell indicating the winner in Fig. 10). After accepting the value in the cell, the REPS-tool will immediately identify the next ‘winner'.

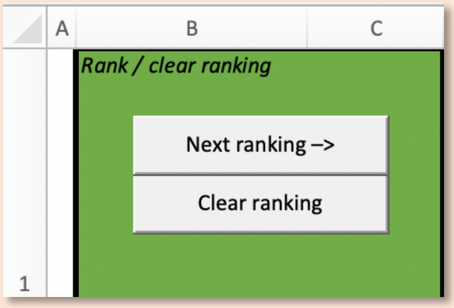

Figure 11 *– Buttons for the semi-automatic assignment of ranks and for clearing all ranks.*

The row ‘Rank:’, containing all of the assigned ranks to the items in the corresponding columns (Fig. 10), can be cleared at once by clicking on the ‘Clear ranking’ button (Fig. 11).

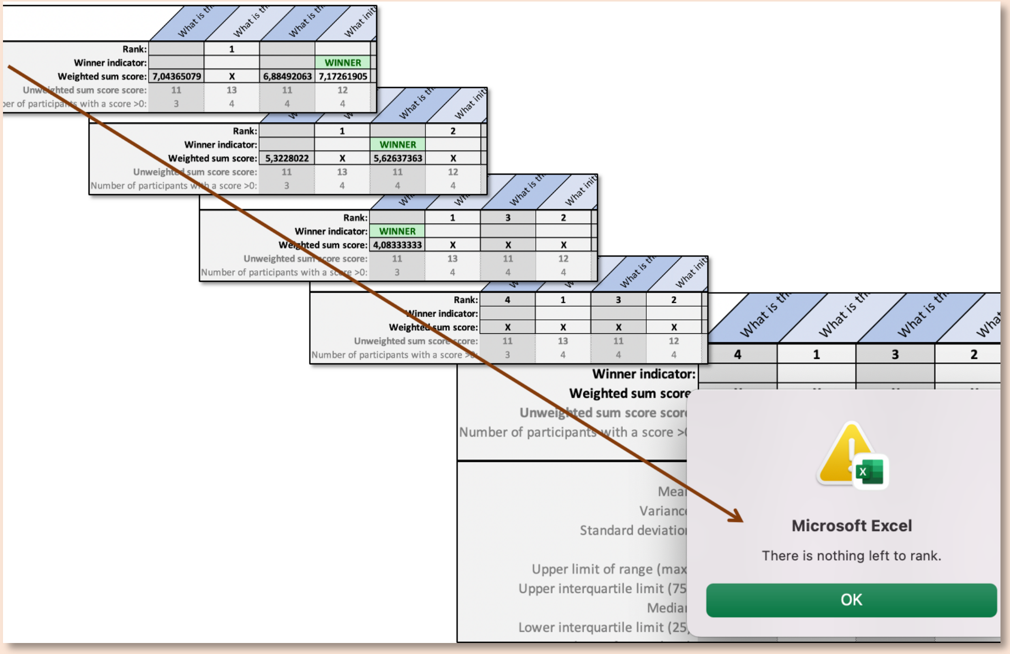

Figure 12 *– Semi-automatic ranking using the ‘Rank next –>’ button (Fig. 11) until there are no items left to rank, which prompts a message to inform the user.*

In some cases, the (new) sum scores of multiple items may be equal and these items are tied for a rank. Equal sum scores are most likely to happen during the first few assignments of ranks, whereafter it probably becomes less likely that items have exactly the same sum score due to the individual weighting of the assigned scores.

The REPS-tool will indicate which items have identical scores with ‘MULTI (n)’ in red when tied for a rank (Fig. 13). Here, *n* displays the number of items with identical scores. Clicking the ‘Next ranking –>’ button when multi-winners are identified will not assign a rank to any of these items. The ‘Next ranking –>’ button, however, prompts a message indicating for which rank the items are tied. This rank needs to be manually assigned to one of the multi-winner items. There are no prespecified rules for this decision yet, however heterogeneity analysis may aid in the

decision. For example, one might choose an item where there is some agreement among the participants reflected by a smaller variance, standard deviation, or range compared to the other multi-winner item(s).

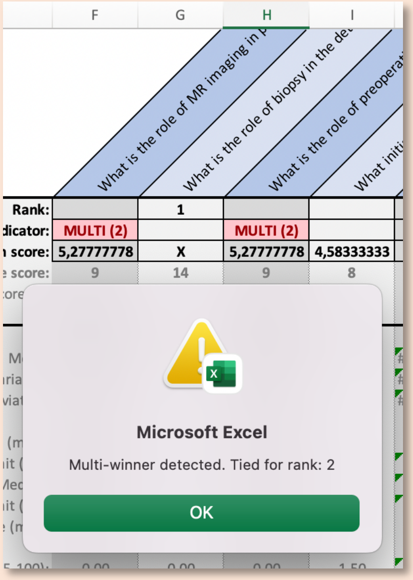

Figure 13 – Two items are tied for a rank as indicated by MULTI(2) in the tool. The REPS-tool identifies which items are tied by displaying MULTI(n) in the item column and shows how many items are tied within the parentheses. Clicking the 'Rank next –>' button (Fig. 11) will prompt a message showing for which rank the items are tied. In this case there is a tie for rank 2.

All items who received a rank will be displayed in the Ranking outcome worksheet ordered by their rank. When deciding to change the tool's parameters after a ranking is completed, the tool does not refresh the assigned ranks automatically. Thus, after changing the tool's parameters, the ranking should be cleared before new ranks are assigned according to the new parameters.

Note: Do not assign identical ranks to multiple items. These items will not be displayed correctly in the 'Ranking outcome' worksheet. For example, do not manually assign the same rank to the items identified as a multi-winner.

10. Heterogeneity analyses

The REPS-tool has a section for heterogeneity analyses (Fig. 2C). Analyses are carried out on the unweighted scores assigned by the participants. Central tendency measures (i.e. mean and median) are provided, accompanied by measures of dispersion (i.e. variance, standard deviation, range, and interquartile range) and sizes of the quartiles.

Items meeting specific conditions, such as a variance greater or equal to three, can be highlighted in the REPS-tool (Fig. 14). Fill out and accept a value in the yellow (smaller or equal to) or red (greater or equal to) cell right to 'Highlight when:' for the tool to automatically highlight items meeting the condition.

Heterogeneity	≤	≥					
			Mean:	2,75	3,25	2,75	3,00
Highlight when:		3,00	Variance:	3,69	0,69	1,69	4,00
Highlight when:			Standard deviation:	1,92	0,83	1,30	2,00
			Upper limit of range (max):	5,00	4,00	4,00	5,00
			Upper interquartile limit (75):	4,25	4,00	4,00	5,00
			Median:	3,00	3,50	3,00	3,00
			Lower interquartile limit (25):	1,50	2,75	1,75	1,00
			Lower limit of range (min):	0,00	2,00	1,00	1,00
	≤	≥					
Highlight when:			Size of the 4th quartile (Q4: 75-100):	0,75	0,00	0,00	0,00
Highlight when:	2,00		Size of the 'box' (Q2+Q3: 25-75):	2,75	1,25	2,25	4,00
Highlight when:			Size of the first quartile (Q1: 0-25)	1,50	0,75	0,75	0,00

***Figure 14** – Highlight items for central tendency and dispersion measure values 'smaller than or equal to' (yellow) and 'greater than or equal to' (red). Items meeting the condition are highlighted in their respective column.*

When items are tied for a rank and the rank has to be entered manually, heterogeneity analyses may be of use in deciding which item receives the rank. Although there is currently no consensus and there are no predefined decision-rules, the measures of dispersion might indicate the level agreement among participants. For example, large variance could mean that the participants do not agree about the priority while little variance could indicate that there is more agreement about the level of priority. One might choose, for example, to assign the rank to the item with the smallest variance.

10. Tool output

The output of the tool is a list of items ascendingly ordered by the assigned ranks to the items. Only items that were (manually or semi-automatically) assigned a rank to in the 'RRV' worksheet are displayed in the 'Ranking outcome' worksheet of the REPS-tool (Fig. 15). It is possible to create a top 10 or a top 15, by (manually or semi-automatically) assigning a rank to the first 10 or 15 winners, respectively. That means that solely items that had a rank assigned to them in the 'RRV' worksheet are ordered and displayed in list in the 'Ranking outcome' worksheet. The list can be copied from the worksheet to other applications, such as Microsoft Word.

	A	B
1	**RANK**	**NAME**
2	1	What is the role of biopsy in the detection of a he
3	2	What initial approach should be used in patients v
4	3	What is the role of preoperative portal vein embo
5	4	What is the role of MR imaging in patients with a
6	5	#N/B

Figure 15 *– The list of items that received a ranking ordered by rank in the 'Ranking outcome' worksheet. Microsoft Excel shows #N/B because a fifth item was not assigned a rank.*

11. Adapting the REPS-tool

The formulas in the worksheets and the worksheets themselves are not locked in the REPS-tool. Therefore, the tool can be programmed in Microsoft Excel according to specific needs not currently programmed in the tool. For example, it is conceivable to wish for a different weighting of individual weights for patients participating in the priority-setting assessment. Or, a different set of weighting methods could be programmed than currently available in the tool. Although these aspects are not features in the current version of the REPS-tool, it should be programmable using Microsoft Excel when desirable. The REPS-tool may be further edited by programming specific wishes for an organization in the tool. In fact, we would encourage any adaptations that would allow for its use for different contexts and needs. This includes priority-setting outside the context of clinical practice guidelines.

12. Reference list

1. Kok J, Smith WD. Re-weigted Range Voting - a Proportional Representation voting method that deels like range voting [cited 2023 15th of May]. Available from: https://www.rangevoting.org/RRV.html.
2. Smith WD. Reweighted range voting – new multiwinner voting method. 2005.
