# Supplementary material for: Introducing re-weighted range voting in clinical practice guideline prioritization: Development and testing of the re-weighted priority-setting (REPS) tool
Source: PLoS One. 2024 Apr 5;19(4):e0300619. doi: 10.1371/journal.pone.0300619 (PMC10997121; doi:10.1371/journal.pone.0300619)
Supplement: S6 File — This file contains figures depicting the priority-setting assessment in the Kleefstra guideline pilot mapped to our frame of reference and its final outcome. Furthermore, the output generated by the REPS-tool under four conditions are shown and compared. (DOCX) [file pone.0300619.s006.docx]

**Supporting information file 6 – Priority-setting in the Kleefstra syndrome guideline**

**S6 Fig 1. The priority-setting assessment in the Kleefstra syndrome guideline.** The priority-setting assessment (colored row) was used to prioritize 12 key questions for development in the Kleefstra syndrome guideline (see S6 Fig 2). The parallel assessment (grey row with colored outline) shows how input from the priority-setting assessment was used to test different four conditions in the REPS-tool (see S6 Fig 3), whereafter the Kleefstra guideline panel were asked which output they preferred.

**

**S6 Fig 2. The final outcome of the priority-setting outcome in the Kleefstra syndrome guideline.** A top-18 output of the REPS-tool was presented to the core group and discussions led to the final selection of 12 key question for development in the Kleefstra syndrome guideline. The figure shows which input and weighting method was used and which key questions were ultimately selected per subgroup in the guideline panel through discussing the REPS-tool’s output in the core group.

**

**S6 Fig 3. Four ranked top-10 outputs resulting from four conditions in the tool using input from the priority-setting assessment in the Kleefstra guideline.** The figure displays the four hypothetical options the Kleefstra syndrome guideline panel could indicate they preferred. Reading the figure from left to right: introduction of a new color in a column (i.e. light orange and mustard yellow) indicates the appearance of a key question now ranked in the top-10 which was not ranked in previous options. The crosslinks show how questions changed in ranking depending on the input format and weighting method.

**

**S6 Fig 4. Comparing the two hypothetical top-10 options most preferred by the Kleefstra syndrome guideline panel.** The figure shows two ranked top-10s obtained using different input formats and weighting methods. The purple highlighted boxes in Option 1 are key questions that did not appear in Option 4. The blue highlighted boxes in Option 4 are key questions that did not appear in Option 1. Six key questions appeared in both Option 1 and 4, with crosslinks showing the change in rankings between options. Note that Option 4 resulted in a #2 ranking of a question that did not appear in Option 1.
